# Patterns and rates of viral evolution in HIV-1 subtype B infected females and males

**DOI:** 10.1371/journal.pone.0182443

**Published:** 2017-10-18

**Authors:** Michael J. Dapp, Kord M. Kober, Lennie Chen, Dylan H. Westfall, Kim Wong, Hong Zhao, Breana M. Hall, Wenjie Deng, Thomas Sibley, Suvankar Ghorai, Katie Kim, Natalie Chen, Sarah McHugh, Lily Au, Mardge Cohen, Kathryn Anastos, James I. Mullins

**Affiliations:** 1 Department of Microbiology, University of Washington School of Medicine, Seattle, Washington, United States of America; 2 Department of Physiological Nursing, University of California at San Francisco, California, United States of America; 3 The Core Center, Bureau of Health Services of Cook County, Chicago, Illinois, United States of America; 4 Department of Medicine, Albert Einstein College of Medicine, Bronx, New York, United States of America; 5 Department of Medicine, University of Washington School of Medicine, Seattle, Washington, United States of America; 6 Department of Global Health, University of Washington School of Medicine, Seattle, Washington, United States of America; 7 Department of Laboratory Medicine, University of Washington School of Medicine, Seattle, Washington, United States of America; "INSERM", FRANCE

## Abstract

Biological sex differences affect the course of HIV infection, with untreated women having lower viral loads compared to their male counterparts but, for a given viral load, women have a higher rate of progression to AIDS. However, the vast majority of data on viral evolution, a process that is clearly impacted by host immunity and could be impacted by sex differences, has been derived from men. We conducted an intensive analysis of HIV-1 *gag* and *env-gp120* evolution taken over the first 6–11 years of infection from 8 Women’s Interagency HIV Study (WIHS) participants who had not received combination antiretroviral therapy (ART). This was compared to similar data previously collected from men, with both groups infected with HIV-1 subtype B. Early virus populations in men and women were generally homogenous with no differences in diversity between sexes. No differences in ensuing nucleotide substitution rates were found between the female and male cohorts studied herein. As previously reported for men, time to peak diversity in *env-gp120* in women was positively associated with time to CD4+ cell count below 200 (*P* = 0.017), and the number of predicted N-linked glycosylation sites generally increased over time, followed by a plateau or decline, with the majority of changes localized to the V1-V2 region. These findings strongly suggest that the sex differences in HIV-1 disease progression attributed to immune system composition and sensitivities are not revealed by, nor do they impact, global patterns of viral evolution, the latter of which proceeds similarly in women and men.

## Introduction

Previous studies have suggested that biological sex differences exist in the natural history and viral population genetics of HIV-1 infection. Women generally have higher CD4^+^ T cell counts and lower viral loads [1–4] but progress to AIDS at viral load levels half that of men [5]. HIV-1 subtype A and D infected women in Kenya were reported to have a high degree of viral genetic variation early in infection relative to their male counterparts [6]; yet, follow up studies found that upon exclusion of individuals with other sexually transmitted diseases, biological sex did not predispose individuals to the acquisition of multiple variants [7, 8]. During heterosexual transmission, viruses with a greater proportion of consensus residues at polymorphic sites were reported to be less frequently transmitted to male recipients compared to female recipients [9].

Sex-specific differences at the immunological and cellular level are also being explored to understand why women progress to AIDS at a faster rate then men. Cultured plasmacytoid dendritic cells (pDC) from women have increased sensitivity to HIV-1 RNA through innate Toll-like Receptor 7 (TLR7) [10, 11], leading to greater IFN-α production and, in turn, heightened CD8^+^ T cell immune activation—a strong predictor of disease progression [11, 12]. Moreover, T cells in women were found to produce elevated levels of IFN-stimulated genes (ISG) in response to IFN-α compared to male counterparts [13]. These studies suggest that the higher levels of immune activation in women may result in faster HIV-1 disease progression compared to men.

Viral evolution is in large measure driven by host immune responses, including a major impact from CD8+ T cells [14, 15]; however, only a limited number of studies have attempted to characterize patterns of viral evolution throughout the duration of HIV-1 infection. Shankarappa *et al*. was the first comprehensive, longitudinal study to accomplish this by studying *env-C2V5* evolution in a cohort of men who have sex with men (MSM) from the Multicenter AIDS Cohort Study (MACS) (*n* = 9) [16]. Therein, a root-to-tip linear regression measure revealed that viral divergence and diversity rates increased linearly throughout most of asymptomatic infection. Prior to the development of AIDS, viral diversification peaked and then declined, and divergence from the founding virus population stabilized. Although a recent analysis of 33 participants used similar methods to conclude that HIV-1 genetic diversity in *pro-pol* accumulates in a nonlinear fashion, this latter study was largely cross-sectional [17] and thus would have limited ability to detect the increases, plateaus and decreases found in a longitudinal study, especially since evolutionary rates are slower in the *pro-pol* region of the viral genome.

Within-host HIV-1 evolutionary rates are > 2-fold greater than population-level rates [18, 19], and several explanations have been posited to explain the differences [20]. Among these, the ‘store and retrieve’ mechanism, whereby ancestral virus is preferentially transmitted, appears likely [18–22]. Recent studies also report that population-level rates differ as HIV-1 spreads through certain risk groups. Vrancken *et al*. reported that evolutionary rates were lowest in heterosexual (HET) compared to MSM and intravenous drug users (IDU) [23], and the authors suggested that gender ratios within a given risk group may explain the associated evolutionary rate differences. Specifically, the incidence of multi-variant transmission is reported to be 2-fold higher in MSM [24] than in HET risk groups [7, 25, 26]. Despite these findings, no direct comparison between HIV-1 evolution in males and females has been performed.

The current study provides the first extensive longitudinal study of natural HIV-1 evolution in the oft-underrepresented female demographic and thus is the first to be extensively comparable to the Shankarappa *et al*. study. Men have been overrepresented in HIV studies to date, and while no good estimate for this exists one such metric from the HIV Los Alamos National Laboratory sequence database (http://www.hiv.lanl.gov) shows that sequences derived from men make up ~60% of the database, while women comprise over half of the infected population. Longitudinal viral sequences from the Shankarappa study and two additional MACS participants (*i*.*e*., M4 and M10) were included in this comparison [27, 28]. The unique longitudinal sampling of the current study and that of Shankarappa *et al*. permitted an interrogation of sex differences in HIV evolution *in vivo*. In addition, the current study in females (*n* = 8) enrolled in the Women’s Interagency HIV Study (WIHS) advances earlier findings from the MACS MSM cohort, assessing a 5-fold larger region of the viral genome, greater sequence numbers, and error-free sequence recovery due to use of consensus sequencing derived from single viral templates. To ensure that the analysis captured substantial ongoing virus-host immune interactions, sampling was limited to time intervals prior to initiation of combination ART. Our analysis reveals no significant differences in the nucleotide substitution rates between the male and female cohorts we studied with the caveat that these two cohorts had comparable set-point viral load (spVL) measures (atypical of established sex-based differences in HIV disease progression). As previously reported in men, we do find that the evolutionary metric time-to-peak-diversity is also associated with disease progression in women. Finally, we also investigate the heterogeneity of virus at earliest detected time and find no difference between the male and female cohort participants.

## Materials and methods

### Study participants

Plasma samples from eight adult, premenopausal women enrolled in the WIHS were used in this study. The WIHS is a prospective cohort that enrolls women with or at risk for HIV acquisition at six clinical research sites within the US [29]. The eight participants were selected to study the natural progression of HIV in women based on their being uninfected at enrollment and plasma sample availability from the initial HIV-1 PCR positive date until either: i) CD4^+^ T cells reached < 200/uL, or ii) initiation of sustained ART following a period of no treatment for ~6 or more years of infection. Samples were collected every 6 months for the first 2 years of infection, then annually until an aforementioned endpoint was met. Participants F1, F2, F5, and F6 received a single dose of ART early in infection but were not on sustained ART at any period of time during the sampling period. Participants’ self-reported race and acquisition risk category, and clinical measures of set-point viral load and time to CD4^+^ T cell count < 200/uL are shown in Table 1. The eleven male participants from the MACS were drawn from the cohort of 14 individuals described by Rinaldo [28] as reported [16, 27], using a plasma collection schedule analogous to that of the WIHS.

**Table 1:** Cohort description^a^.

PtID^b^	Race	Risk Category	Set-point viral load (cp/mL)	Time to AIDS^c^ (yr)
F1	African-American (Non-Hispanic)	None identified	8,300	6.3
F2	Other (Hispanic)	Intravenous drug use	42,000	5.0
F3	African-American (Non-Hispanic)	Heterosexual sex	41,000	5.3
F4	White (Hispanic)	None identified	860	4.1
F5	Other	Intravenous drug use	9,700	6.0
F6	African-American (Non-Hispanic)	Intravenous drug use	22,000	10.3
F7	African-American (Non-Hispanic)	Heterosexual sex	1,700	N/A^d^
F8	African-American (Non-Hispanic)	None identified	7,700	7.9

^a^All participants were from the US and infected with HIV-1 Subtype B.

^b^PtID = Participant Identifier.

^c^AIDS defined as CD4+ T cell numbers reaching below 200/mm^3^.

^d^N/A = not applicable, did not develop AIDS during the period of follow up.

### Ethics statement

Written informed consent was provided by all study participants. The study was approved by the following Institutional Review Boards: Albert Einstein College of Medicine, University of California at San Francisco, and Bureau of Health Services of Cook County.

### Clinical samples

Plasma HIV-1 RNA was measured by isothermal nucleic acid sequence based amplification (NASBA/Nuclisens; Organon Teknika Corp.) with a lower limit of detection of 80 copies/mL. Lymphocyte subsets were quantified using standard flow cytometric methods in laboratories participating in the NIH/NIAID Flow Cytometry Quality Assessment Program [30].

Set-point viral load was estimated as the average of plasma viral load (VL) measurements taken after acute infection (< 4 months) but prior to chronic infection (> 2 years). Measurements were excluded from this estimate if a previous measurement varied by > 1 log_10_ copies/mL.

Time to CD3^+^ T cell inflection was estimated using a segmented regression model of log_10_ CD3^+^ T cell counts [31] yielding the smallest residual variability and a constrained initial slope of 0. Estimated inflection points (IP) required at least three measures before and after a potential midpoint, and the midpoint date was the mean of the two dates surrounding the IP. Additionally, the CD3^+^ T cell count immediately following the midpoint was required to decrease.

### HIV-1 amplification and sequencing

Between 20–30 sequences were targeted for both full-length *gag* and *env-gp120* (unlinked) genes at each timepoint; however, low viral loads and exhaustion of samples limited success from 6 of 85 plasma samples. A total of 1,790 *gag* sequences (median = 22) and 1,755 *env-gp120* sequences (median = 21) were recovered for analysis. Note: Prior to final manuscript submission it was determined that one *gag* sequence (clone G28) out of 23 sequences in participant F4 timepoint 6 (3.6 years post seroconversion) was erroneously duplicated. It was included in subsequent analyses since it did not impact our results. For example, within timepoint diversity measures from this specimen were 0.3425% (standard error = 0.0101%) with the duplicated sequence included and 0.3425% (standard error = 0.0107%) with the duplicated sequence removed.

Briefly, RNA was extracted from 280uL aliquots of plasma with the QIAamp Viral RNA Mini Kit (QIAGEN, Inc.) cDNA synthesis was performed using either BluePrint 1^st^ Strand cDNA Synthesis Kit (Takara Bio, Inc.) or SuperScript III First-Strand Synthesis System for RT-PCR (Life Technologies, Thermo Fisher Scientific, Inc.) with HIV-1 specific primers: NEF3 [32] and RT2 [15]. cDNA was subjected to end-point dilution to allow single template amplification of viral cDNA with a target of 1 positive per 3–5 reactions, and then PCR products were sequenced directly to avoid detection of PCR misincorporation errors [33]. Nested PCR was employed on all cDNA templates with the majority of reactions using BIOLASE DNA Polymerase (Bioline USA, Inc.), while Advantage 2 (Clontech Laboratories, Inc.) was used for difficult-to-amplify samples. First round multiplex PCR reactions included the primers to cover *gag* [F683 (5’-CTCTCGACGCAGGACTCGGCTTG) and RT2 [15]; HXB2 (GenBank# K03455.1) positions: 683–3321] and *env* [ED3 [34] and NEF3 [32]; HXB2 positions: 5957–9038]. Thermocycling conditions were: 2 min at 94C; 5 cycles of 1 min at 94C, 30s at 55C, and 135s at 72C; 30 cycles of 15s at 94C, 30s at 58C, and 135s at 72C; followed by a 7 min extension at 72C and a 4C hold. *gag* and *env* regions were amplified separately in second-round PCRs using F762 (5’- TTGACTAGCGGAGGCTAGAAGGAGA) and RSP15R [35] (HXB2 positions: 762–2403) or gp120-5 [36] or gp120-3 [36] (HXB2 positions: 6205–7810) primers, respectively. Thermocycling conditions for 2^nd^ round reactions were: 2 min at 94C; 5 cycles of 1 min at 94C, 1 min at 55C, and 1 min at 72C; 30 cycles of 15s at 94C, 30s at 55C, and 1 min at 72C; followed by a 7 min extension at 72C and a 4C hold. Amplicons were purified by gel electrophoresis or on silica columns (NucleoSpin PCR Clean-up; Macherey-Nagel, Bethlehem, PA) and both strands sequenced using dye-terminator Sanger sequencing (Genewiz; Seattle, WA).

### Sequence analyses

Several safeguards were in place to eliminate cross-contamination with DNA from other sources in the laboratory and across timepoints within each participant: PCR setup was performed in a dedicated clean room absent of any PCR-amplified or plasmid DNA, and all *gag* and *env-gp120* sequence assemblies were regularly compared against the continually updated Mullins laboratory HIV sequence database using ViroBLAST [37] [http://indra.mullins.microbiol.washington.edu/viroblast/viroblast.php]. As it is particularly difficult to detect specimen mix-up or contamination in longitudinal studies, researchers also never worked with more than one specimen at a time from a given participant.

Sequence chromatograms were trimmed based on quality and to remove primer sequences, and then assembled into contigs within Geneious^®^ 7.1.7 (Biomatters, Auckland). Contigs were assessed for quality and complete forward and reverse coverage and then used to generate a consensus sequence. Up to 2 base ambiguities were permitted per assembled consensus sequence (≤ 0.13%).

Sequence alignments were generated using MUSCLE [38] within Geneious followed by manual editing. Hypermut 2.0 [39] was used to identify APOBEC3G/F hypermutated sequences, which were then eliminated from downstream analyses. The hypervariable regions within V1, V2, V4 and V5 were excluded from *env-gp120* alignments when used to assess positional homology for potential N-linked glycosylation site analyses; these included positions (HXB2 amino acid locations) 132–152, 185–190, 396–410, and 460–465, respectively. A founder sequence was inferred within each participant as the consensus sequence of the first available timepoint.

BEAST version 1.8.2 was used to estimate substitution rates [40]. Each taxa was dated according to the known time of sample collection. All sequence alignments were confirmed to possess sufficient temporal signal using TempEst [41]. The GTR +I +G substitution and site heterogeneity models were set for the nucleotide substitution model [42]. A lognormal relaxed molecular clock [43], which estimates the level of rate variation among lineages, was used instead of a strict molecular clock because formal model comparison testing revealed the relaxed clock a better fit of the data (data not shown). Prior distributions for the uncorrelated lognormal distributional model (for a relaxed clock) were specified as follows: the ucld.mean was gamma distributed with a mean of 0.075 and a shape of 1000, and the ucld.stdev was exponentially distributed with a mean of 1/3, initial value of 1/3, and offset of 0. This model yields an estimate of each branch-specific rate, as well as the coefficient of variation and covariance of rates across the tree. The coefficient of variation provides a measure of rate heterogeneity across branches and gives information about how clock-like the data is. The covariance measures autocorrelation between adjacent branches in the tree. Model testing was also performed for the tree prior; the exponential growth population coalescent model fit the data better than a constant population coalescent model (except for M1) and was used for all nucleotide substitution rate estimates [44, 45]. A comparison of nucleotide substitution rate estimates for each viral region is shown for constant and exponential growth population coalescent models (S1 Table). All other priors were left as default parameters. The operators were set to auto-optimize during the simulation. Chain lengths were set to 10^8^ with sampling every 10^4^ steps, which ensured a decent sampling from the posterior and effective sample size (ESS) > 100 for all meanRate estimates (ESS > 200 in > 90% of cases). Additionally, each BEAST simulation was run three times (each with a different random starting number) to verify similar convergence from run to run. A separate analysis with the CTMC reference prior on the mean clock rate showed no difference in meanRate estimates (data not shown). Output log files were analyzed using Tracer [http://tree.bio.ed.ac.uk/software/tracer/] and the mean of the posterior density was reported along with the 95% highest posterior density (HPD) interval. Hierarchical estimates of substitution rates were also employed in BEAST using the hierarchical phylogenetic model described by Edo-Matas *et al* [46]. This framework allows for different evolutionary histories of the within-host variants from individual-to-individual while providing an overall between-host summary estimate of nucleotide substitution rates. A fixed-effects HPM was used for hypothesis testing of the between group *env-C2V5* substitution rates from the 8 WIHS and 11 MACS participants.

jModelTest 2.1.7 [47] was used to determine the appropriate nucleotide substitution model for each sequence dataset, with GTR +I +G [48] chosen as it resulted in the highest likelihood scores for most alignments. Equations 1 and 2 from Deng *et*. *al*. [49] were used to compute average (± standard error) pairwise divergence and diversity, respectively, using the DIVEIN webtool (https://indra.mullins.microbiol.washington.edu/DIVEIN/).

Phylogenetic tree reconstruction by maximum likelihood was performed using PhyML v3.0 [50] within DIVEIN [49] for intra-participant alignments, and because of its fast maximum tree search algorithm, RAxML (Randomized Axelerated Maximum Likelihood) [51] was used to infer inter-participant phylograms. RAxML was also used to perform intra-participant *a posteriori* bootstrap convergence tests (S1 Fig). Phylogenetic inference was performed using a GTR substitution model, optimized equilibrium frequency, estimated proportion of invariable sites (I), and among site rate heterogeneity was captured using a discretized Gamma distribution. Tree searching optimization used the better of nearest-neighbor interchange (NNI) and subtree pruning and regrafting (SPR).

CodeML from the PAML v4.8a software package [52] was used to estimate nonsynonymous (*d*_*N*_) and synonymous site (*d*_*S*_) substitutions rates. CodeML estimated a single *d*_*N*_/*d*_*S*_ (**ω**) over the coding region using the M0 (one ratio) model. The hierarchical M7(β) and M8(β and ω) site-based models were used for hypothesis testing of positive selection at individual codon sites. Codon alignment and ML-based tree files (from PhyML inference) were input from the viral population sequences sampled for each participant at: i) each timepoint individually (model M0), or ii) for all timepoints collectively (model M0, M7, and M8). The following parameters for CodeML were changed from otherwise default values (CodonFreq = 2: F3x4; kappa = 1.6; fix_alpha = 1: fixed; ncatG = 3; fix_rho = 1: fixed; method = 1: one branch at a time; blength_fix = initial). A likelihood ratio test (LRT) was used to test for positive selection under the M7 and M8 models. Significance of the LRT was determined assuming a Chi square with 2 degrees of freedom test statistic distribution. A mixed effects model of evolution (MEME) [53] was used to infer codons undergoing episodic diversifying selection using the Datamonkey webserver [54]. This model combines fixed effects at the level of a site with random effects at the level of branches. Positively selected sites are reported for p-values < 0.05 using a LRT that tested the null vs. alternative models of nonsynonymous rate variation. A false discovery rate (q-value < 0.2, derived from the corresponding *P*-value using Simes’ procedure [55]) analysis was used to refine these inferences.

Co-receptor usage was predicted using the nucleotide position-specific scoring matrix (ntPSSM) of translated V3 loop amino acid sequences [27]. PSSM scores of -6 or greater were taken as indicative of X4-tropism [56]. Predicted N-linked glycosylation sites (PNLGS) were obtained using N-GlycoSite [57] [http://www.hiv.lanl.gov/content/sequence/GLYCOSITE/glycosite.html].

### Variant detection by digital droplet PCR (ddPCR)

Detection of specific variants was assessed from sequence alignments and in participant F5 by variant-specific ddPCR using the QX100^™^ Droplet Digital^™^ PCR System (Bio-Rad, Hercules, CA). Reactions contained 2x QX200^™^ ddPCR^™^ EvaGreen Supermix (Bio-Rad), 10uM variant-specific primer, 2uL of cDNA template, and H_2_O to a total volume of 22uL. Variant 1 primers were [5F7543 (5’- AAGGAGAAATTAGGTGTGTATCA) and 5R7639 (5’- CCTCCAGGTCTGAAGATTTCA); HXB2 positions: 7543–7639] and variant 2 primers were [5F7545 (5’- GGACGAATTAGCTGTACATCAA) and 5R7631 (5’- TCTGAAGGTCTCATTCATGGA); HXB2 positions: 7545–7631]. Thermocyclying conditions were: 5 min at 95C; 35 cycles of 30s at 95C, 30s at 56C, and 1 min at 60C; hold at 4C. Primer specificity was shown by assaying variant 1 primers with a variant 2 positive control sequence and vice versa. Each ddPCR assay was performed in duplicate and the data provided is representative of two independent experiments.

### Graphics and statistical analyses

Graphics were generated using either GraphPad Prism v6.0f (GraphPad Software, Inc.) or R v3.1.1 [58]. Phylogenetic trees were generated in FigTree v1.4.1 (http://tree.bio.ed.ac.uk/software/figtree/) and the phytools R package [59]. The nonparametric Mann-Whitney U test was used to test for differences between unmatched groups. Most associations were assessed using a nonparametric Spearman’s rank correlation coefficient (ρ) test because the relatively small sample sizes obtained were not assumed to be normally distributed. A Pearson’s correlation test was used to assess the relationship of larger sample sizes and the accompanying *r* coefficient and *P-*value was reported. A linear mixed effects model [58] was fit to the data and used to estimate the mean upward slope for differences in *d*_*S*_ and *d*_*N*_ rates over time. A likelihood-ratio test was used to compare the estimated rates within each model and to calculate *P*-values. Models were corrected for autocorrelation within the repeated measure datasets. LRTs revealed that the slope parameters (*d*_*S*_ / year and *d*_*N*_ / year) were best estimated with a random effects parameter, while the y-intercepts were best estimated with a fixed effects parameter (data not shown).

### Nucleotide sequence accession numbers

Nucleotide sequences are available from GenBank under the accession numbers MF777047—MF780582. Sequence alignments can be obtained at https://mullinslab.microbiol.washington.edu/publications/.

## Results

### Low diversity early in infection and consistent viral evolution between sexes

Plasma virus from all of the 8 WIHS participants who were infected with HIV while in longitudinal follow up and then followed for at least 6 years before ART were enrolled in this study. Blood samples were obtained for study from a median of 11 times per individual, usually at biannual visits, over a 6–12 year range. A median of 22 (range 2–28) ~1.6 kb *gag* and 21 (range 0–39) ~1.6 kb *env-gp120* single template-derived consensus sequences were obtained per timepoint (S2 Table).

**Fig 1:**
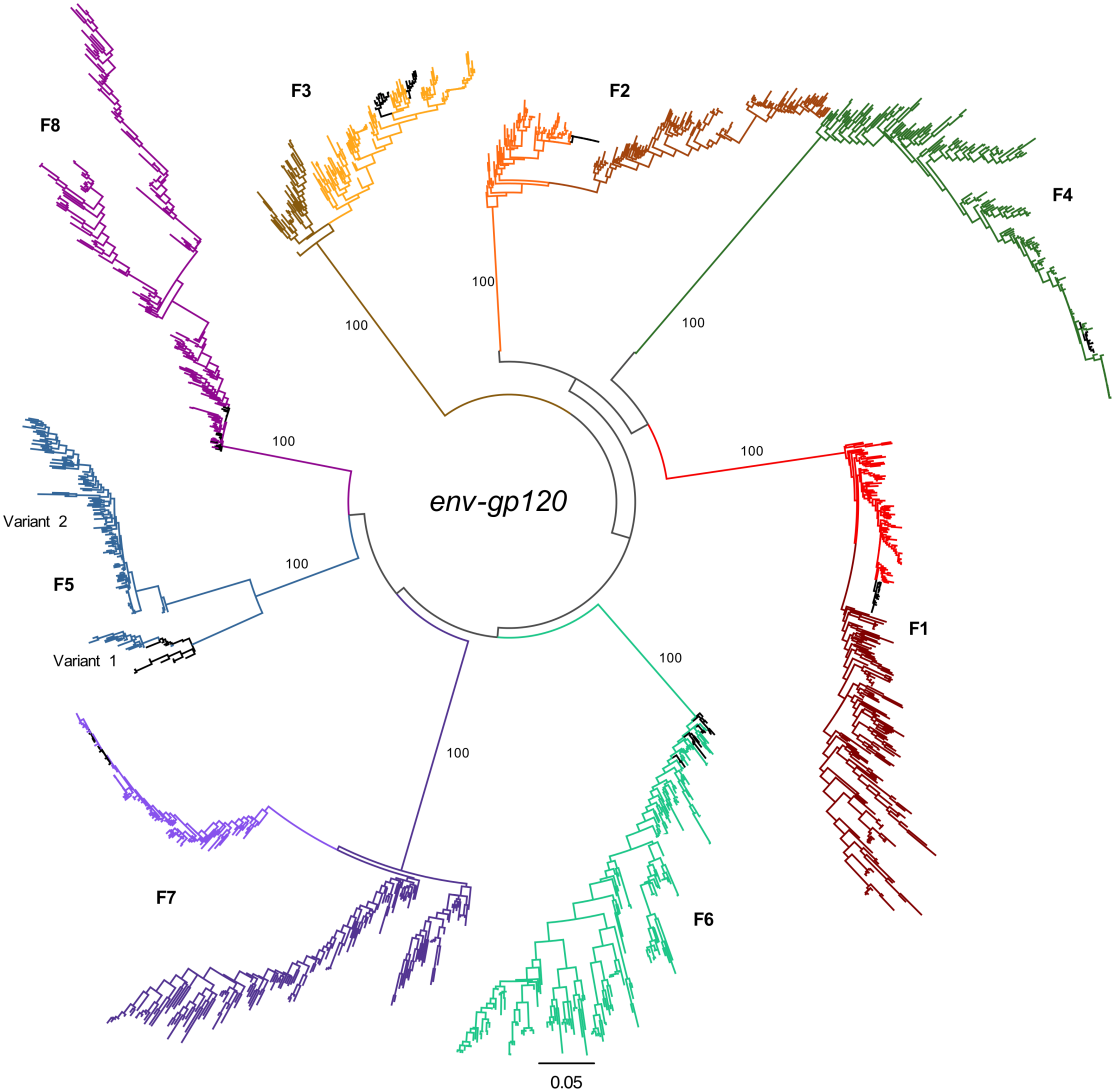
Inter-participant *env-gp120* phylograms. (**A**) A Phylogenetic tree of *env-gp120* for all 8 WIHS participants was inferred using RAxML [51] with the GTR substitution model +I +G [48]. External branches from the first available timepoint after infection are colored black. Branches from participants F1, F2, F3, and F7 are shaded light and dark to indicate taxa from early and late infection, respectively, when sequences from early in infection are found at opposite sides of the root node. The scale at the bottom measures genetic distances in nucleotide substitutions per site. Phylograms from each individual were rooted based on outgroup. Bootstrap values are shown along branches extending to each participant’s clade.

Inter-participant phylogenetic trees revealed possible acquisition of mutations over the first few years of infection that make sequences appear to become more closely related to the population most recent common ancestor (MRCA) in all except participants F6 and F8 in *env-gp120*. Sequences then diverge away from the MRCA at latter timepoints in 5/8 women (all except F4, F6, and F8; Fig 1; branches from the first timepoint are colored black; branches from early timepoints are shaded in lighter colors and latter timepoints are shaded in darker colors). Evolution away from the MRCA was strictly observed for F6 and F8 *env-gp120*, while continuous evolution toward the MRCA was observed for F4 *env-gp120* (Fig 1). This was also observed for 5/8 participants (all except F4, F5, and F8) in the *gag* phylogram (S2 Fig) and was also previously observed for individuals within the MACS cohort [60]. While the branch lengths separating sequences from different individuals are long, and may result in rooting artefacts, such topologies are consistent with the acquisition of reversion of immune escape mutations acquired from the transmitting partner in the early years of infection [61–66]. Further departure from the MRCA then appears to dominate, due to host-specific escape mutations.

Selective sweeps of the virus population in men, driven primarily by CTL pressure, begin to be recognized at one or a few positions in the viral proteome during the first 2 months of infection [67]. Since these women had blood draws only approximately every 6 months, as part of the WIHS protocol (median time to infection was estimated to be 91 days), we cannot rule out that a small number of sites would have undergone mutation and a selective sweep by the time of sampling. However, sequence analysis revealed the presence of near homogenous virus populations at the earliest time points sampled from 7 of the 8 participants (S3 Fig). Two highly divergent variant *env-gp120* lineages, including one that was atypically diverse (Variant 1), were initially found in participant F5 (Fig 1 and S4 Fig). Alignment of the *env-gp120* sequences from F5 with 2,200 epidemiologically unlinked subtype B sequences showed that the two variants were phylogenetically linked (S5A Fig). This indicates the outgrowth of multiple variants from a single infecting donor rather than the two variants originating from different donors. Variant-specific droplet digital (dd)PCR showed the proportion of each variant varied over time, with variant 1 dominating initially, and then fluctuating until being substantially replaced by variant 2 by 6.2 years post infection (S5B Fig). Single template amplification followed by Sanger sequencing detected recombinants between the two variant populations at the 0.8 year time point only (S4 and S5C Figs). ddPCR was used to amplify a ~100bp region and thus was unable to quantify recombinants.

**Fig 2:**
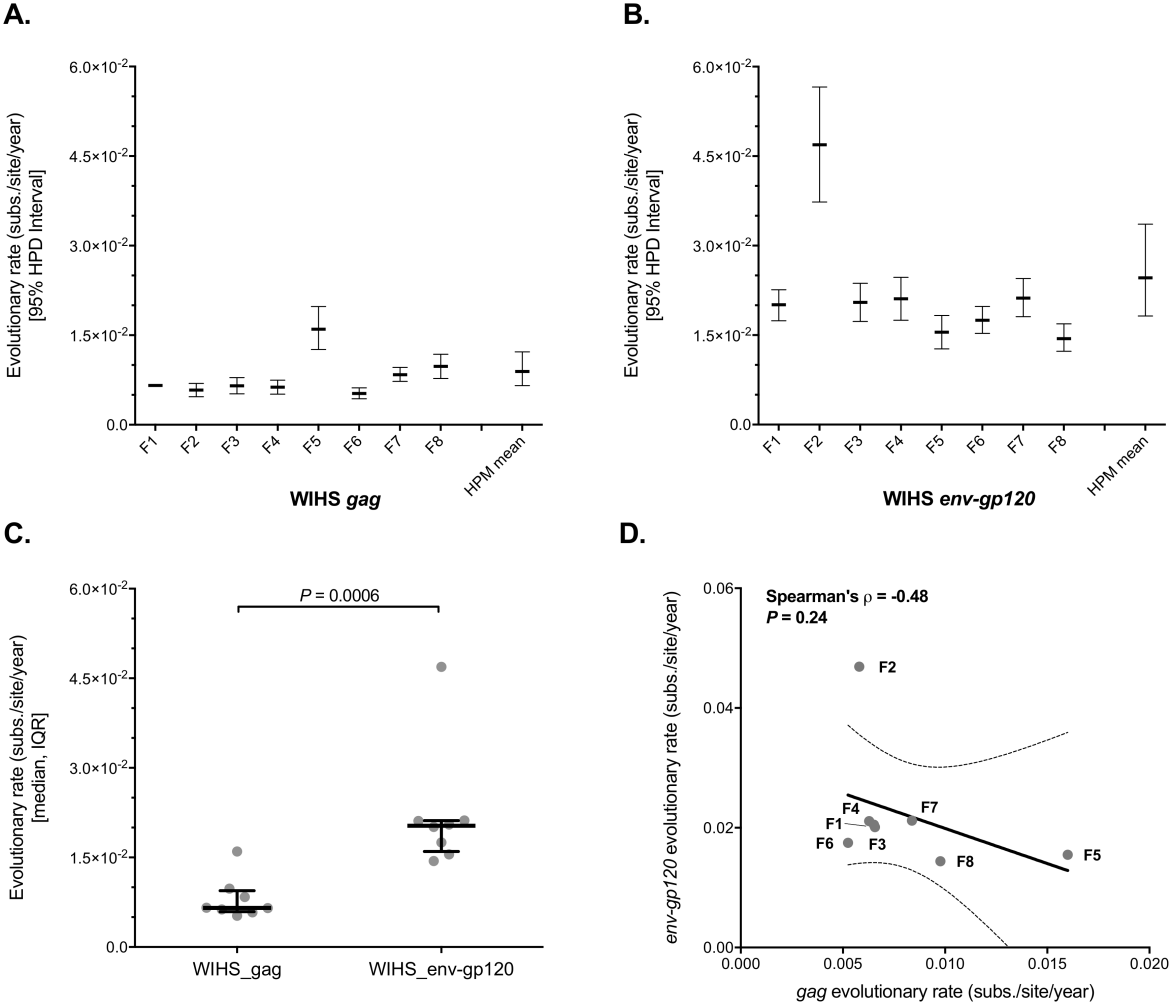
Nucleotide substitution rates within HIV-1 subtype B *gag* and *env-gp120*. Intra-host viral evolutionary rates within *gag* (**A**) and *env-gp120* (**B**) were estimated using a relaxed molecular clock model within a Bayesian framework for all WIHS participants (see Methods). 95% HPD is the highest posterior density interval. HPM is the mean evolutionary rate (and 95% HPD) estimated using a hierarchical phylogenetic model applied across the group. Evolutionary rates were defined as nucleotide substitutions/site/year. (**C**) A summary of the median and interquartile ranges (IQR) across participants. (**D**) The association between *gag* and *env-gp120* intra-host substitution rates. A non-parametric Mann-Whitney U test was used to test for differences between unmatched groups. Associations were analyzed using the Spearman’s correlation test; rho and *P*-values are shown. Dashed lines show the 95% confidence band of the best-fit line. ESS values were > 200 for meanRate in the analysis of 8/8 *gag* and 7/8 *env-gp120* BEAST simulations.

Nucleotide substitutions rates for *gag* and *env-gp120* were estimated within a phylogenetic context using Bayesian inference methods in the BEAST (Bayesian Evolutionary Analysis of Sampling Trees) [40] program. Evolutionary rates varied over roughly a three-fold range across participants, ranging from 5.25x10^-3^–1.60x10^-2^ (substitutions/site/year) with a median of 6.54x10^-3^ in *gag* (Fig 2A and S3 Table) and ~3-fold faster in *env-g120* (median of 2.03x10^-2^, range 1.44x10^-2^–4.69x10^-2^ subs./site/year) (Fig 2B and 2C and S3 Table). Hierarchical modeling supported this difference, as *gag* and *env-gp120* 95% credible intervals were non-overlapping. Mean across-group HPM evolutionary rate estimates were 8.92x10^-3^ (substitutions/site/year; [95% HPD = 6.56x10^-3^, 1.22x10^-2^]) in *gag* and 2.46x10^-2^ (substitutions/site/year; [95% HPD = 1.82x10^-2^, 3.36x10^-2^]) in *env-gp120* (Fig 2A and 2B). No association was found between intra-patient *gag* and *env-gp120* substitution rates (Spearman’s ρ = -0.48; *P* = 0.24) (Fig 2D).

**Fig 3:**
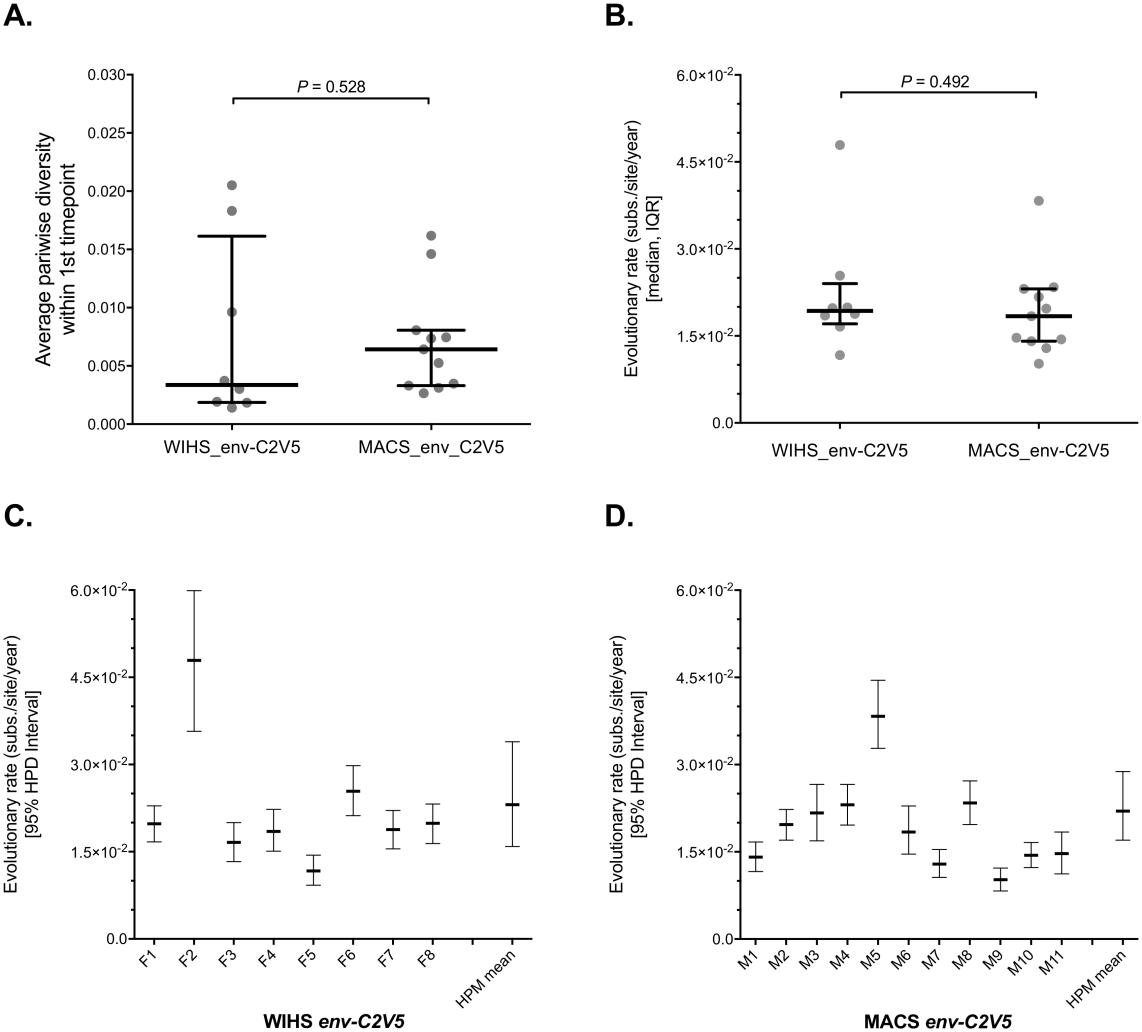
No differences in HIV-1 subtype B *env-C2V5* evolutionary rates between females and males. Intra-host *env-C2V5* (**A**) diversity at the first time point and (**B**) substitution rates for both WIHS and MACS cohorts. Substitution rates for both WIHS and MACS participants were estimated using a relaxed molecular clock model using Bayesian inference (see Methods). A Mann-Whitney U test was used to test for sex differences. Horizontal bars show the median and interquartile range. 95% HPD is the highest posterior density interval. HPM mean is the evolutionary rate (and 95% HPD) estimated using a hierarchical phylogenetic model applied across the group. Evolutionary rates were defined as nucleotide substitutions/site/year. A non-parametric Mann-Whitney U test was used to test differences between unmatched groups. (**C** and **D**) Evolutionary rates from (B) broken out by individual. ESS values were > 200 for meanRate in the analysis of 8/8 *env-C2V5* WIHS and 10/11 *env-C2V5* MACS BEAST simulations.

Early viral diversity and evolutionary trends from the WIHS cohort (*n* = 8) was compared to an existing cohort of men from the MACS that were also infected with HIV-1 subtype B (*n* = 11) [16, 27]. Previous MACS participant identifiers: Pt. 1 –Pt. 11 were renamed in this study to M1 –M11, respectively. Initial sampling times were similar between cohorts with a median of 91 [range: 73–285] and 102 [range: -77–416] days post seroconversion for the women and men, respectively. Comparing only the *C2-V5* regions of *env* (the only region available for the men), average pairwise diversity at the initial sampling time was not different between women and men (0.34% and 0.64%, respectively; *P* = 0.528, Mann-Whitney U test) (Fig 3A). Additionally, substitution rates estimated using BEAST were not significantly different between the sexes, as the median *env-C2V5* nucleotide substitution rate in women was 1.93x10^-2^ and 1.84 x10^-2^ in men (*P* = 0.492, Mann-Whitney U test) (Fig 3B–3D and S3 Table). Fixed-effects hierarchical modeling also showed that mean substitution rates were not different between the female and male cohorts (Fig 3C and 3D). The mean across-group HPM evolutionary rate estimate in *env-C2V5* was 2.31x10^-2^ (substitutions/site/year; [95% HPD = 1.59x10^-2^, 3.39x10^-2^]) in the WIHS cohort and 2.20x10^-2^ (substitutions/site/year; [95% HPD = 1.70x10^-2^, 2.88x10^-2^]) in the MACS cohort.

The coefficient of rate variation was estimated to assess the relative variability of the viral substitution rates in men and women. The median coefficient of rate variation within *C2V5* was not different, being 0.54 (range: 0.22–1.09) within the WIHS and 0.78 (range: 0.49–0.97) within the MACS (*P* = 0.319, Mann-Whitney U test) (S3 Table). The covariance of rates was estimated to measure how similar a given branch rate is to its ancestral or descendent branches. Again, the median covariation of rates was not different between WIHS and MACS participants, 0.024 (range: 0.0032–0.068) vs. 0.042 (range: 0.014–0.076), respectively (*P* = 0.506, Mann-Whitney U test) (S3 Table).

### Evolutionary patterns in env-gp120 are associated with disease progression in women as well as men

**Fig 4:**
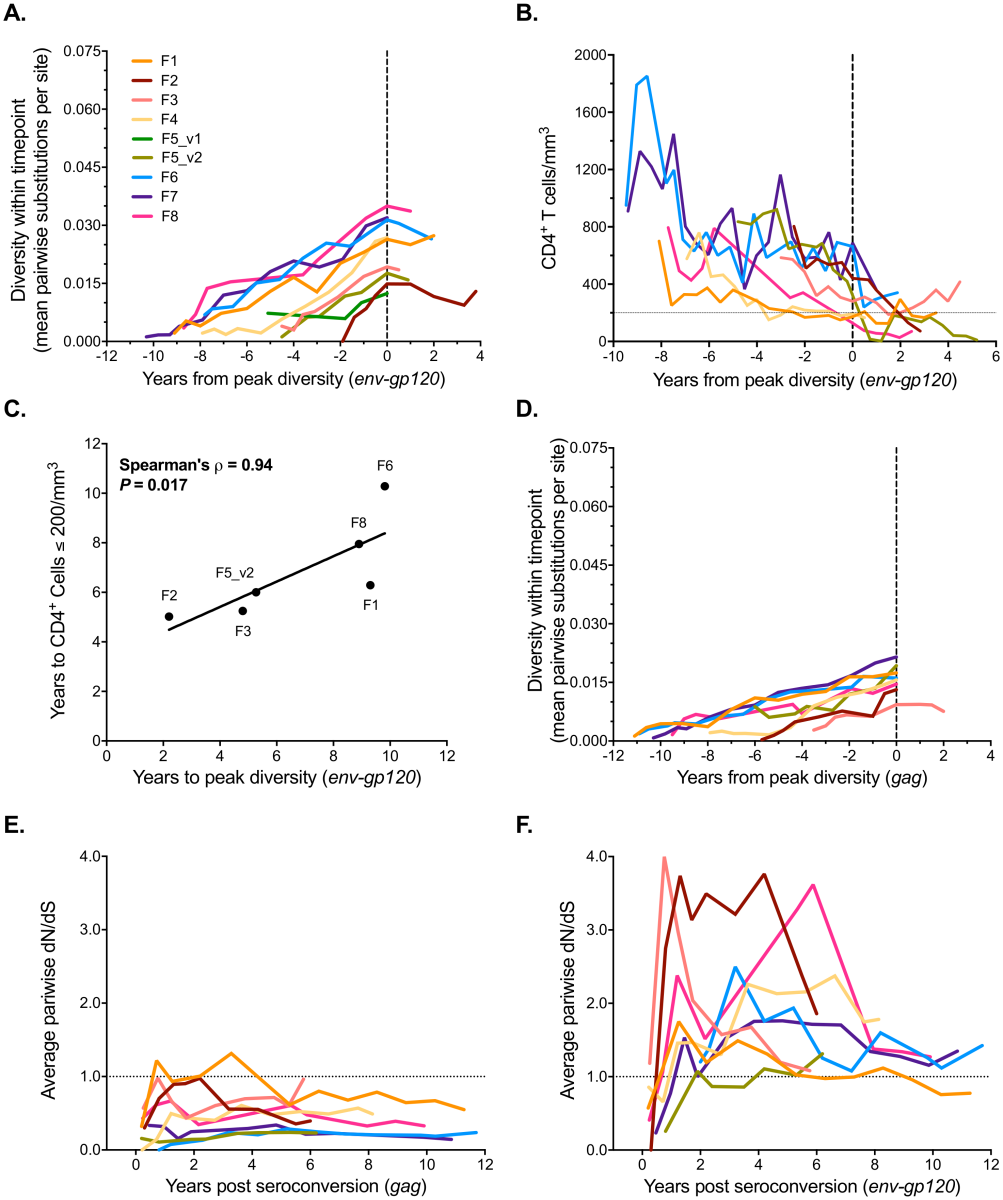
Correlates of disease progression and patterns of selection in the WIHS cohort. (**A**) Average pairwise diversity in *env-gp120* was estimated for each timepoint and is shown relative to peak diversity in each participant. (**B**) CD4^+^ T cell counts were placed relative to the time to peak diversity in *env-gp120*. The dashed horizontal line indicates the 200 CD4^+^ T cell count per mm^3^ AIDS-defining threshold. (**C**) The association between the time CD4+ T cells dropped below 200 per mm^3^ and the time of peak *env-gp120* diversity. Participants F4 and F7 were not included as virus had no observable peak in average pairwise diversity during the period of follow up. (**D**) Average pairwise diversity in *gag* for each timepoint is shown relative to the time of peak diversity in each participant. No peak was observed except for Subject F3. (**E**, **F**) The average pairwise ratio of nonsynonymous (*d*_*N*_) to synonymous (*d*_*S*_) substitutions per site at each timepoint compared to the inferred founder strain (see Methods section) for *gag* and *env-gp120*, respectively, for each participant. Associations were assessed using the Spearman’s correlation test.

**Fig 5:**
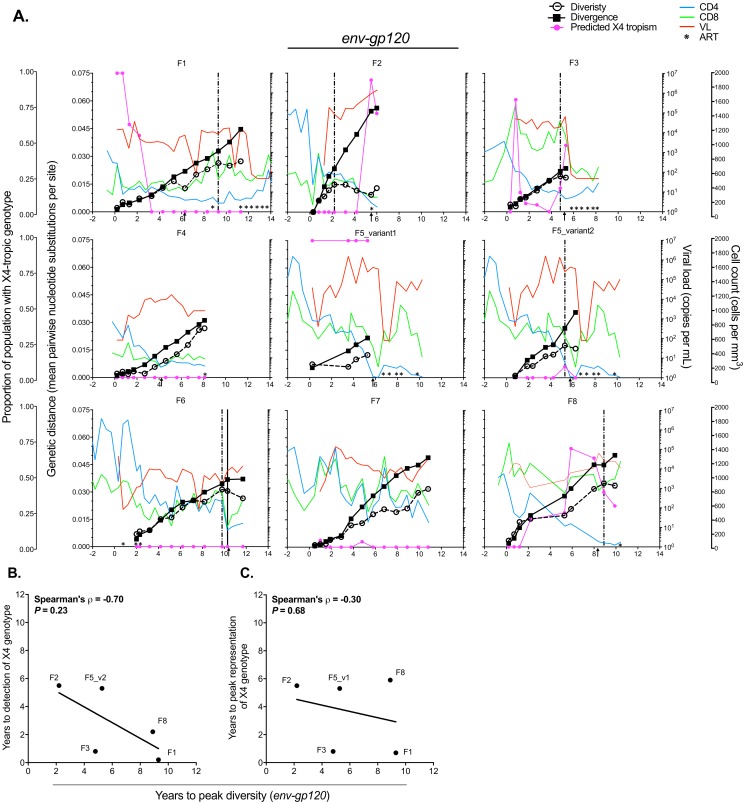
HIV-1 genetic distance measures over time. (**A**) Average pairwise nucleotide diversity within timepoints (open circles), divergence from the consensus of the initial timepoint sequences (filled squares) was calculated for *env-gp120* nucleotide sequences. Mean ± standard error is plotted (error bars are not visible as they were not as large as the data points). The proportion of predicted X4-tropic strains (filled magenta circles) computed by the PSSM scoring algorithm from V3 loop sequences is shown at each timepoint. The two distinct variants (1 and 2) within participant F5 were analyzed separately. HIV viral RNA load (copies per mL; red lines), CD4^+^ and CD8^+^ T cell counts (cells per mm^3^; blue and green lines, respectively), and visits with prescribed ART (black asterisks (*) at the bottom of each panel) are shown. The arrow at the bottom of each panel indicates the first time at which CD4^+^ T cell counts fell below 200. Dashed vertical lines indicate the time of peak viral diversity, when detected, while the solid vertical line indicates the time at which divergence from the initial consensus sequence stabilized (only detected in F6). Time to peak *env-gp120* diversity is shown associated with time to predicted X4-tropic genotype detection (**B**) and time to peak X4-tropic genotype representation (**C**). PSSM scores of ~ -6 or greater were taken as indicative of X4-tropism [56]). Associations were analyzed using the Spearman’s correlation test; rho and *P*-values are shown. Lines were fit using a least squares linear regression model.

The Shankarappa study showed that viral diversity grew in men until 3–4 years prior to the development of clinical AIDS, after which time diversity typically began to decrease [68]. The WIHS cohort evaluation was conducted through the time that cART became available, and thus none of these individuals reached terminal stages of disease, in contrast to the MACS study, which followed participants infected approximately a decade earlier, with several developing AIDS before effective ART was available. Hence, follow up in the WIHS cohort in the late stages of disease was more limited. Nonetheless, time to peak diversity in *env-gp120* could be estimated for WIHS participants (Figs 4A and 5A), and similar to the findings of Shankarappa *et al*. [16], time to peak diversity was positively associated with time to CD4^+^ T cell count < 200 for *env-gp120* (Spearman’s ρ = 0.94, *P* = 0.017) (Fig 4B and 4C) and C2-V5 (Spearman’s ρ = 0.83, *P* = 0.058) (S6A–S6C and S7A Figs). Little evidence of peak diversity, and therefore no association with this disease correlate, was observed in *gag* (measured only in the WIHS) (Fig 4D and S7B Fig).

The time to peak diversity in *env-gp120* was not correlated with time to initial emergence of a predicted CXCR4-tropic genotype (Spearman’s ρ = -0.70, *P* = 0.23; Fig 5A and 5B), nor with time to peak representation of predicted CXCR4-tropic genotypes (Spearman’s ρ = -0.30, *P* = 0.68) (Fig 5A and 5C). Participants F4 and F7 were excluded from these analyses because they exhibited no peak in diversity (Figs 4A and 5A). Participant F6 was also excluded because no predicted X4-tropic viruses were detected within her (Fig 5A and S8 Fig). Similar results were found for analysis of the C2V5 region alone (S6D and S6E and S7A Figs). In contrast, prior results in men found the time to emergence of the X4 genotype to be correlated with peak viral population diversity [16].

Another predictor of disease progression is loss of CD3^+^ T cell homeostasis [16, 28, 31, 69–72], thus, CD3^+^ T cells counts were fit to a segmented linear regression model to estimate an inflection point (IP) [31] in each participant (S9A–S9H Fig). There was a positive trend between CD3^+^ T cell IP and multiple other markers, although not significant at the *P* = 0.05 level, including: time to peak diversity (Spearman’s ρ = 0.44, *P* = 0.22), time to CD4^+^ T cell count < 200 (Spearman’s ρ = 0.55, *P* = 0.21), time to detection of the X4-tropic genotype (Spearman’s ρ = 0.09, *P* = 0.77), and time to peak representation of the X4-genotype (Spearman’s ρ = 0.71, *P* = 0.14) (S9I–S9L Fig).

To assess trends in selection over time within *gag* and *env-gp120* across the gene region in its entirety and at specific sites, the ratio between nonsynonymous (*d*_*N*_) and synonymous (*d*_*S*_) substitution rates were determined at each visit (Fig 4E and 4F, respectively) and all visits combined (Table 2), respectively. Positive selection was observed at specific codons in both *gag* and *env-gp120* (median of 4.5 and 28.5 sites under positive selection after false discovery rate analysis, respectively) (Table 2 and S10A and S10B Fig). Selection was also analyzed across the C2V5 region for sequences within WIHS and MACS participants and the median number of positively selected sites within each cohort was not different (median of 15 vs. 12, respectively; *P* = 0.318, Mann-Whitney test) (S10C and S10D Fig).

**Table 2:** Summary of nonsynonymous and synonymous substitution rates, site-based positive selection, and likelihood ratio test scores^a^.

		Number codons	*d*_*N*_^b^	*d*_*S*_^c^	*d*_*N*_/*d*_*S*_ (ω)		No. pos. sel. codons^d^	No. pos. sel. codons post-FDR^e^	LRT score^f^	P-value^g^
*gag*	F1	428	0.42	1.78		0.23	14	11	151	<0.0001
	F2	457	0.20	1.07		0.19	5	3	94.0	<0.0001
	F3	464	0.20	0.95		0.21	5	5	88.5	<0.0001
	F4	467	0.22	1.21		0.18	4	0	45.8	<0.0001
	F5	464	0.23	1.72		0.14	5	4	33.8	<0.0001
	F6	452	0.22	2.20		0.10	7	6	76.9	<0.0001
	F7	450	0.43	3.12		0.14	10	9	108	<0.0001
	F8	459	0.76	1.21		0.63	2	1	435	<0.0001
**Median**		**458**	**0.23**	**1.47**		**0.18**	**5**	**4.5**		
*env-gp120*	F1	389	1.39	2.89		0.49	60	45	643	<0.0001
	F2	380	0.74	1.24		0.60	27	27	497	<0.0001
	F3	461	0.66	1.11		0.59	29	29	583	<0.0001
	F4	485	0.86	1.05		0.82	28	28	828	<0.0001
	F5	478	0.37	0.83		0.44	13	10	182	<0.0001
	F6	417	1.01	1.53		0.66	48	48	713	<0.0001
	F7	363	0.82	2.64		0.31	40	40	264	<0.0001
	F8	431	0.24	1.92		0.13	25	16	54.0	<0.0001
**Median**		**424**	**0.78**	**1.38**		**0.54**	**28.5**	**28.5**		
Difference *gag* vs. *env-gp120*			0.55	0.084		0.36				
(P-value)^h^			(0.010)	(0.959)		(0.038)				

^a^LRT estimated in PAML using CodeML with hierarchical M7 (β) and M8 (β and ω) site models.

^b^Estimated nonsynonymous substitutions per site within coding region.

^c^Estimated synonymous substitutions per site within coding region.

^d^Number of positively selected codon sites determined by the mixed effects model of evolution method (see Methods) for p-values < 0.05.

^e^False discovery rate determined by q-value < 0.2 for independent tests, derived from corresponding p-value using Simes’ procedure.

^f^Likelihood ratio test score of positive selection determined by 2ΔlnL (2*(lnL M7—lnL M8). lnL is log-likelihood estimate.

^g^Determined assuming a chi square distribution with 2 degrees of freedom.

^h^Determined by Mann-Whitney U test.

At the gene-level, purifying selection (*d*_*N*_/*d*_*S*_ <1) was primarily observed for *gag* (Fig 4E), whereas positive selection (*d*_*N*_/*d*_*S*_ >1) was observed for *env-gp120* when each timepoint was evaluated separately (Fig 4F). Nonetheless, when summarized across the whole gene and over the entire observation period, purifying selection was observed for both genes, although more so in *gag* than *env-gp120* (median *d*_*N*_/*d*_*S*_ (or **ω**) = 0.18 vs 0.54, respectively; *P* = 0.038) (Table 2). *d*_*S*_ rates did not differ between *gag* and *env-gp120* when the visits were analyzed separately (0.49% vs. 0.46% per year; *P* = 0.794) (S11A–S11C Fig), or when summarized over the entire study period (median 1.47% vs 1.38%; *P* = 0.959, Mann-Whitney test) (Table 2). In contrast, *d*_*N*_ rates were significantly higher in *env-gp120* compared to *gag* when the visits were analyzed separately (0.55% vs 0.16% per year, respectively; *P* = 0.001) (S11A, S11B and S11D Fig) or combined (0.78 vs 0.23, *P* = 0.010; Mann-Whitney test) (Table 2). Estimations of *d*_*N*_/*d*_*S*_ ratios in the WIHS and MACS participant *C2V5* regions was also analyzed over time (S11E and S11F Fig), with patterns consistent with *env-gp120* (Fig 4E and 4F). Overall, the *d*_*N*_/*d*_*S*_ patterns were consistent with directional selection, whereby a period of mutation accumulation in multiple lineages is followed by fixation and purifying selection. However, as the assessment of *d*_*N*_/*d*_*S*_ ratios at each time point separately does not measure selection occurring on the branches connecting the populations, future work is still needed to assess the temporal changes in selective pressure.

**Fig 6:**
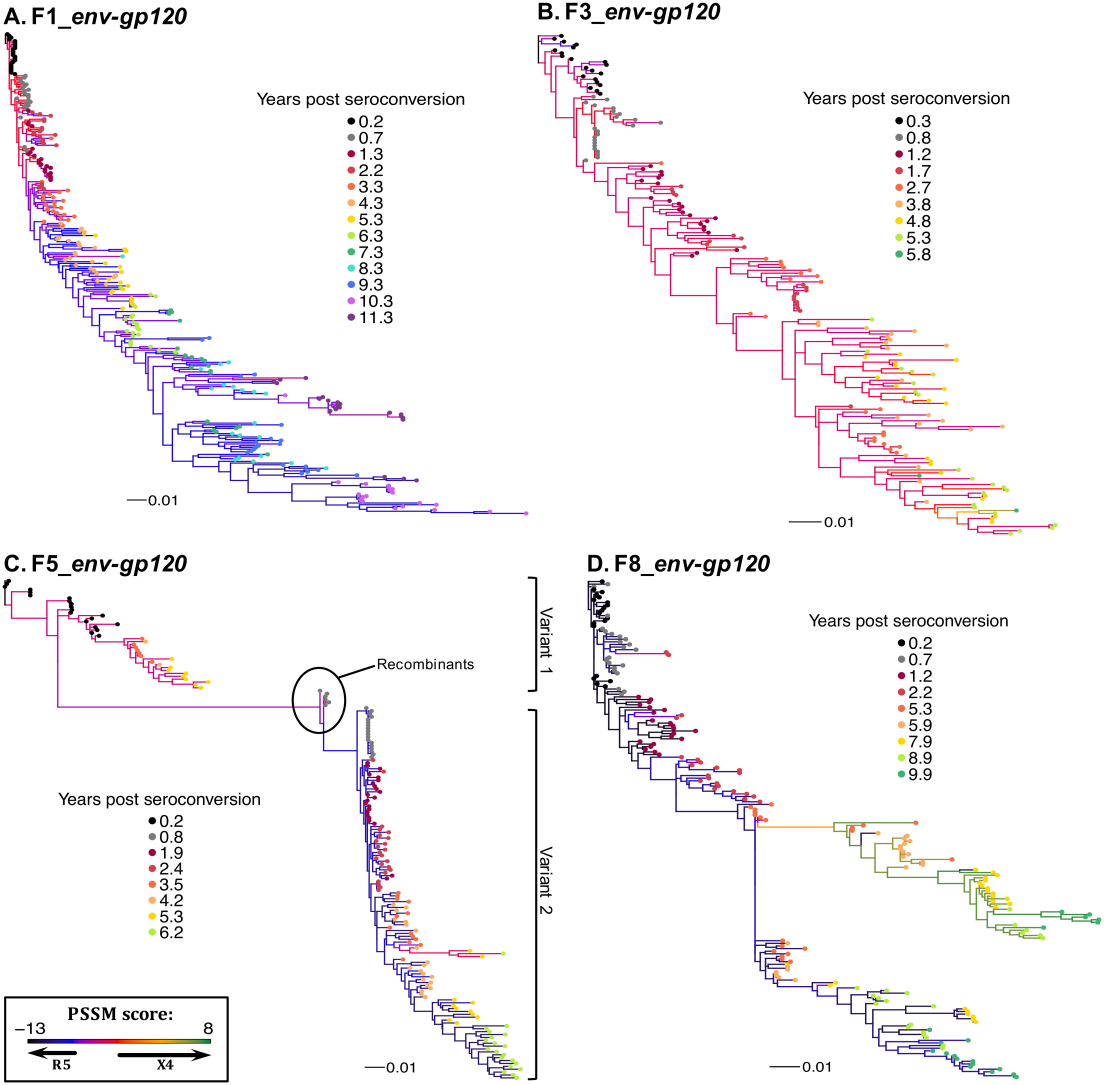
Phylogenetic analysis of *env-gp120* nucleotide sequences and predicted co-receptor use. (**A-D**) Maximum likelihood *env-gp120* phylogenetic trees from each participant were reconstructed using PhyML v3.0 (see Methods) and rooted to earliest timepoint sequences. Data from 4 participants are shown here and from the remaining four in Fig Sx. Tip symbols indicate years post seroconversion (colored circles). Branch colors represent PSSM-predicted V3 loop co-receptor usage (see Methods). The scale at the bottom measures genetic distances in nucleotide substitutions per site.

Phylogenetic trees were inferred using a maximum likelihood based approach and, based on the clustering of terminal nodes with respect to time, showed time-ordered relatedness for *env-gp120* (Fig 6) and *gag* (S12 Fig). The *env-gp120* phylograms also show predicted CXCR4/CCR5 co-receptor usage based on PSSM score (Fig 6; indicated by branch color). These data show that X4-tropic viruses (red/orange branches) were present in participant F1 at the first virus positive visit, and persisted for ~2.2 years. R5-tropic virus (violet/blue branches) was detected subsequently and persisted throughout infection (Figs 5A and 6A). *De novo* evolution toward X4-tropism was also detected at 10.3 years post seroconversion, as these variants were derived from new mutations in the V3 loop conferring X4-tropism (data not shown). X4-tropic viruses were also detected at the first time point and dominated throughout infection in participant F3 (red/orange branches) (Fig 6C and S8 Fig). Variant 1 from participant F5 was X4-tropic (red branches), while Variant 2 evolved new X4-tropic viruses that emerged at ~4 years of infection (red/orange branches) (Fig 6E and S8 Fig). *env-gp120* sequences in F8 diverged between 2.2 and 5.3 years post seroconversion, with one lineage evolving X4-variants (yellow/green branches) while a second lineage remained R5-tropic (violet/blue branches) (Fig 6H and S8 Fig).

### Potential N-linked glycosylation sites (PNLGS) changes over time and correlation with disease progression

**Fig 7:**
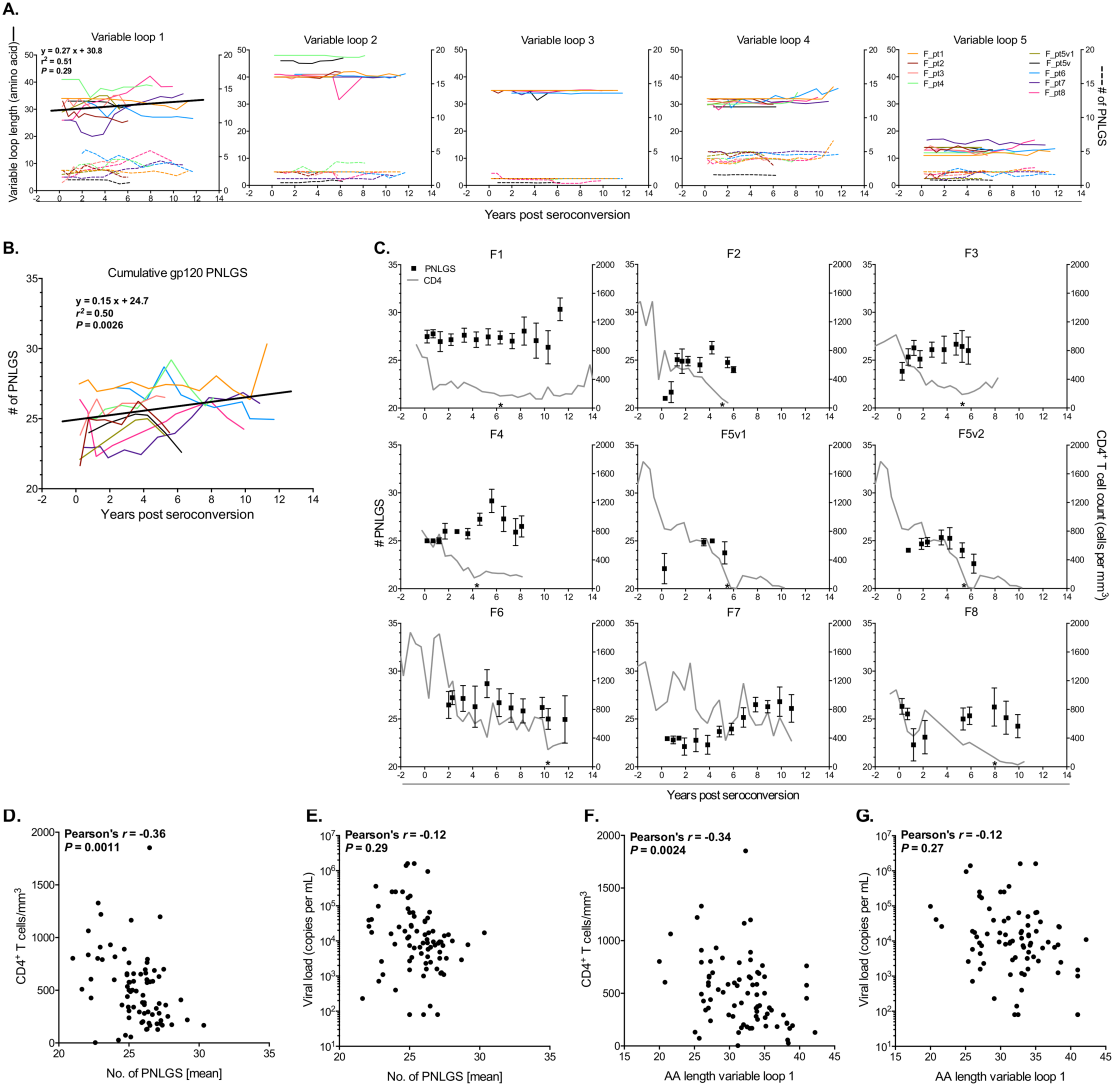
N-linked glycosylation sites and variable loop lengths throughout infection. (**A**) V1-V5 gp120 variable loop lengths (solid line, left y-axis) and number of PNLGS within each variable loop region (dashed line; right y-axis) is shown for each participant. gp120 V1 loop lengths (in amino acids) over time was estimated from a linear mixed-effects model (black line). (**B**) Summary of the cumulative PNLGS over time are shown with a summary (black) line which was estimated from a linear mixed-effects model. (**C**) PNLGS over the entire gp120 protein (filled squares; left y-axis) and CD4^+^ T cell counts (grey line; right y-axis) are shown for each participant. *P*-values derive from a linear regression test to determine if the estimated slope differs from zero. Asterisks (*) indicate when the CD4^+^ T cell count dropped below 200. Average numbers of PNLGS in gp120 at each time point are plotted against CD4^+^ T cell counts (**D**) and plasma viral load (**E**). Amino acid lengths of gp120 V1 were plotted against CD4^+^ T cell counts (**F**) and plasma viral load (**G**). Correlations were analyzed using a Pearson’s correlation test.

HIV-1 PNLGS and variable loop lengths have been reported to decrease upon transmission, although not in subtype B infections [36, 73–76]. In subtype B (primarily men), the frequency of PNLGS in *gp120* tend to increase before declining late in infection, consistent with immune exhaustion [77]. In our cohort, variable loop lengths remained generally static throughout the initial years of infection except for V1, in which loop length increased at a rate of 27% per year (slope did not deviate from zero; *P* = 0.29) (Fig 7A). However, PNLGS increased at an annual rate of 15% (*P* = 0.0026) (solid line in Fig 7B), followed by a late decline in at least 6 of 8 participants (Fig 7C, 7CF2–7CF6 and 7CF8). The mean number of PNLGS per timepoint and length of V1 were negatively associated with CD4+ T cell numbers (Pearson’s *r* = -0.36; *P* = 0.0011 and *r* = -0.34; *P* = 0.0024, respectively) (Fig 7D–7F) but not viral load (Fig 7E–7G). Additionally, the number of PNLGS tracked with viral diversity only within F4 and F7 (Spearman’s ρ = 0.72, *P* = 0.017 and Spearman’s ρ = 0.75, *P* = 0.005, respectively; data not shown).

## Discussion

The present study reports the comprehensive analysis of HIV evolution in women from around the time of seroconversion until the onset of AIDS or ART. All 8 of the women in the WIHS cohort who both seroconverted while on study and were followed for ~6 or more years without ART were studied. These women progressed to AIDS slightly faster than typical progressors, with a median time to CD4^+^ T cell counts below 200 mm^3^/uL of 6.0 years (S13 Fig and Table 1); time to CD4^+^ T cell counts below 200 mm^3^/uL was a median of 7.3 years in the 11 MACS participants studied herein [16]. Median spVL was also similar between the studied WIHS and MACS participants, 0.90x10^4^ and 1.11x10^4^ copies/mL, respectively (Table 1 and [16]). Although spVL was slightly lower in the individuals we studied it was not discernably lower than what is typically found between females and males [1–5]. Thus, the evolutionary rates are consistent between these two cohorts with similar spVL. The incidence of X4 viruses in primary infection, known to be associated with rapid disease progression [78–80], was also high in this female cohort (3/8 = 38% vs ~10% in male cohorts [81–84]). Additionally, the expected inverse correlation between set-point viral load and disease progression (time to AIDS) was not detected in the WIHS cohort (Table 1); we attribute this to cohort size, and thus, future studies are needed to assess the representativeness of this cohort within HIV-infected women.

### Comparison of viral evolutionary metrics

80% of all heterosexual transmission events result in a single founder virus responsible for productive infection [7, 8, 25, 26, 85, 86], yet it remains unknown if a sex bias exists for the acquisition of multiple founders, aside from co-existing sexually transmitted infections and hormonal contraception that have been reported to increase acquisition of multiple variants [7, 87]. We observed little viral heterogeneity early in infection with only one person, F5, showing evidence of multiple variants, in contrast to the acquisition of multiple founder strains within 63% of females reported by Long *et al*. [6]. Demographic and methodological differences between Long *et al*. and our current study could conceivably explain these differences. Long *et al*. analyzed a Kenyan cohort of 42 individuals (32 women and 10 men) infected with HIV-1 subtypes A, C, and D, while our analysis comprised fewer females and all were infected with HIV-1 subtype B. Additionally, the women in our cohort were sampled a median of 91 days from the estimated date of infection while Long *et al*. sampled at a mean of 59 days, thus it may be possible that our study did not detect early virus heterogeneity. However, there was no negative association between viral diversity at the earliest timepoint and time post seroconversion in the current WIHS participants (data not shown). A follow up study by the same group reported that 23% (3/13) of subtype B infected women were infected with heterogeneous virus populations [88]. And, in agreement with our findings, other studies that have documented early viral population diversity in women, including HIV-1 subtype A, C [7, 8, 73, 89–91] and B [92], found them to be near homogeneous. Thus, although the Long *et al*. study sampled more individuals than most previous studies, it remains as an unexplained outlier and we conclude that there are no major differences in early HIV-1 population dynamics between the sexes.

Shankarappa *et al*. [16] reported that for MACS participants (*n* = 9) time to divergence slowdown or stabilization and of peak diversification in *env-C2V5* were temporally related. Divergence stabilization in *env-C2V5* was observed in 50% (n = 4/8) and 73% (*n* = 8/11) of the women and men, respectively, while peak diversity was observed within 75% (*n* = 6/8) of women and 9/11 men. When applied to the entire *env-gp120* region, divergence stabilization was only detected in 2/8 WIHS participants. Viral diversity declined in most of the males [16], whereas a decline phase was found less frequently (4/8) in the females. These differences are due, at least in part, to the availability and uptake of potent ART within the female cohort, not available to the males. Decreases in viral diversity, along with a decrease in variable loop length and loss of N-linked glycosylation sites, may result from reduced selection pressure due to immune dysfunction in latter stages of infection [77, 93, 94].

Previous studies have estimated intra-host HIV-1 nucleotide substitution rates under a variety of assumptions and methodologies, including: linear regression [16, 95, 96], maximum likelihood [97, 98], and Bayesian inference [46, 99]. Using root-to-tip linear regression over the C2V5 region in *env* we found slower evolution in the WIHS vs. the MACS cohorts (0.60% vs. 0.81% mean nucleotide substitutions per site, respectively; *P* = 0.044; data not shown). However, these estimates employed a strict evolutionary clock and when tested, we found that a relaxed clock fit the data better. Furthermore, because this method assumes that sequences are independently sampled without a shared evolutionary history, we chose to instead report evolutionary rate estimates using a Bayesian framework [40]. The Bayesian approach incorporates phylogenetic structure of the sampled sequence data by using MCMC to average individual parameters over a weighted tree topology. And, among the ability to assign priors, it can apply a relaxed molecular clock model [43]. Novitsky *et al*. estimated intra-host evolutionary rates of 32 HIV-1 subtype C infected individuals over a median of 417 days post-seroconversion and reported a median substitution rate of 5.22x10^-3^ (subs./site/year) for *gag* and 1.58x10^-2^ (subs./site/year) for *env-V1C5* [100]. These are very similar to what we found within HIV-1 subtype B infected individuals (6.54x10^-3^ subs./site/year for *gag* and 2.03x10^-2^ subs./site/year for *env-gp120*) despite methodological differences (*i*.*e*., Novitsky collected sequences from only early in infection and like the Shankarappa study, included proviral DNA sequences in their analysis).

A recent report claimed that evolutionary rates of HIV-1 differed by ~30-fold between recently infected individuals and those infected for >1 year, suggesting that HIV-1 diversifies within-hosts in a non-linear manner [17]. However, their conclusion was based on root-to-tip distance-based analysis of *pol* sequences from ART-naïve individuals sampled at either single (*n* = 22) or multiple (*n* = 11; median of 4) timepoints. It is possible that detection of multiple variants within early time points (found in ~5/17 of their acutely infected individuals) skewed the apparent trajectory of their evolutionary slope measures, and transmission and rapid loss of drug resistant variants in *pol* could also affect these estimates. Hence, the results reported in our study are likely to provide a more accurate measure of within-host evolutionary rate estimates. In support of this, a study by Vranken *et al*. also found no evidence for stage-specific evolutionary rates [18]. Our dataset is robust and well sampled over the duration of natural HIV-1 infection. We acknowledge the tradeoff between sampling depth and amplicon size, and chose to produce longer reads to aid in future studies assessing linked or co-evolving sites.

Previous studies have also reported consistent HIV-1 evolution across the genome [100, 101]. Piantadosi *et al*. reported that HIV-1 *gag* and *env* evolutionary rates were highly correlated in a study of 37 females [101]. In that study, however, evolutionary rates were estimated from only two timepoints: during acute and chronic infection. A study by Novitsky *et*. *al*. also reported concordance between *gag* and *env-V1C5* substitution rates in 32 individuals infected with HIV-1 subtype C; however, the average follow up time was only ~400 days (1.3 years) post-seroconversion [100]. That we found uncorrelated evolutionary rates within *gag* and *env-gp120* suggests that experimental design could be responsible for the differences. We argue that extensive longitudinal sampling is superior to estimates based on very limited sample collection when assessing patterns of point mutational viral evolution. We hypothesize that the uncoupled evolution across the genome is most likely due to recombination combined with regional immune-driven escapes or reversions. Recombination of circulating virus with reemerged latent or compartmentalized virus may also uncouple evolutionary rates within genomes. Our findings are similar to Zanini *et al*. who performed extensive whole-genome sequencing in 9 HIV-1-infected individuals over a 5–8 year period [102]. This study reported a 10-fold difference in divergence rates along the genome, and it is evident from their study that regional intra-patient rates are not correlated.

### Evolutionary patterns associated with disease progression

The proportion of individuals in our cohort who developed X4-tropic virus (5/8), despite censored follow up, was not unexpected [16, 78, 80, 103–105]. Similar to previous findings [16], time to peak diversity was correlated with time to peak X4 representation, although no association was found between peak diversity and initial detection of X4 virus in the WIHS, in contrast to the MACS [27]. This distinction might be related to the fact that 3/8 WIHS participants had X4-tropic founder viruses, whereas none of 11 MACS participants did [27]. Other studies have found frequencies of X4/dual-tropic strains between 3.2% and 17.5% in plasma samples from recently infected individuals in the US and Spain [106–108].

Loss of T cell homeostasis, defined by CD3^+^ T cell downward inflection, has been shown to be associated with disease progression and X4-tropic virus dynamics [16, 28, 31, 69–71], however, we found only a non-significant association between CD3^+^ T cell inflection and time to CD4^+^ T cell count < 200 in our study and no association with X4-virus appearance or peak representation in the WIHS. Lastly, changes in patterns of PNLGS have been reported during disease progression [77, 109, 110], and in agreement with prior studies of HIV-1 subtype B infections [77], we observed a generally gradual increase in PNLGS over time followed by a decline in PNLGS, and *d*_*N*_*/d*_*S*_ ratios, during late-stage chronic infection.

### Sex differences in HIV-1 evolution

Recent studies have investigated transmission dynamics and within-host virus evolution to explain risk group-associated (*i*.*e*., MSM, HET, or IDU) differences in HIV-1 evolution at the population level [18, 23, 111], as this may help to inform treatment and prevention strategies within certain demographics. For instance, Vrancken *et al*. observed that HIV evolution was higher in risk groups with a greater proportion of men, comparing between-host rates in MSM and HET [23]. It was posited that HIV may be evolving faster through MSM populations due to increased multivariant transmission [7, 24–26] or to faster within-host evolutionary rates in males. Because the cohorts within our study were not representative of HIV-infected females and males, in terms of spVL differences, we were unable to estimate representative within-host evolutionary rates. Instead, we find equivalent evolutionary rates in men and women having similar spVL, which is expected given the impact of generation time on substitution rates [112].

## Supporting information

S1 Fig Bootstrap phylogenetic analysis of *gag* and *env-gp120* for WIHS participants. RAxML was used to infer best-scoring ML phylograms with bootstrap support values for *gag* and *env-gp120* from all participants. A bootstrap convergence test was performed during 1000 replicate searches. Filled circles represent bootstrap values at the basal node of the clade they support, and are scaled relative to the bootstrap support. Colors correspond to years post seroconversion. The scale at the bottom measures genetic distances in nucleotide substitutions per site.
(PDF)

S2 Fig Inter-participant *gag* phylograms. A Phylogenetic tree of *gag* sequences was inferred for all 8 WIHS participants (see Fig 1 legend for details). External branches corresponding to sequences from the first available timepoint after infection are colored black. Branches in the trees from participants F1, F2, F3, F6, and F7 are shaded light and dark to indicate taxa from early and late infection, respectively, when sequences from early in infection are found at opposite sides of the root node. The scale at the bottom measures genetic distances in nucleotide substitutions per site. Phylograms from each individual were rooted based on outgroup.
(PDF)

S3 Fig Highlighter plots of WIHS and MACS nucleotide alignments. Alignments show nucleotide substitutions relative to the consensus sequence at first timepoint for *gag* and *env-gp120* of the WIHS (A-N) and C2V5 of the MACS (O-Y). Substitutions relative to the first timepoint consensus (master) are color-coded: A = green, C = blue, G = orange, T = red, and grey = gap/deletion. Years post seroconversion is shown to the left of the denoted sequences.
(PDF)

S4 Fig Analysis of variants in participant F5. Unrooted phylograms from participant F5 *env-gp120* (**A**) and gag (**B**) with external node symbols colored according to years post seroconversion. The scale at the bottom of each phylogram shows genetic distances in nucleotide substitutions per site. Highlighter plots for *env-gp120* and *gag*, respectively.
(PDF)

S5 Fig Variant detection in F5_*env-gp120*. (**A**) Phylogenetic tree showing the two variant populations in F5 sequences along with 2,200 randomly chosen subtype B *env-gp120* sequences. The scale at the bottom measures genetic distances in nucleotide substitutions per site. The proportion of *env-gp120* variants detected in plasma of participant F5 found by digital droplet PCR (ddPCR) (**B**) and Sanger sequencing (**C**) are shown. ddPCR was performed using variant specific primers and, therefore did not detect recombinants.
(PDF)

S6 Fig Time to peak diversity in C2V5. (**A**) Average pairwise diversity in C2-V5 was estimated for each timepoint and is shown relative to peak diversification in each participant. (**B**) The association between CD4+ T cell numbers and time of peak C2-V5 diversity. (**C**) The association between the time CD4+ T cells dropped below 200 per mm^3^ and the time of peak C2-V5 diversity. Participants F5 and F7 were not included in this analysis as no observable peak in average pairwise diversity was observed. Associations were assessed using the Spearman’s correlation test. Colored lines represent each of the 8 female participants. Data is put in register (vertical dashed lines) relative to the time of peak average pairwise diversity. (**C**) CD4^+^ T cell counts reaching 200/mm^3^ is plotted relative to time to peak diversity. Time to peak diversity is shown associated with time to predicted X4-tropic genotype detection (**D**) and time to peak X4-tropic genotype representation (**E**). PSSM scores of ~-6 or greater were taken as indicative of X4-tropism). Associations were analyzed using the Spearman’s correlation test; rho and *P*-values are shown. Lines were fit using a least squares linear regression model.
(PDF)

S7 Fig *env-C2V5* and *gag* genetic distance measures over time. Average pairwise nucleotide diversity within timepoints (open circles) and divergence from the founder sequence (defined as the consensus of first timepoint sequences; filled squares) was calculated for *env-C2V5* (**A**) and *gag* (**B**) nucleotide sequences. Mean ± standard error is plotted (error bars are not visible as they were not as large as the data points). The proportion of predicted X4-tropic strains (magenta circles) computed by the PSSM Subtype B scoring algorithm is shown at each timepoint. The two distinct variants in C2V5 within participant F5 were analyzed separately. HIV viral RNA load (copies per mL; red lines), CD4^+^ and CD8^+^ T cell counts (cells per mm^3^; blue and green lines, respectively), and visits with ART administration (black asterisks (*) at the bottom of each panel) are shown. The arrow at the bottom of each panel indicates the first time at which CD4^+^ T cell counts fell below 200. Dashed vertical lines indicate the time of peak viral diversity, when detected, and the solid vertical lines (F6, panel A and F3, panel B) indicates the time at which divergence from the founder strain stabilized or decreased.
(PDF)

S8 Fig CD4^+^ T cell tropism predicted from individual V3-loop sequences. (**A**) PSSM scores to predict HIV-1 co-receptor tropism (see Methods) were estimated for all 8 participants (left y-axis, open maroon circles. Plots show CD4^+^ and CD8^+^ T cell counts (right y-axis; blue and green lines, respectively), HIV viral RNA load (right y-axis; red line), and years post seroconversion (x-axis). PSSM scores were plotted on a continuous +10 to -15 scale. Higher scores indicate CXCR4 co-receptor usage, while lower scores indicate CCR5 co-receptor usage. Values above ~-6 reliably indicate X4 tropism. The arrow at the bottom of each panel indicates the first time at which CD4^+^ T cell counts fell below 200.
(PDF)

S9 Fig CD3^+^ T cell inflection points (IPs). (**A-H**) CD3+ T cell IPs were estimated for all participants using a segmented linear regression with a constrained initial slope of 0 (see Methods). Colored lines indicate the log-transformed number of CD3+ T cells. Solid black lines indicate the estimated segmented linear regression line and estimated IPs are shown as the midpoint of the two dates surrounding the IP (indicated by arrow). An estimated IP required at least three measures before and after a potential midpoint. Time to CD3^+^ T cell inflection is shown relative to time to (**I**) peak viral diversity in *env-gp120*, (**J**) CD4^+^ T cell count below 200, (**K**) predicted X4-tropic genotype, and (**L**) peak X4-tropic genotype representation. Associations were analyzed using the Spearman’s correlation test; rho and *P*-values are shown. Lines were fit using a least squares linear regression model.
(PDF)

S10 Fig Location of positively selected sites in *gag* and *env*. A mixed effects model of evolution was used to infer codons undergoing diversifying positive selection for *gag* (A), *env-gp120* (B), and C2V5 (C) sequences within WIHS and (D) MACS participants. Participant identifiers are displayed on the y-axis with codon positions set to the HXB2 subtype B reference sequence displayed on the x-axis. *Gag* and *env-gp120* coding regions are displayed atop each panel. Vertical lines are shown for all sites inferred to be experiencing positive selection with a *P*-value < 0.05. Lines are colored from red to black and indicate a false discovery rate q-value (from 0 to 0.5) of each site’s associated *P*-value.
(PDF)

S11 Fig Estimations of synonymous (*d*_*S*_) and nonsynonymous (*d*_*N*_) divergence rates and *d*_*N*_/*d*_*S*_ ratios. Accumulation of *d*_*S*_ and *d*_*N*_ substitutions per site are shown for *gag* (**A**) and *env-gp120* (**B**). Light blue and yellow lines, respectively, correspond to *d*_*S*_ values and violet and orange lines, respectively, correspond to *d*_*N*_. Solid gray lines show RNA viral load and dotted gray lines show CD4^+^ T cell counts. Summary of *d*_*S*_ (**C**) and *d*_*N*_ (**D**) for *gag* and *env-gp120* in all 8 participants, including estimated mean group rates (solid black lines). Estimations of *d*_*N*_/*d*_*S*_ ratios are shown over time for C2V5 in WIHS (**E**) and MACS (**F**) participants. Average pairwise substitution rates were determined from comparisons to founder strains using PAML. A linear mixed-effects model was used to compare substitution rate differences between viral genes. Mean rate ± SEM are shown for each rate estimate.
(PDF)

S12 Fig Phylogenetic analysis of *gag* nucleotide sequences. (**A-H**) Maximum likelihood *gag* phylogenetic trees of sequence from each participant were reconstructed using PhyML v3.0 (see Methods) and rooted to earliest timepoint sequences. Tip symbols show years post seroconversion (colored circles). The scale at the bottom measures genetic distances in nucleotide substitutions per site.
(PDF)

S13 Fig Summary of clinical measures. Summary plots of (**A**) Set-point viral load, (**B**) time to CD4^+^ T cells < 200 counts/mm^3^, (**C**) viral load, and (**D**) CD4^+^ T cell numbers are shown for each participant. N/A, not applicable because participant F7 did not reach CD4^+^ T cell count < 200.
(PDF)

S1 Table Comparison of nucleotide substitution rate estimates for constant and exponential growth population coalescent models. (DOCX)

S2 Table HIV *gag* and *env-gp120* sequences collected for analysis. (DOCX)

S3 Table Rate estimations using a Bayesian phylogenetic approach. (DOCX)
