# Supplementary material for: Patterns and rates of viral evolution in HIV-1 subtype B infected females and males
Source: PLoS One. 2017 Oct 18;12(10):e0182443. doi: 10.1371/journal.pone.0182443 (PMC5646779; doi:10.1371/journal.pone.0182443)

Figure S1

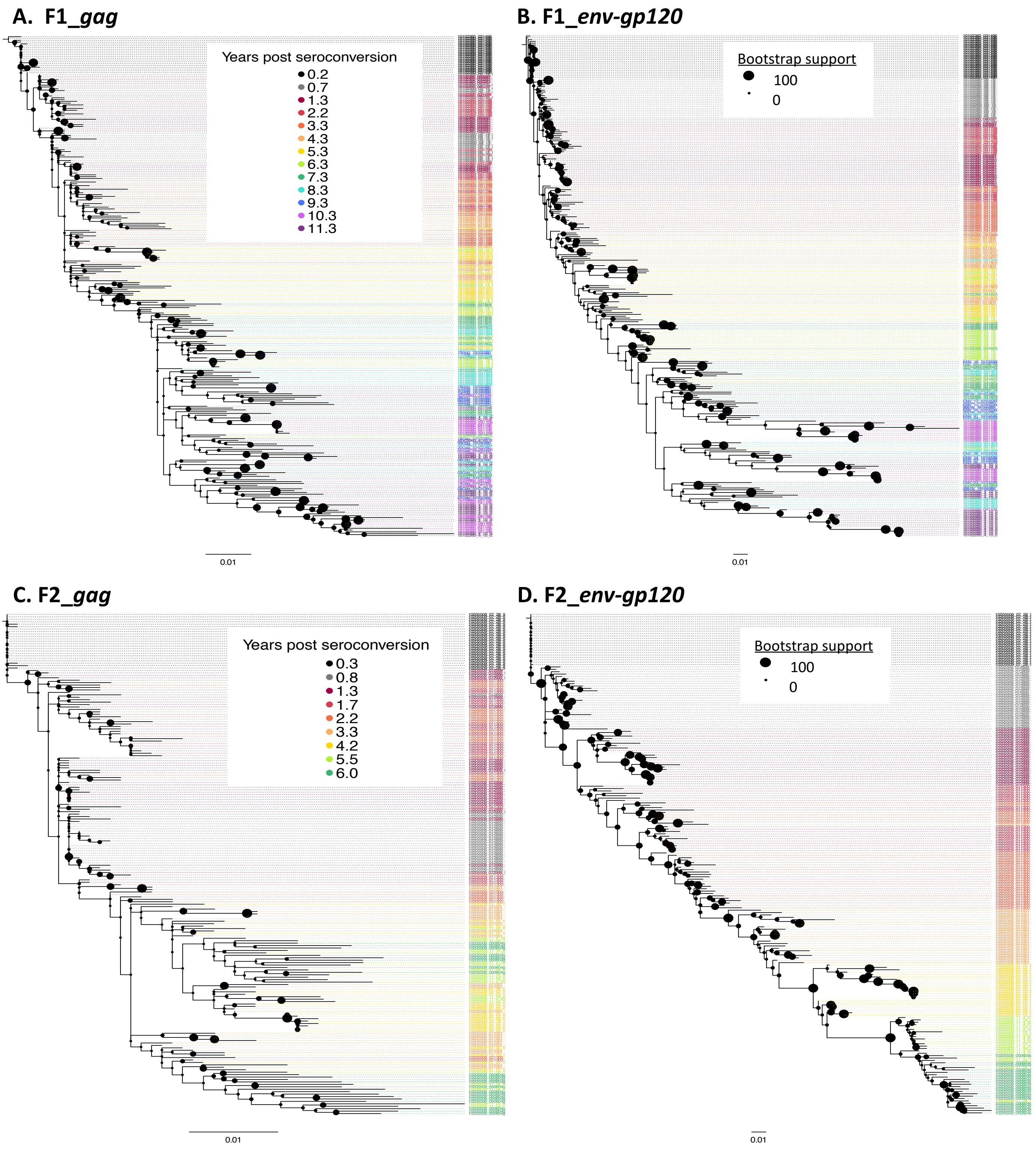
A. F1_gag
Years post seroconversion
0.2
0.7
1.3
2.2
3.3
4.3
5.3
6.3
7.3
8.3
9.3
10.3
11.3
0.01
B. F1_env-gp120
Bootstrap support
100
0
0.01
C. F2_gag
Years post seroconversion
0.3
0.8
1.3
1.7
2.2
3.3
4.2
5.5
6.0
0.01
D. F2_env-gp120
Bootstrap support
100
0
0.01

Figure S1 (con't)

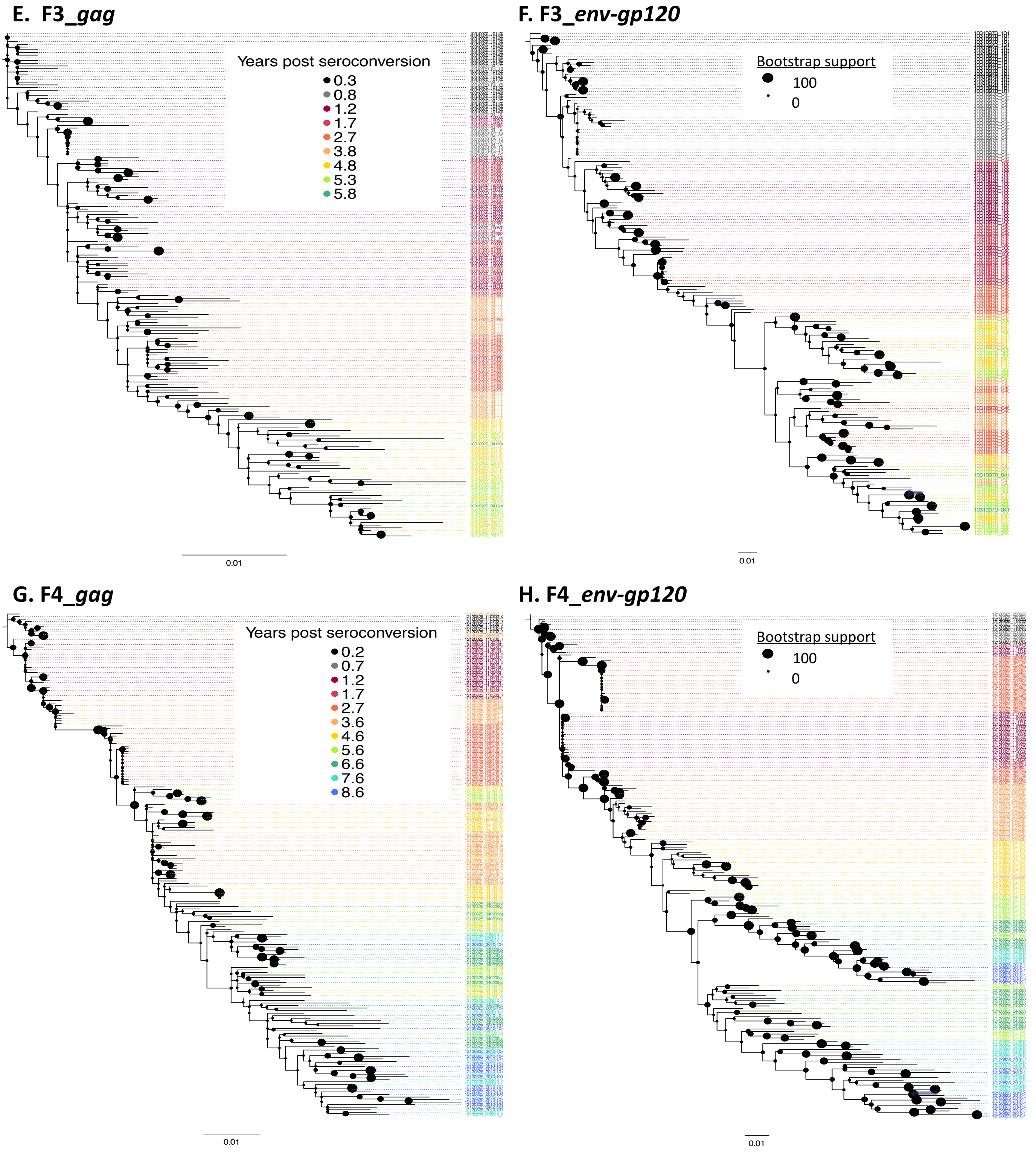

Figure S1 (con't)

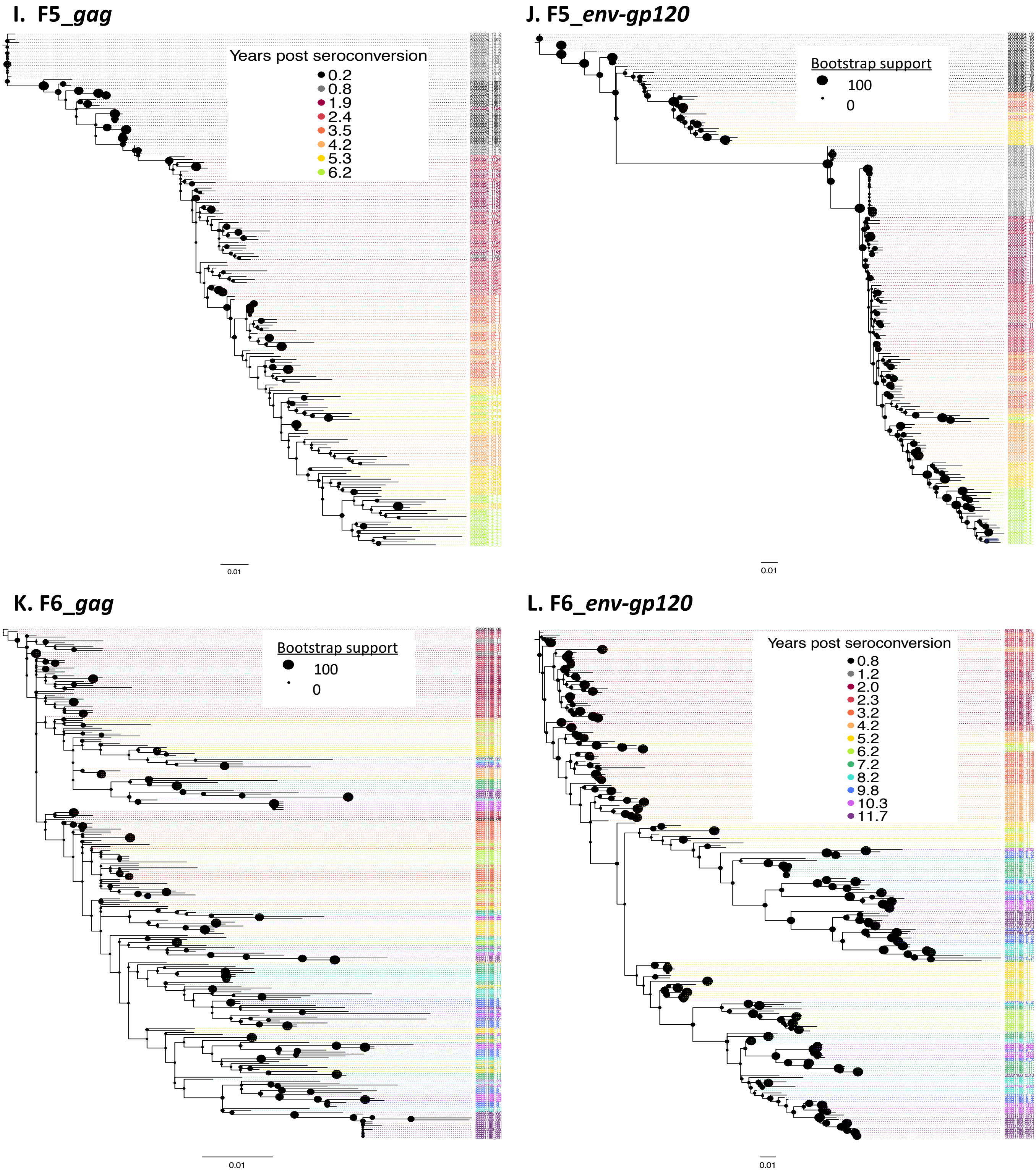

Figure S1 (con't)

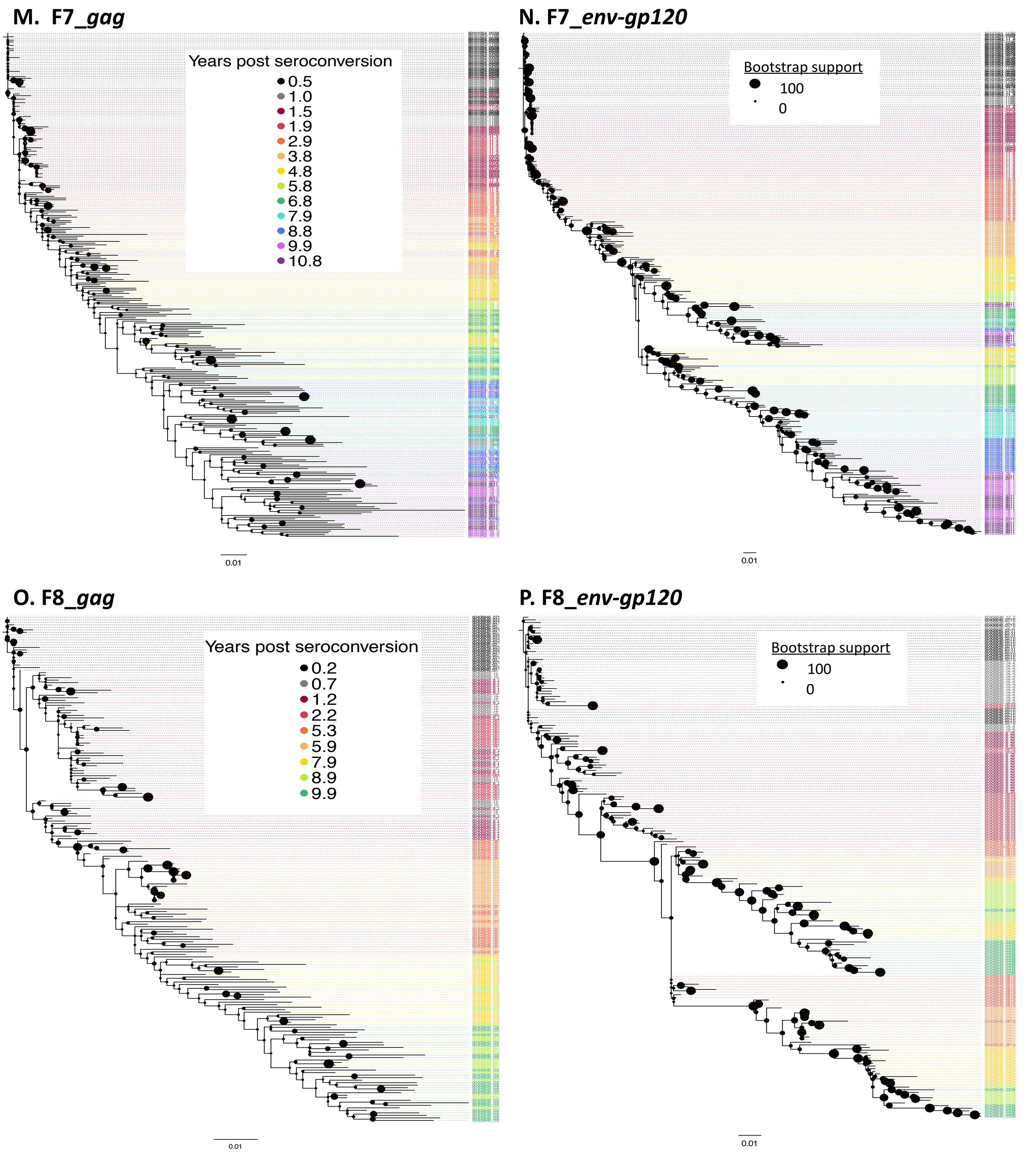

Supplement: S1 Fig — RAxML was used to infer best-scoring ML phylograms with bootstrap support values for gag and env-gp120 from all participants. A bootstrap convergence test was performed during 1000 replicate searches. Filled circles represent bootstrap values at the basal node of the clade they support, and are scaled relative to the bootstrap support. Colors correspond to years post seroconversion. The scale at the bottom measures genetic distances in nucleotide substitutions per site. (PDF) [file pone.0182443.s001.pdf]
