# Supplementary figures and images for: Patterns and rates of viral evolution in HIV-1 subtype B infected females and males
Source: PLoS One. 2017 Oct 18;12(10):e0182443. doi: 10.1371/journal.pone.0182443 (PMC5646779; doi:10.1371/journal.pone.0182443)

Figure S2

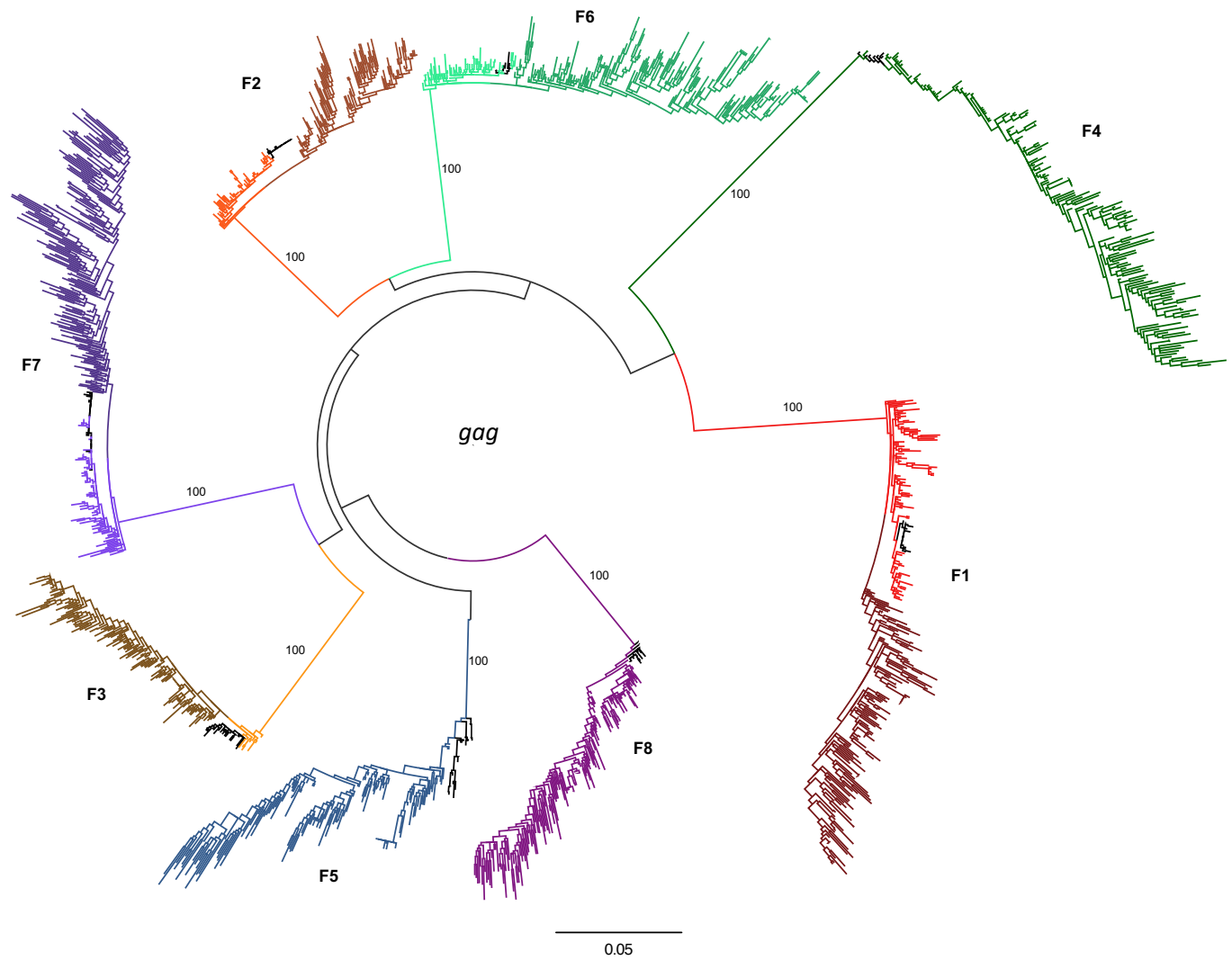

Supplement: S2 Fig — A Phylogenetic tree of gag sequences was inferred for all 8 WIHS participants (see Fig 1 legend for details). External branches corresponding to sequences from the first available timepoint after infection are colored black. Branches in the trees from participants F1, F2, F3, F6, and F7 are shaded light and dark to indicate taxa from early and late infection, respectively, when sequences from early in infection are found at opposite sides of the root node. The scale at the bottom measures genetic distances in nucleotide substitutions per site. Phylograms from each individual were rooted based on outgroup. (PDF) [file pone.0182443.s002.pdf]

Figure S4

A.

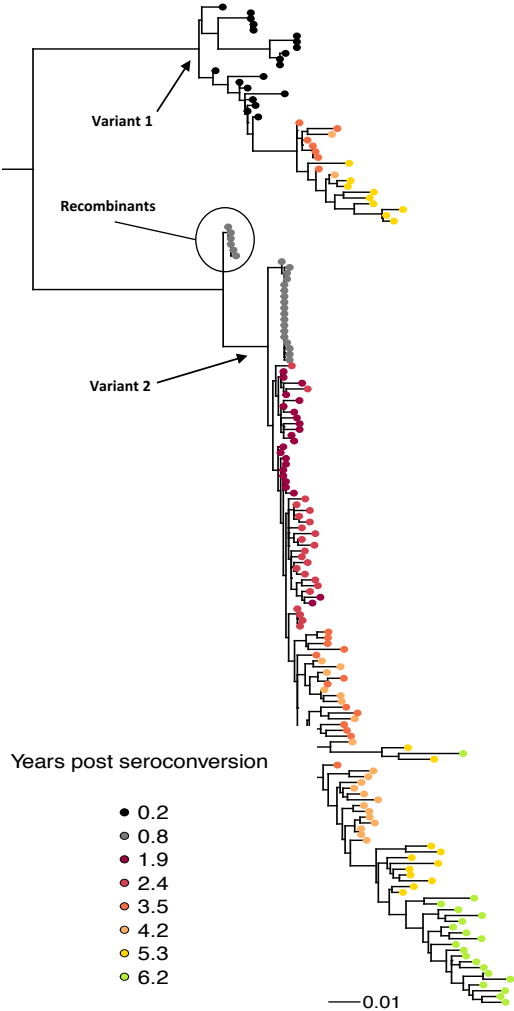

B.

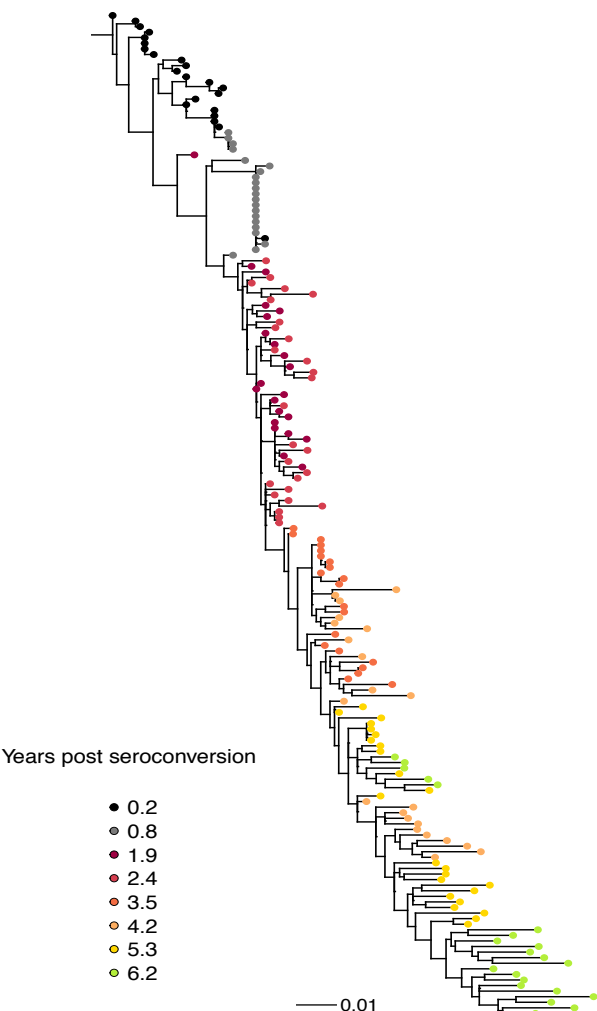

Supplement: S4 Fig — Unrooted phylograms from participant F5 env-gp120 (A) and gag (B) with external node symbols colored according to years post seroconversion. The scale at the bottom of each phylogram shows genetic distances in nucleotide substitutions per site. Highlighter plots for env-gp120 and gag, respectively. (PDF) [file pone.0182443.s004.pdf]

Figure S5

A.

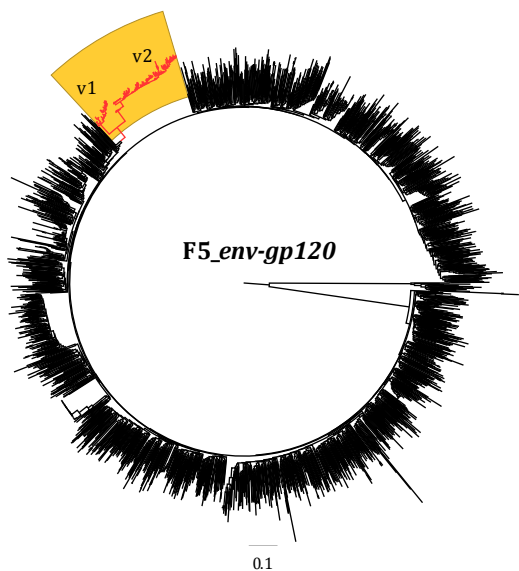

B.

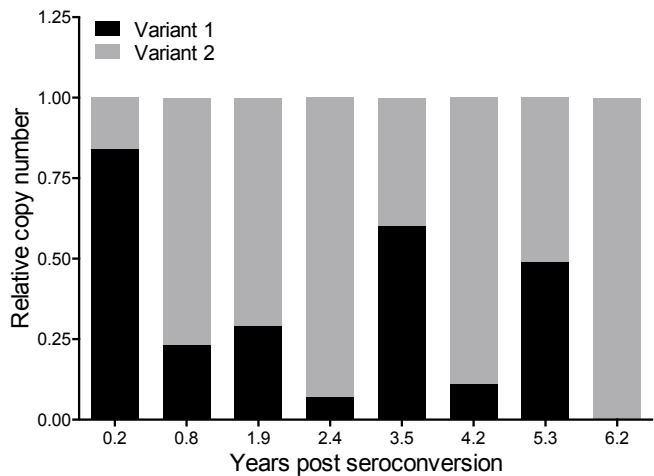

C.

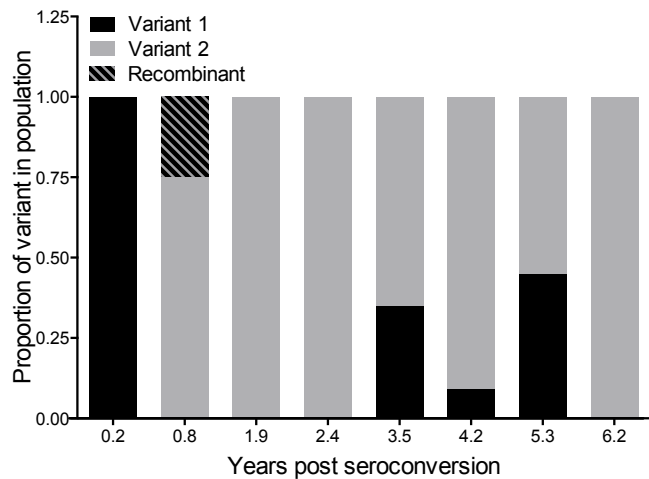

Supplement: S5 Fig — (A) Phylogenetic tree showing the two variant populations in F5 sequences along with 2,200 randomly chosen subtype B env-gp120 sequences. The scale at the bottom measures genetic distances in nucleotide substitutions per site. The proportion of env-gp120 variants detected in plasma of participant F5 found by digital droplet PCR (ddPCR) (B) and Sanger sequencing (C) are shown. ddPCR was performed using variant specific primers and, therefore did not detect recombinants. (PDF) [file pone.0182443.s005.pdf]

Figure S6

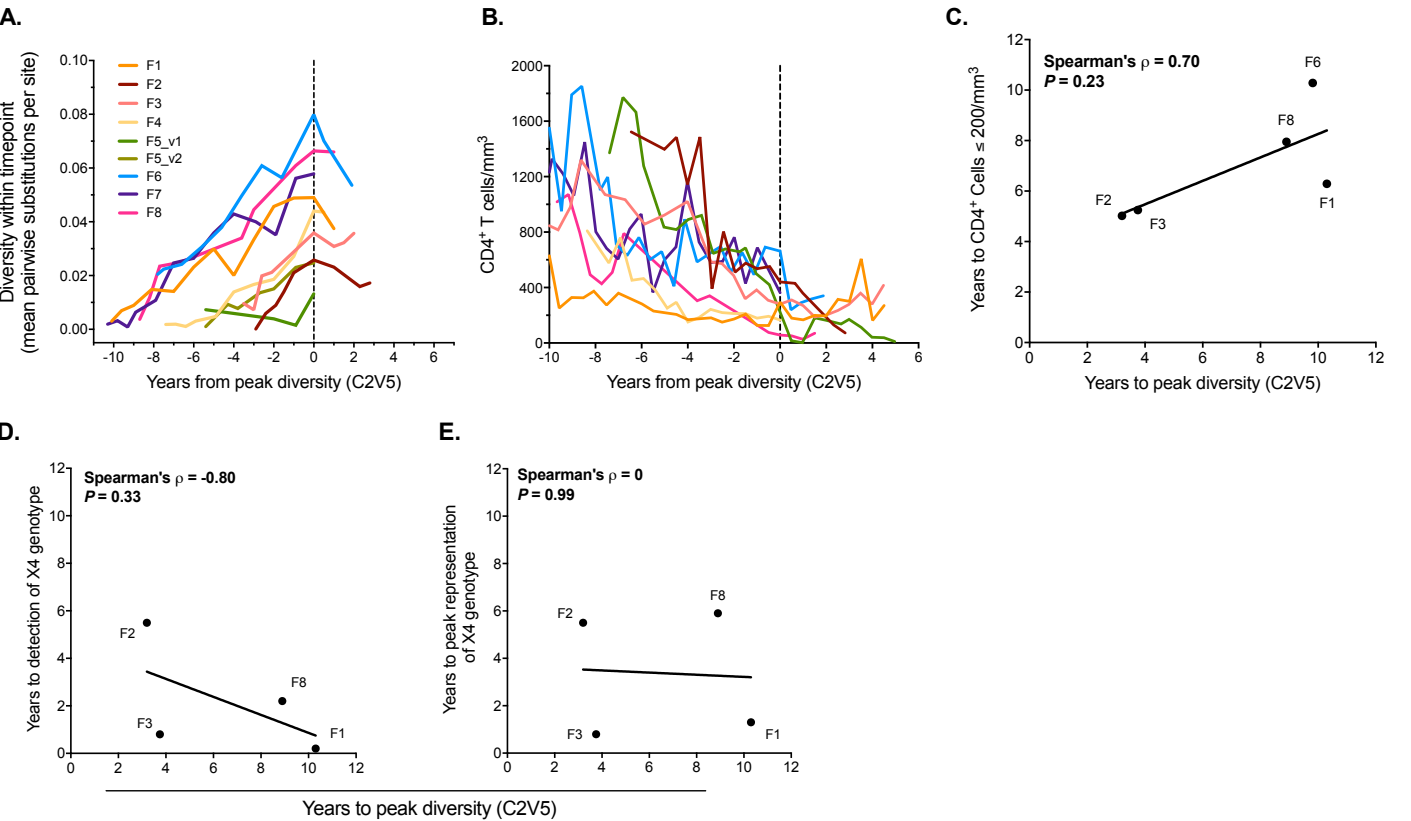

Supplement: S6 Fig — (A) Average pairwise diversity in C2-V5 was estimated for each timepoint and is shown relative to peak diversification in each participant. (B) The association between CD4+ T cell numbers and time of peak C2-V5 diversity. (C) The association between the time CD4+ T cells dropped below 200 per mm3 and the time of peak C2-V5 diversity. Participants F5 and F7 were not included in this analysis as no observable peak in average pairwise diversity was observed. Associations were assessed using the Spearman’s correlation test. Colored lines represent each of the 8 female participants. Data is put in register (vertical dashed lines) relative to the time of peak average pairwise diversity. (C) CD4+ T cell counts reaching 200/mm3 is plotted relative to time to peak diversity. Time to peak diversity is shown associated with time to predicted X4-tropic genotype detection (D) and time to peak X4-tropic genotype representation (E). PSSM scores of ~-6 or greater were taken as indicative of X4-tropism). Associations were analyzed using the Spearman’s correlation test; rho and P-values are shown. Lines were fit using a least squares linear regression model. (PDF) [file pone.0182443.s006.pdf]

Figure S7

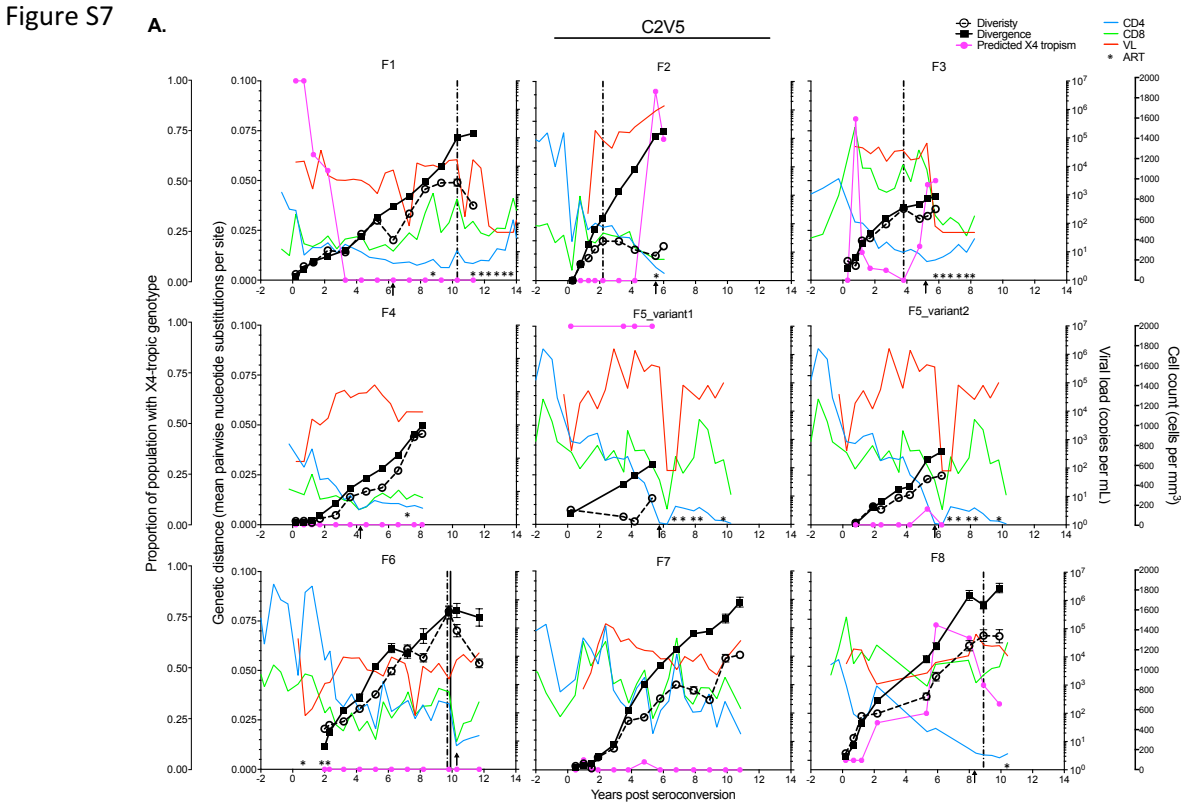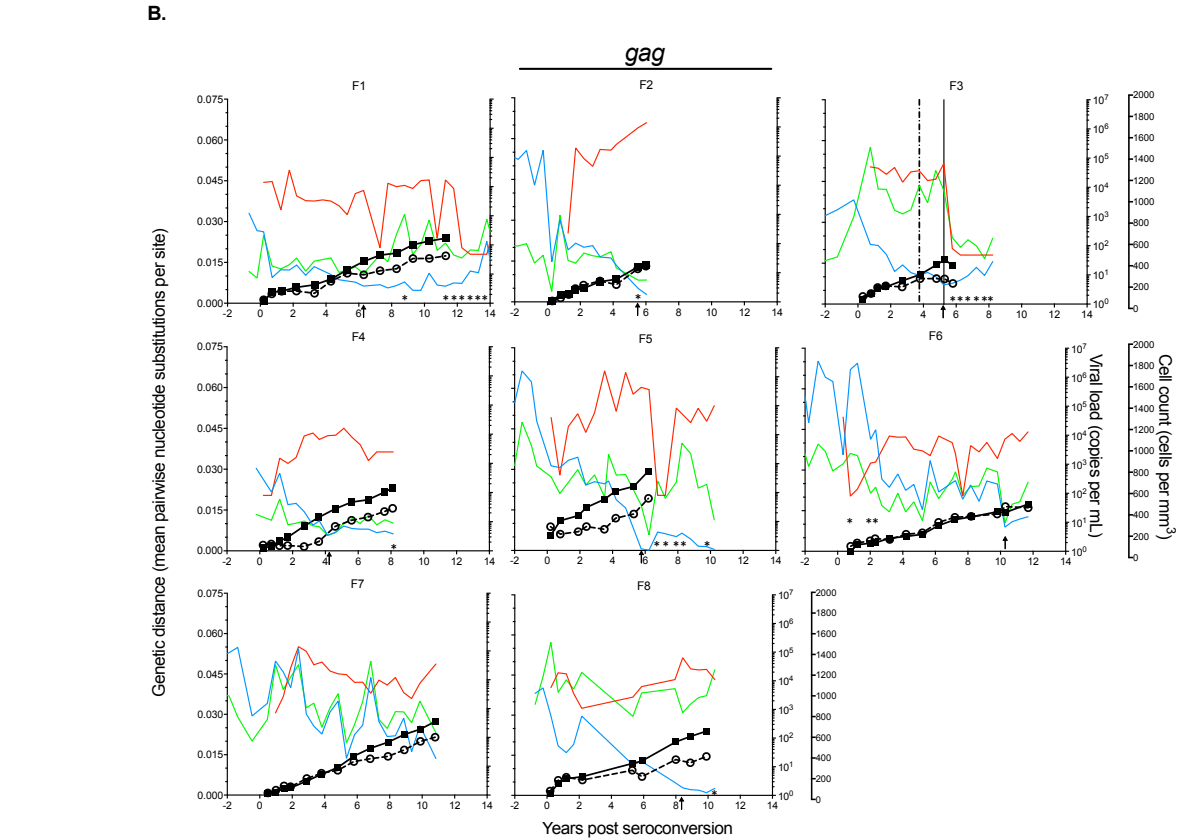

Supplement: S7 Fig — Average pairwise nucleotide diversity within timepoints (open circles) and divergence from the founder sequence (defined as the consensus of first timepoint sequences; filled squares) was calculated for env-C2V5 (A) and gag (B) nucleotide sequences. Mean ± standard error is plotted (error bars are not visible as they were not as large as the data points). The proportion of predicted X4-tropic strains (magenta circles) computed by the PSSM Subtype B scoring algorithm is shown at each timepoint. The two distinct variants in C2V5 within participant F5 were analyzed separately. HIV viral RNA load (copies per mL; red lines), CD4+ and CD8+ T cell counts (cells per mm3; blue and green lines, respectively), and visits with ART administration (black asterisks (*) at the bottom of each panel) are shown. The arrow at the bottom of each panel indicates the first time at which CD4+ T cell counts fell below 200. Dashed vertical lines indicate the time of peak viral diversity, when detected, and the solid vertical lines (F6, panel A and F3, panel B) indicates the time at which divergence from the founder strain stabilized or decreased. (PDF) [file pone.0182443.s007.pdf]

Figure S8

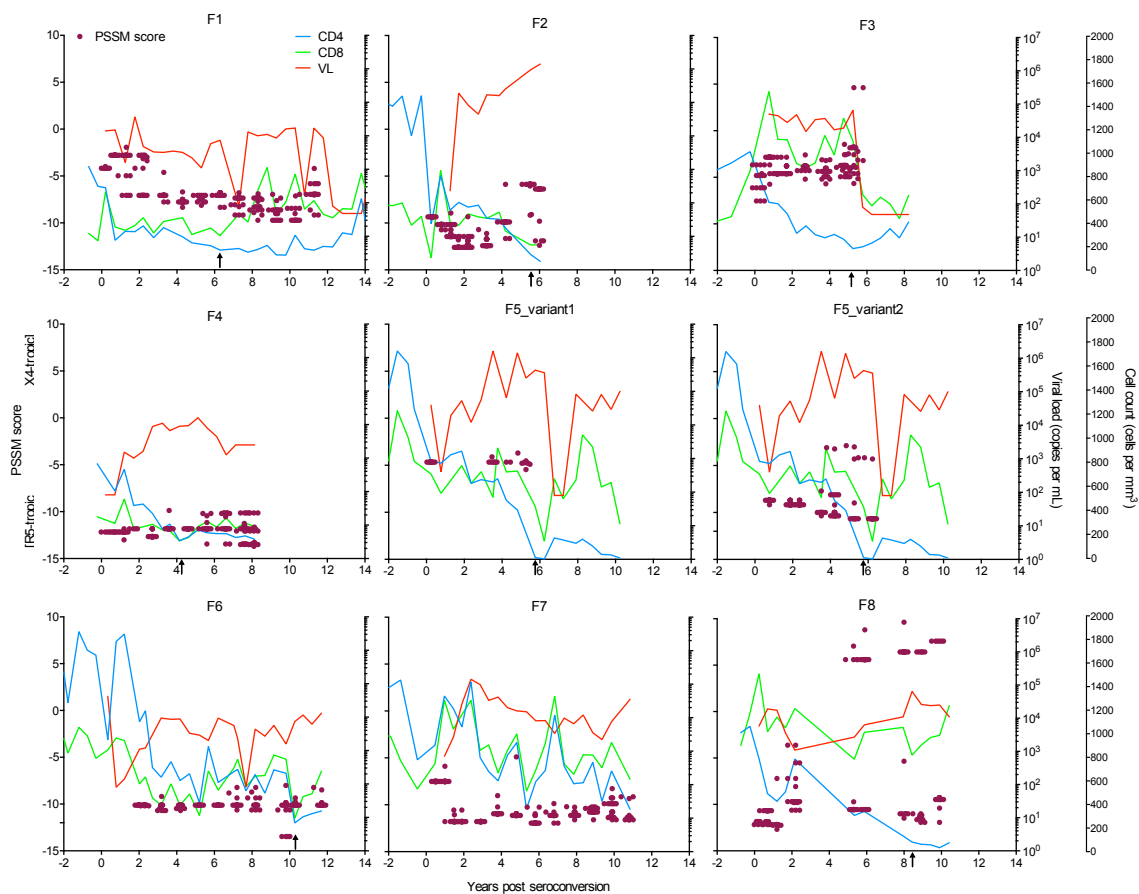

Supplement: S8 Fig — (A) PSSM scores to predict HIV-1 co-receptor tropism (see Methods) were estimated for all 8 participants (left y-axis, open maroon circles. Plots show CD4+ and CD8+ T cell counts (right y-axis; blue and green lines, respectively), HIV viral RNA load (right y-axis; red line), and years post seroconversion (x-axis). PSSM scores were plotted on a continuous +10 to -15 scale. Higher scores indicate CXCR4 co-receptor usage, while lower scores indicate CCR5 co-receptor usage. Values above ~-6 reliably indicate X4 tropism. The arrow at the bottom of each panel indicates the first time at which CD4+ T cell counts fell below 200. (PDF) [file pone.0182443.s008.pdf]

Figure S9

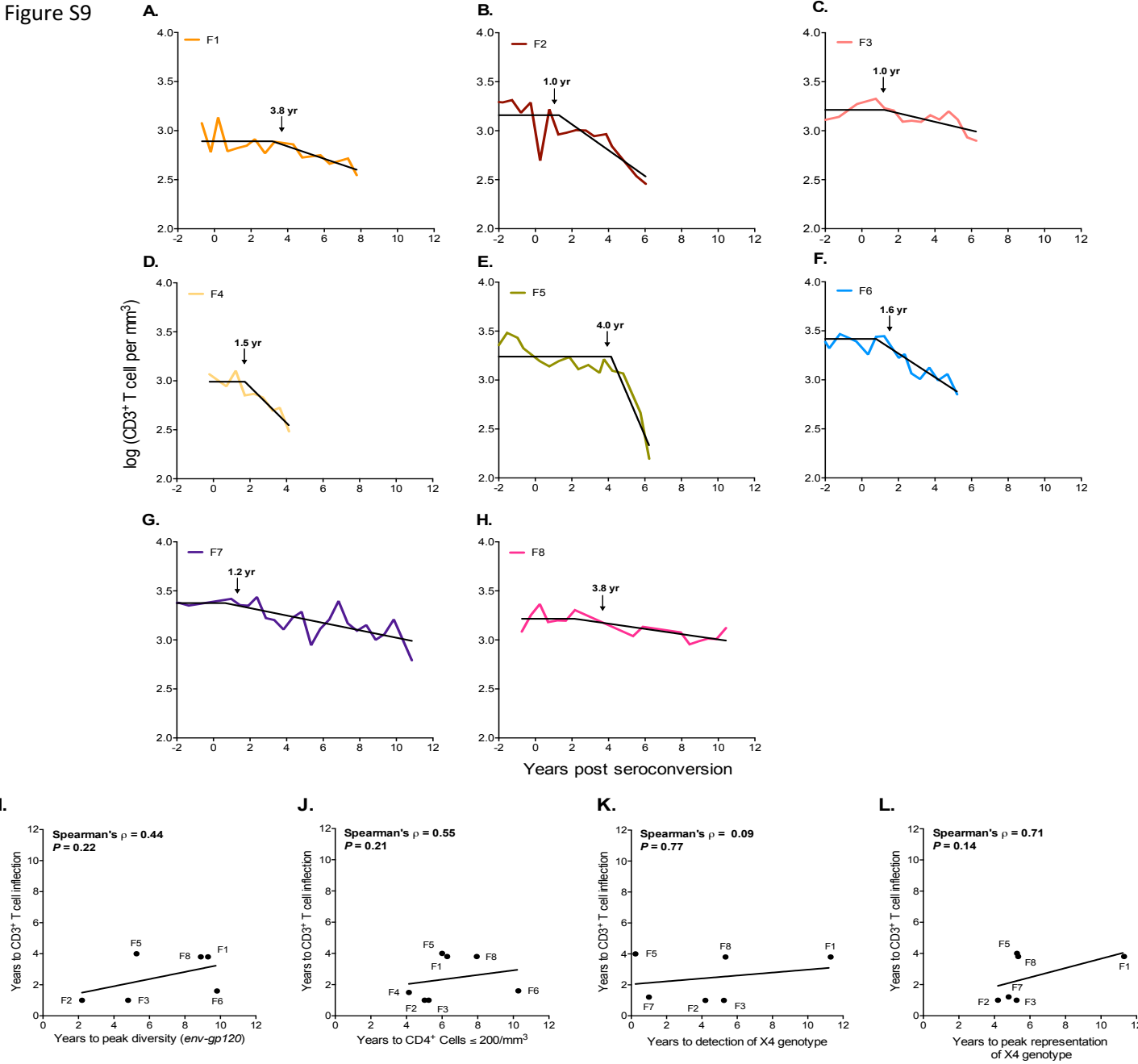

Supplement: S9 Fig — (A-H) CD3+ T cell IPs were estimated for all participants using a segmented linear regression with a constrained initial slope of 0 (see Methods). Colored lines indicate the log-transformed number of CD3+ T cells. Solid black lines indicate the estimated segmented linear regression line and estimated IPs are shown as the midpoint of the two dates surrounding the IP (indicated by arrow). An estimated IP required at least three measures before and after a potential midpoint. Time to CD3+ T cell inflection is shown relative to time to (I) peak viral diversity in env-gp120, (J) CD4+ T cell count below 200, (K) predicted X4-tropic genotype, and (L) peak X4-tropic genotype representation. Associations were analyzed using the Spearman’s correlation test; rho and P-values are shown. Lines were fit using a least squares linear regression model. (PDF) [file pone.0182443.s009.pdf]

Figure S10

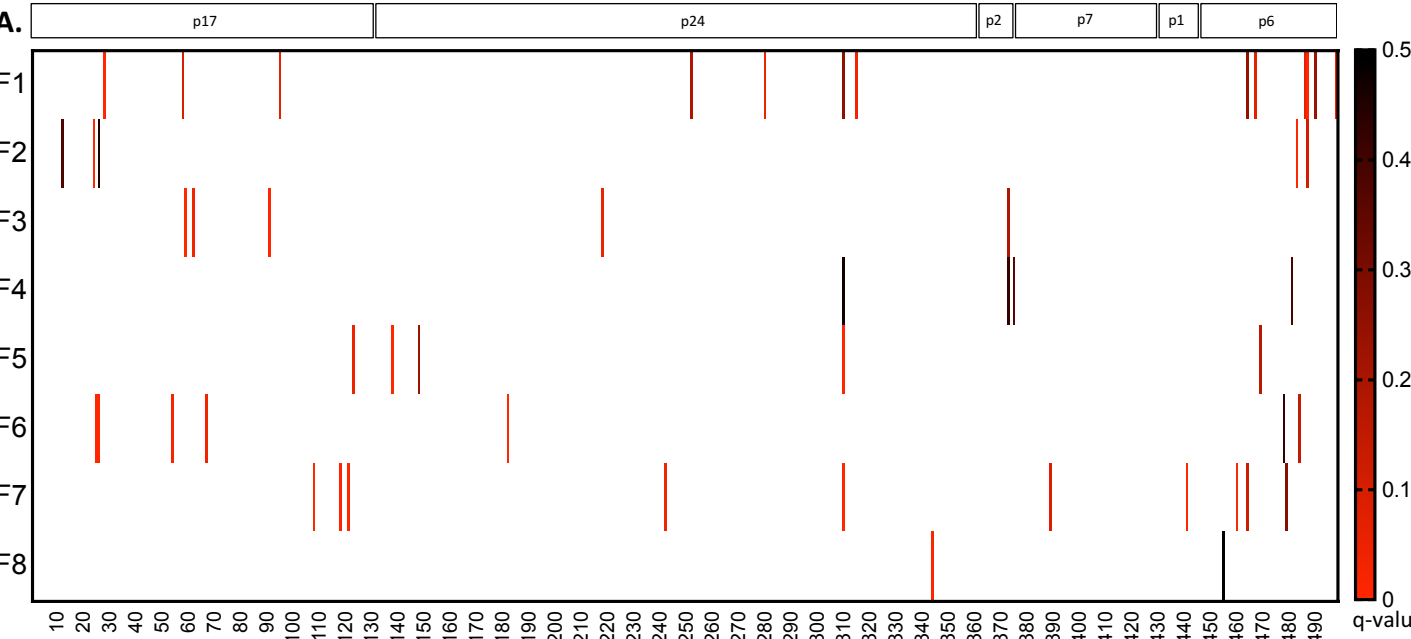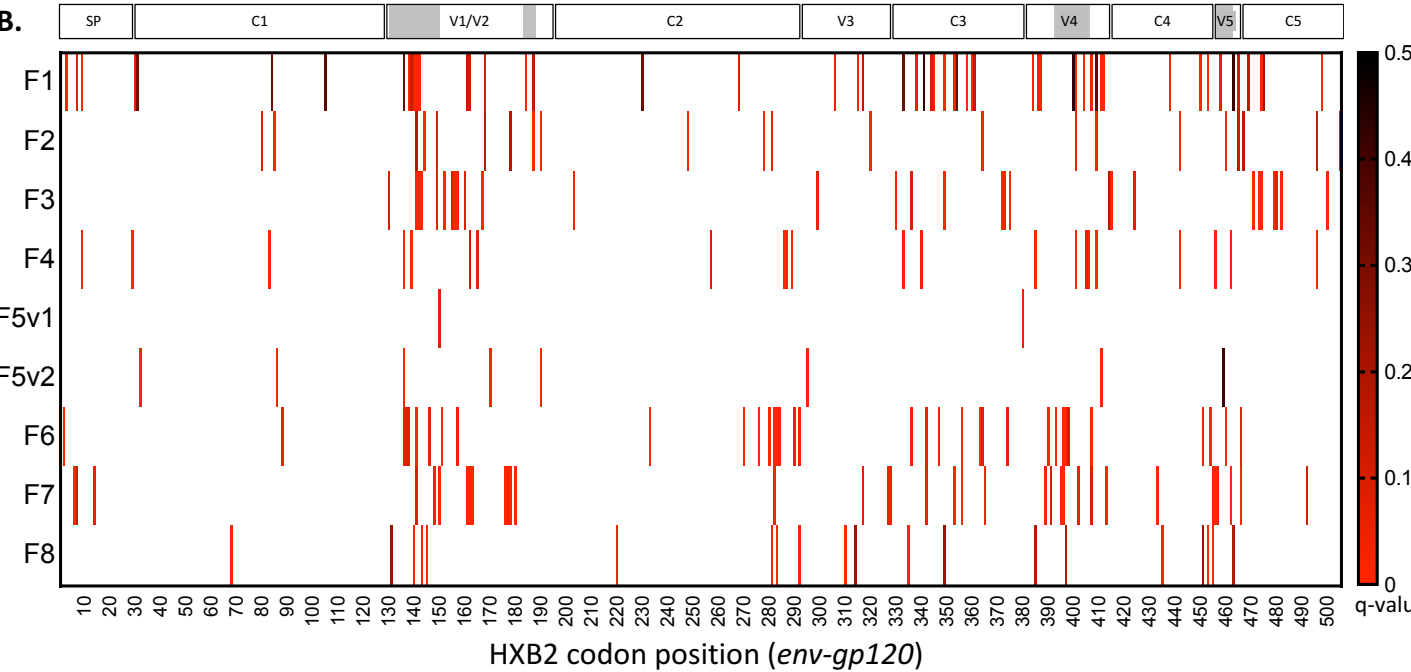

Figure S10 (con't)

C.

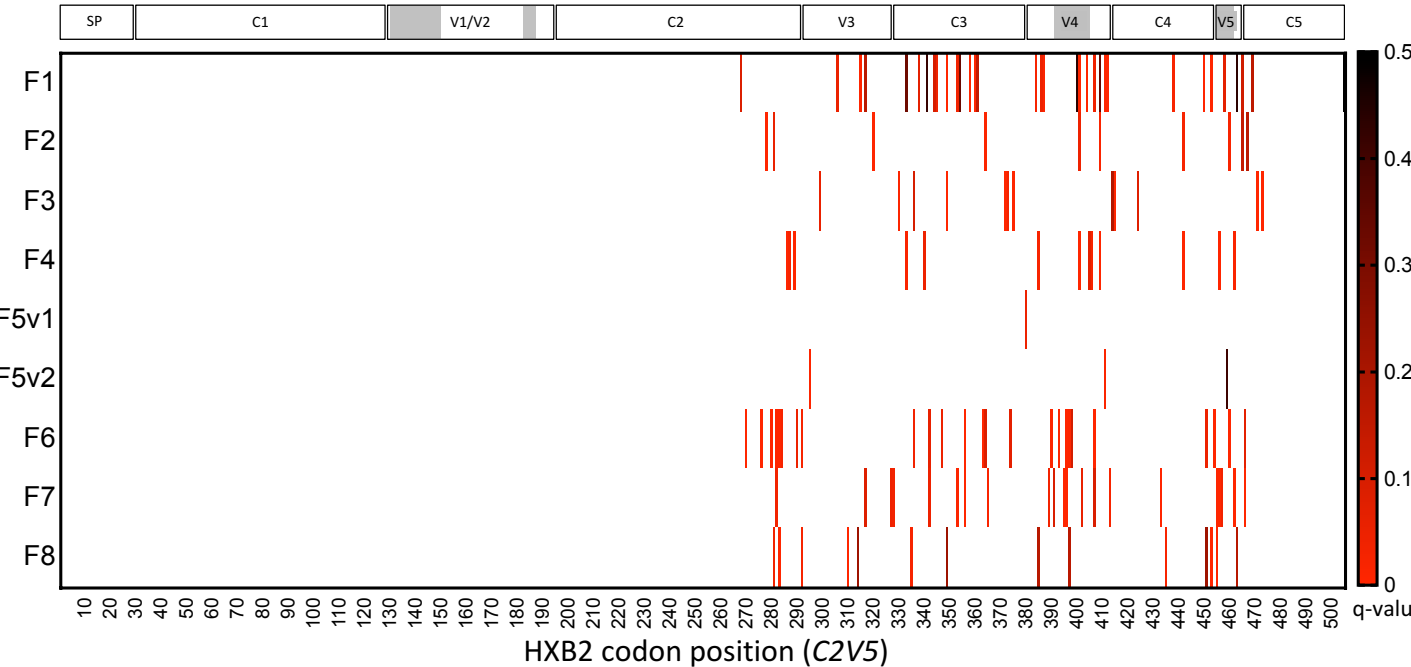

D.

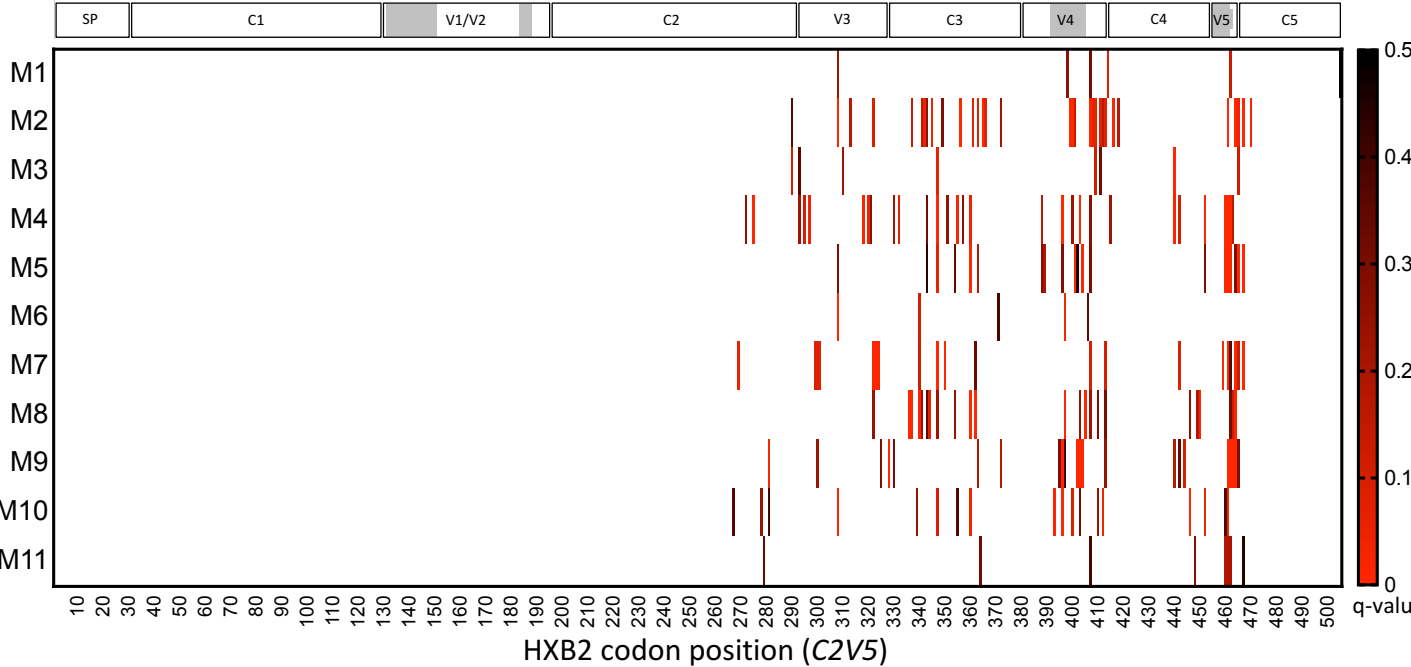

Supplement: S10 Fig — A mixed effects model of evolution was used to infer codons undergoing diversifying positive selection for gag (A), env-gp120 (B), and C2V5 (C) sequences within WIHS and (D) MACS participants. Participant identifiers are displayed on the y-axis with codon positions set to the HXB2 subtype B reference sequence displayed on the x-axis. Gag and env-gp120 coding regions are displayed atop each panel. Vertical lines are shown for all sites inferred to be experiencing positive selection with a P-value < 0.05. Lines are colored from red to black and indicate a false discovery rate q-value (from 0 to 0.5) of each site’s associated P-value. (PDF) [file pone.0182443.s010.pdf]

Figure S11 (con't)

C.

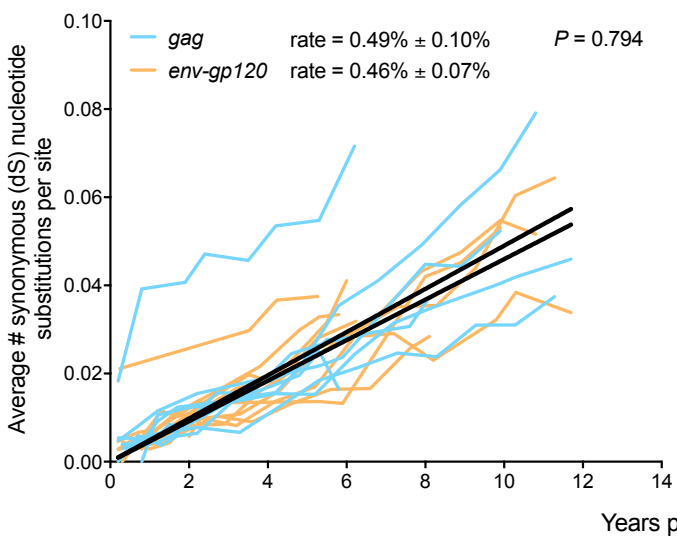

D.

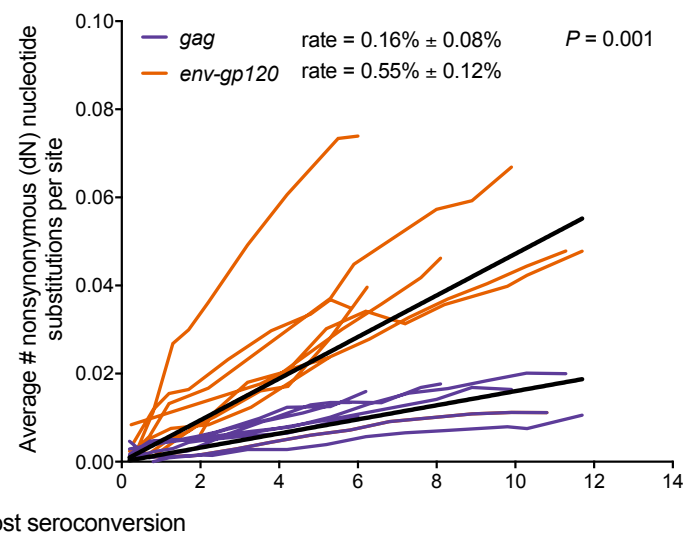

Figure S11 (con't)

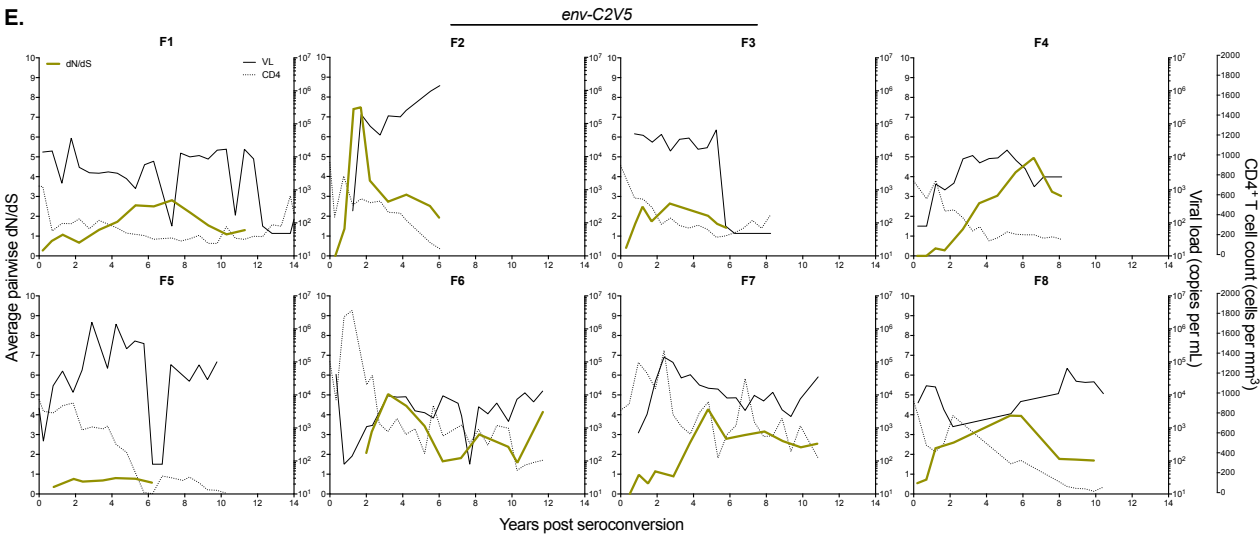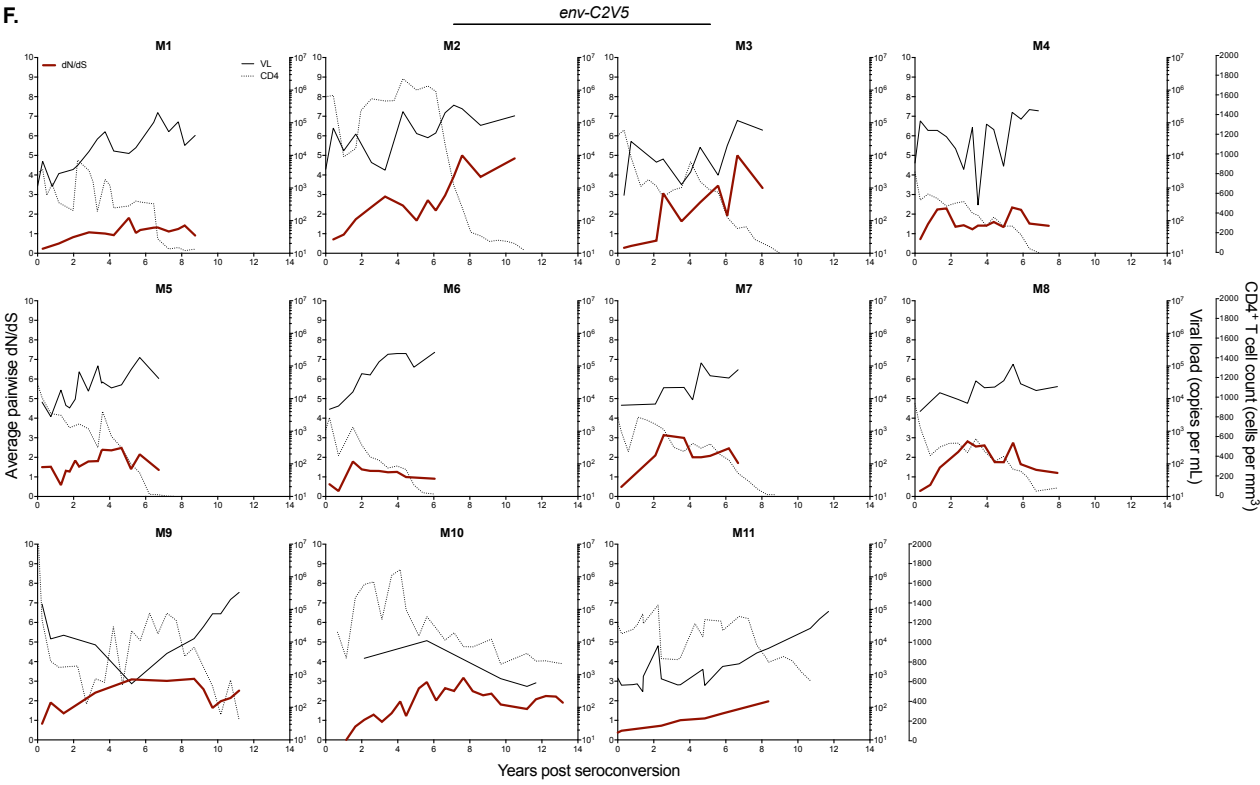

Supplement: S11 Fig — Accumulation of dS and dN substitutions per site are shown for gag (A) and env-gp120 (B). Light blue and yellow lines, respectively, correspond to dS values and violet and orange lines, respectively, correspond to dN. Solid gray lines show RNA viral load and dotted gray lines show CD4+ T cell counts. Summary of dS (C) and dN (D) for gag and env-gp120 in all 8 participants, including estimated mean group rates (solid black lines). Estimations of dN/dS ratios are shown over time for C2V5 in WIHS (E) and MACS (F) participants. Average pairwise substitution rates were determined from comparisons to founder strains using PAML. A linear mixed-effects model was used to compare substitution rate differences between viral genes. Mean rate ± SEM are shown for each rate estimate. (PDF) [file pone.0182443.s011.pdf]

Figure S12

**A. F1\_gag**

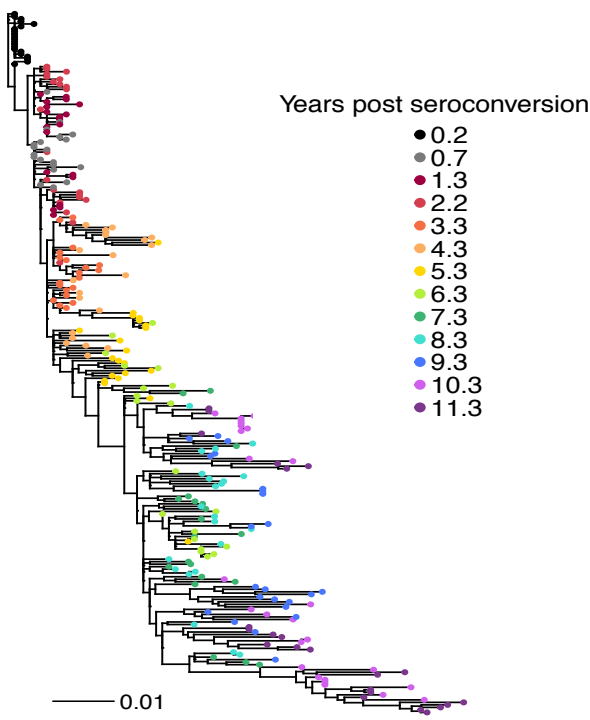

**B. F2\_gag**

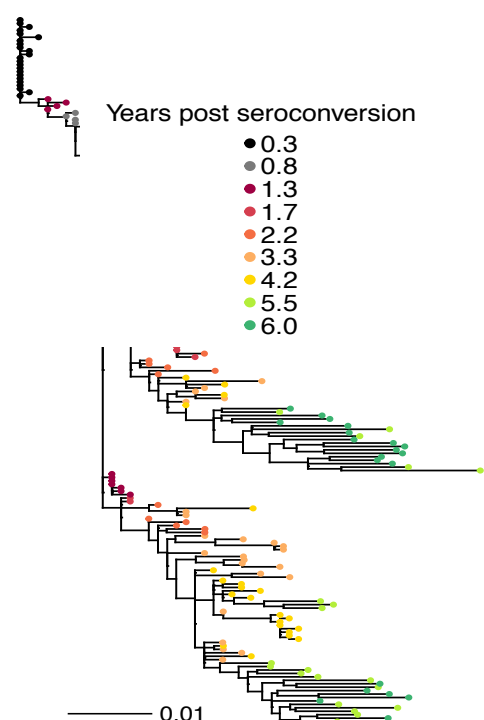

**C. F3\_gag**

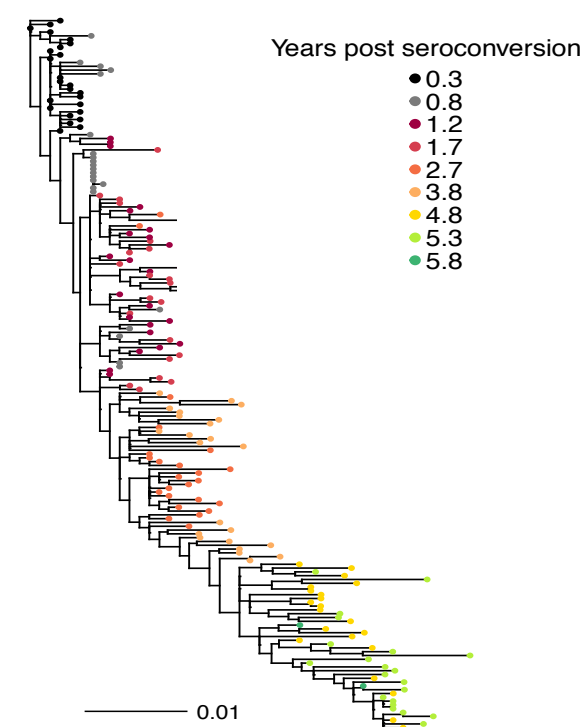

**D. F4\_gag**

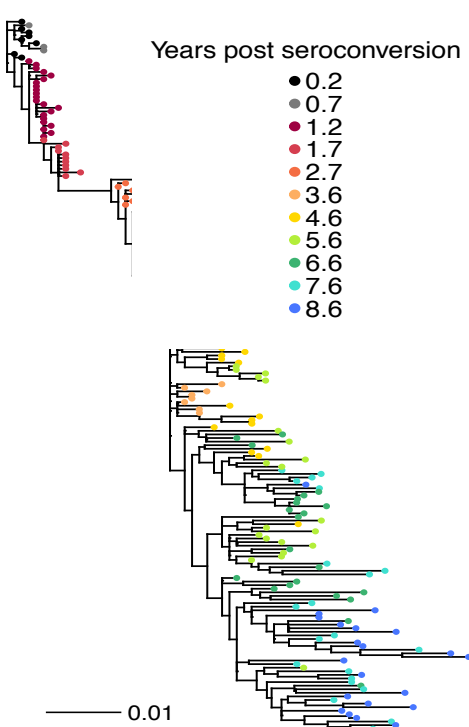

Figure S12 (con't)

**E. F5\_gag**

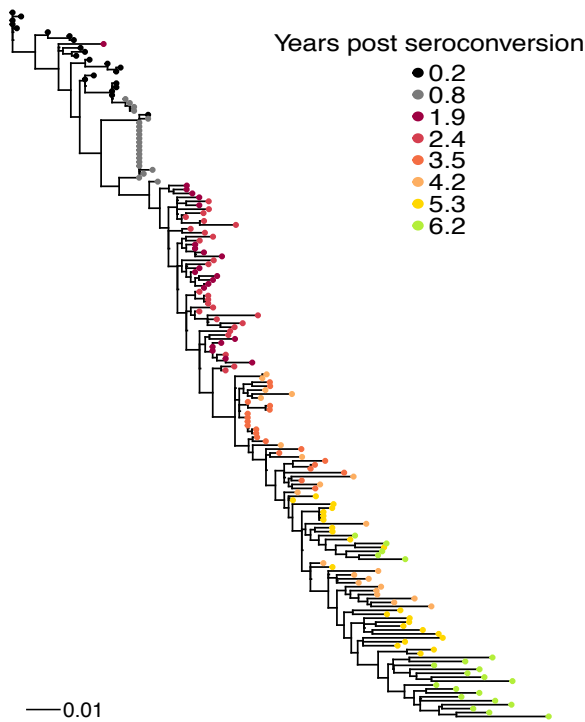

**F. F6\_gag**

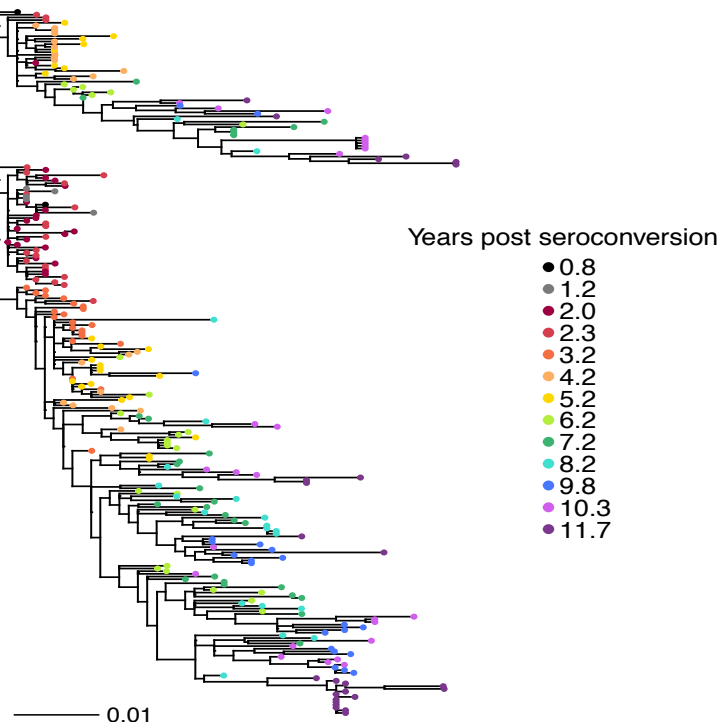

**G. F7\_gag**

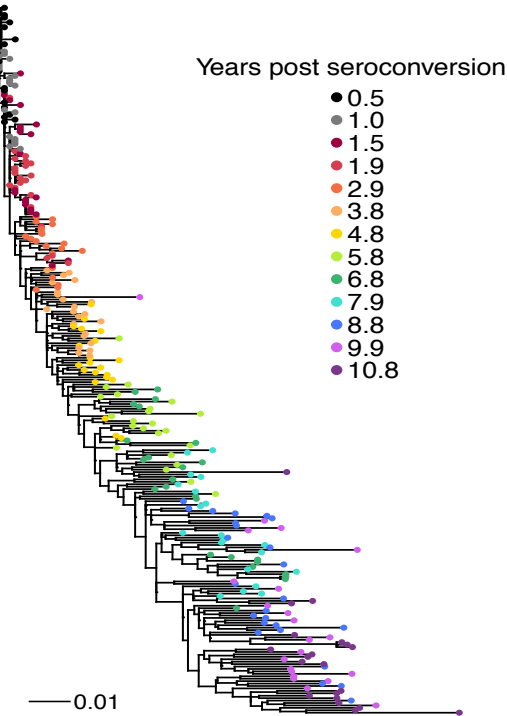

**H. F8\_gag**

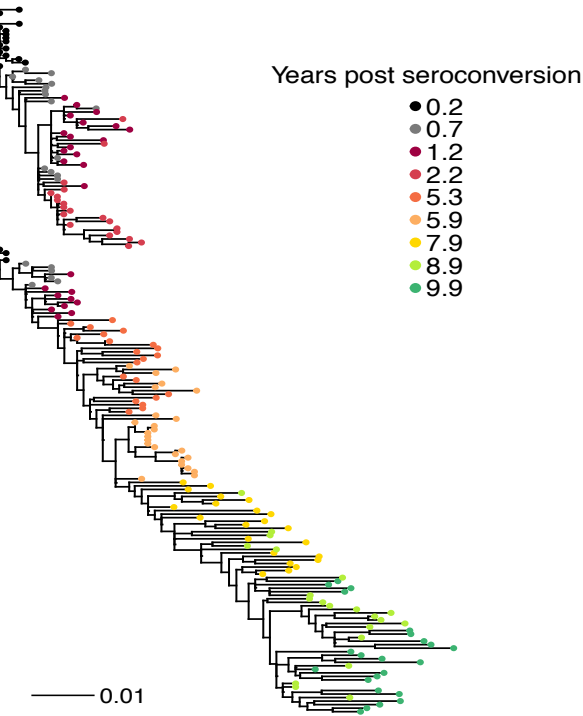

Supplement: S12 Fig — (A-H) Maximum likelihood gag phylogenetic trees of sequence from each participant were reconstructed using PhyML v3.0 (see Methods) and rooted to earliest timepoint sequences. Tip symbols show years post seroconversion (colored circles). The scale at the bottom measures genetic distances in nucleotide substitutions per site. (PDF) [file pone.0182443.s012.pdf]

Figure S13

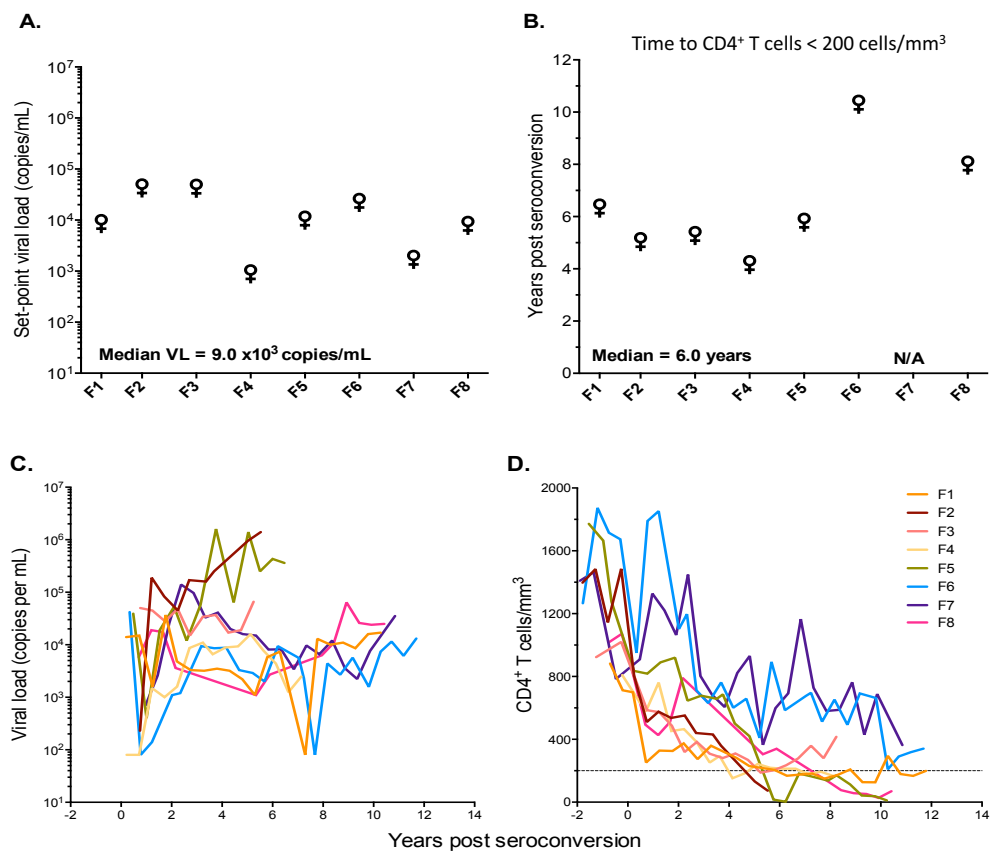

Supplement: S13 Fig — Summary plots of (A) Set-point viral load, (B) time to CD4+ T cells < 200 counts/mm3, (C) viral load, and (D) CD4+ T cell numbers are shown for each participant. N/A, not applicable because participant F7 did not reach CD4+ T cell count < 200. (PDF) [file pone.0182443.s013.pdf]
