# Supplementary material for: Patterns and rates of viral evolution in HIV-1 subtype B infected females and males
Source: PLoS One. 2017 Oct 18;12(10):e0182443. doi: 10.1371/journal.pone.0182443 (PMC5646779; doi:10.1371/journal.pone.0182443)

Figure S3

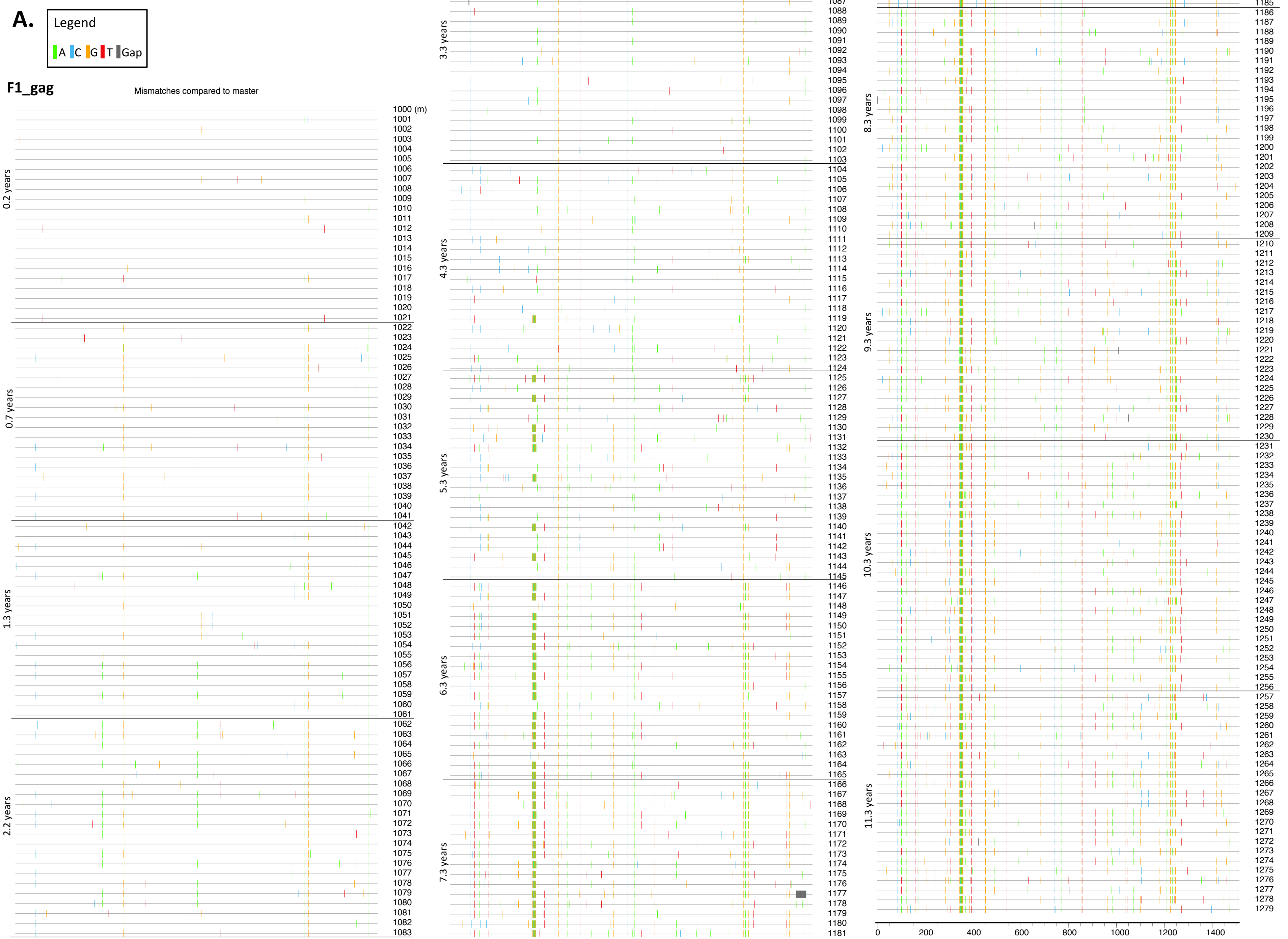
A.
Legend
A C G T Gap
F1_gag
Mismatches compared to master
1000 (m)
0.2 years
0.7 years
1.3 years
2.2 years
3.3 years
4.3 years
5.3 years
6.3 years
7.3 years
8.3 years
9.3 years
10.3 years
11.3 years
0
200
400
600
800
1000
1200
1400

Figure S3

B.

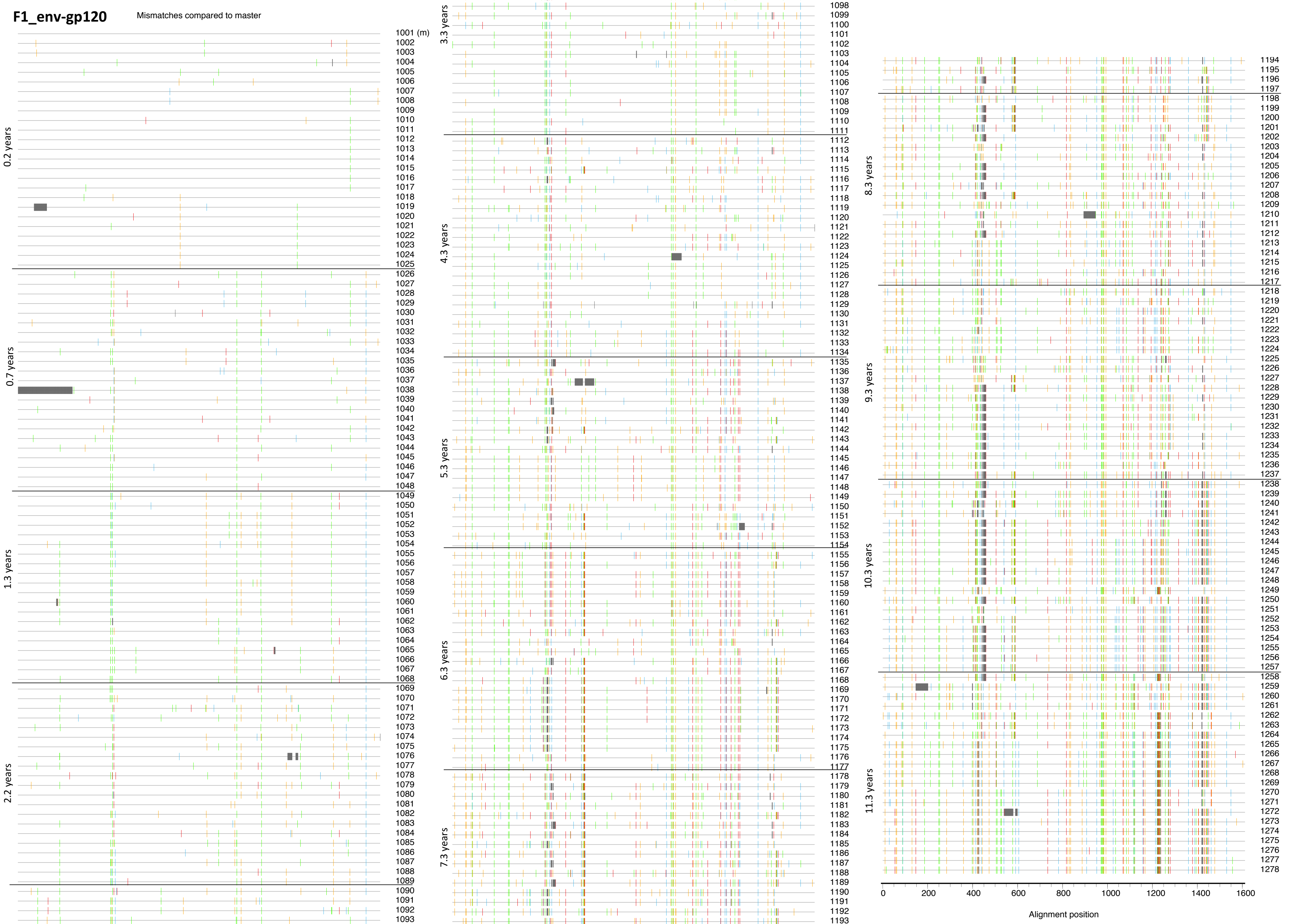
F1_env-gp120
Mismatches compared to master
0.2 years
0.7 years
1.3 years
2.2 years
3.3 years
4.3 years
5.3 years
6.3 years
7.3 years
8.3 years
9.3 years
10.3 years
11.3 years
1001 (m)
1002
1003
1004
1005
1006
1007
1008
1009
1010
1011
1012
1013
1014
1015
1016
1017
1018
1019
1020
1021
1022
1023
1024
1025
1026
1027
1028
1029
1030
1031
1032
1033
1034
1035
1036
1037
1038
1039
1040
1041
1042
1043
1044
1045
1046
1047
1048
1049
1050
1051
1052
1053
1054
1055
1056
1057
1058
1059
1060
1061
1062
1063
1064
1065
1066
1067
1068
1069
1070
1071
1072
1073
1074
1075
1076
1077
1078
1079
1080
1081
1082
1083
1084
1085
1086
1087
1088
1089
1090
1091
1092
1093
1093
1094
1095
1096
1097
1098
1099
1100
1101
1102
1103
1104
1105
1106
1107
1108
1109
1110
1111
1112
1113
1114
1115
1116
1117
1118
1119
1120
1121
1122
1123
1124
1125
1126
1127
1128
1129
1130
1131
1132
1133
1134
1135
1136
1137
1138
1139
1140
1141
1142
1143
1144
1145
1146
1147
1148
1149
1150
1151
1152
1153
1154
1155
1156
1157
1158
1159
1160
1161
1162
1163
1164
1165
1166
1167
1168
1169
1170
1171
1172
1173
1174
1175
1176
1177
1178
1179
1180
1181
1182
1183
1184
1185
1186
1187
1188
1189
1190
1191
1192
1193
1194
1195
1196
1197
1198
1199
1200
1201
1202
1203
1204
1205
1206
1207
1208
1209
1210
1211
1212
1213
1214
1215
1216
1217
1218
1219
1220
1221
1222
1223
1224
1225
1226
1227
1228
1229
1230
1231
1232
1233
1234
1235
1236
1237
1238
1239
1240
1241
1242
1243
1244
1245
1246
1247
1248
1249
1250
1251
1252
1253
1254
1255
1256
1257
1258
1259
1260
1261
1262
1263
1264
1265
1266
1267
1268
1269
1270
1271
1272
1273
1274
1275
1276
1277
1278
0
200
400
600
800
1000
1200
1400
1600
Alignment position

Figure S3
C.

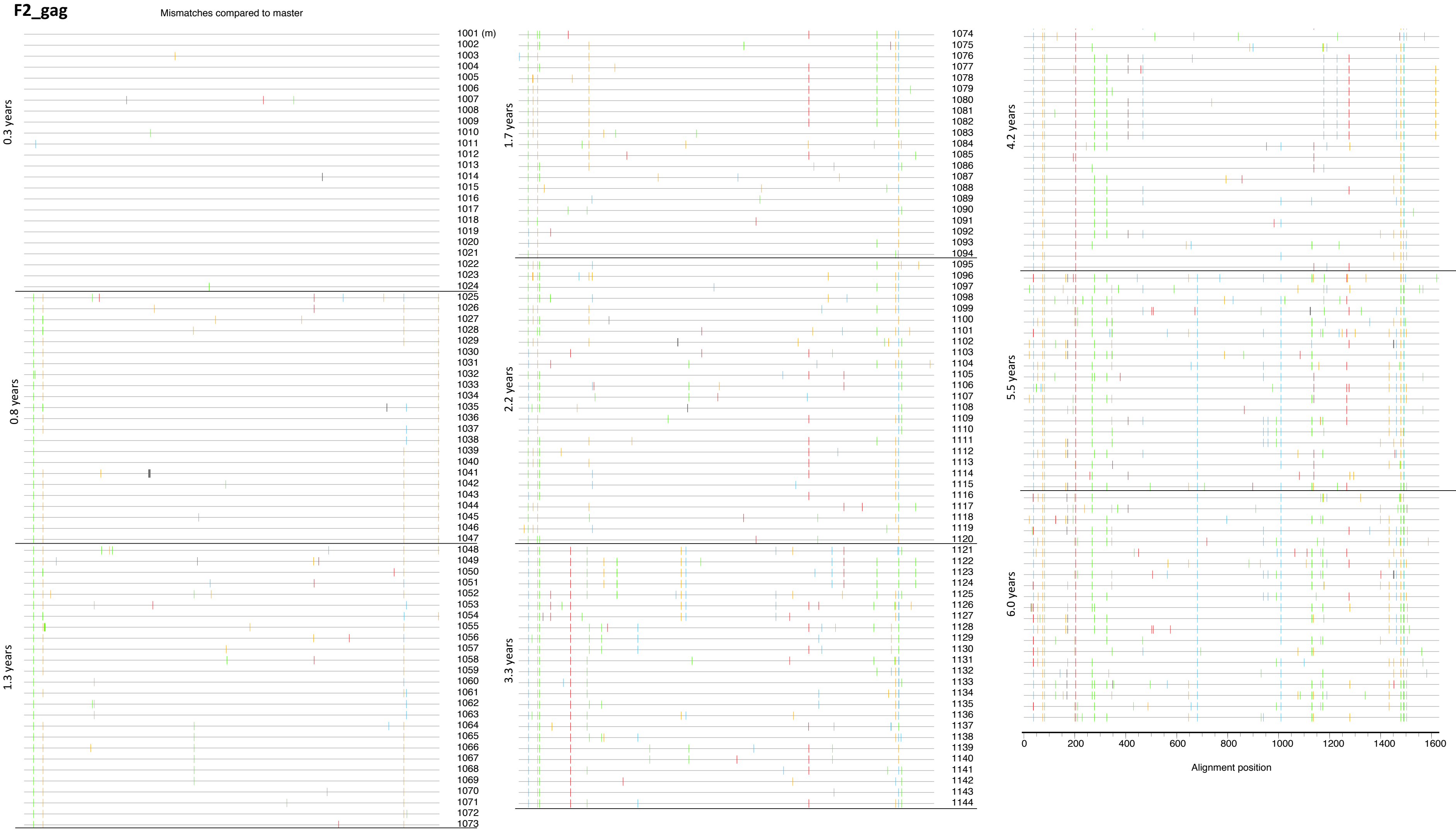
F2_gag
Mismatches compared to master
0.3 years
0.8 years
1.3 years
1.7 years
2.2 years
3.3 years
4.2 years
5.5 years
6.0 years
1001 (m)
1002
1003
1004
1005
1006
1007
1008
1009
1010
1011
1012
1013
1014
1015
1016
1017
1018
1019
1020
1021
1022
1023
1024
1025
1026
1027
1028
1029
1030
1031
1032
1033
1034
1035
1036
1037
1038
1039
1040
1041
1042
1043
1044
1045
1046
1047
1048
1049
1050
1051
1052
1053
1054
1055
1056
1057
1058
1059
1060
1061
1062
1063
1064
1065
1066
1067
1068
1069
1070
1071
1072
1073
1074
1075
1076
1077
1078
1079
1080
1081
1082
1083
1084
1085
1086
1087
1088
1089
1090
1091
1092
1093
1094
1095
1096
1097
1098
1099
1100
1101
1102
1103
1104
1105
1106
1107
1108
1109
1110
1111
1112
1113
1114
1115
1116
1117
1118
1119
1120
1121
1122
1123
1124
1125
1126
1127
1128
1129
1130
1131
1132
1133
1134
1135
1136
1137
1138
1139
1140
1141
1142
1143
1144
1145
1146
1147
1148
1149
1150
1151
1152
1153
1154
1155
1156
1157
1158
1159
1160
1161
1162
1163
1164
1165
1166
1167
1168
1169
1170
1171
1172
1173
1174
1175
1176
1177
1178
1179
1180
1181
1182
1183
1184
1185
1186
1187
1188
1189
1190
1191
1192
1193
1194
1195
1196
1197
1198
1199
1200
1201
1202
1203
1204
1205
1206
1207
0
200
400
600
800
1000
1200
1400
1600
Alignment position

Figure S3

D.

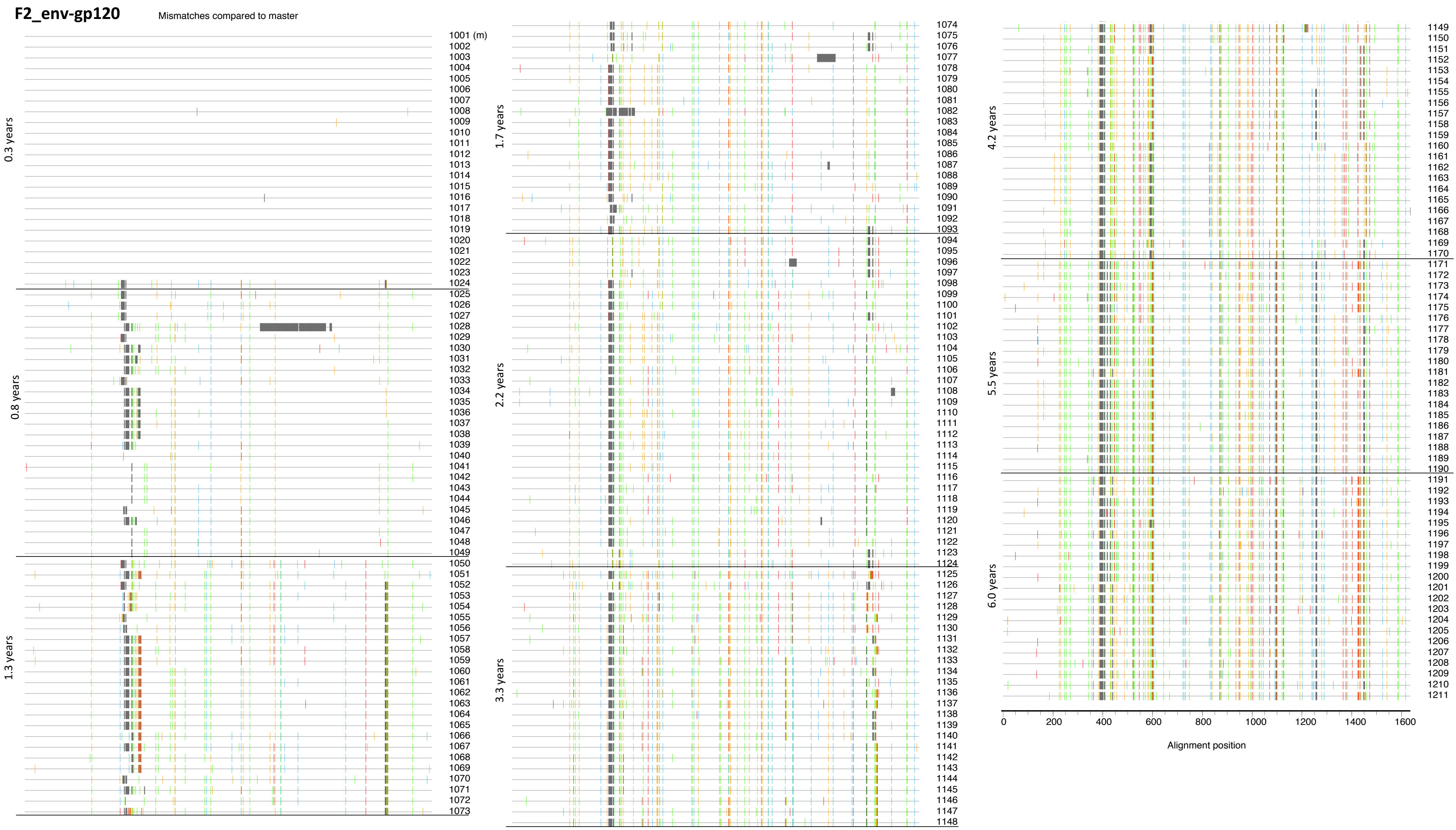
F2_env-gp120
Mismatches compared to master
0.3 years
0.8 years
1.3 years
1.7 years
2.2 years
3.3 years
4.2 years
5.5 years
6.0 years
1001 (m)
1002
1003
1004
1005
1006
1007
1008
1009
1010
1011
1012
1013
1014
1015
1016
1017
1018
1019
1020
1021
1022
1023
1024
1025
1026
1027
1028
1029
1030
1031
1032
1033
1034
1035
1036
1037
1038
1039
1040
1041
1042
1043
1044
1045
1046
1047
1048
1049
1050
1051
1052
1053
1054
1055
1056
1057
1058
1059
1060
1061
1062
1063
1064
1065
1066
1067
1068
1069
1070
1071
1072
1073
1074
1075
1076
1077
1078
1079
1080
1081
1082
1083
1084
1085
1086
1087
1088
1089
1090
1091
1092
1093
1094
1095
1096
1097
1098
1099
1100
1101
1102
1103
1104
1105
1106
1107
1108
1109
1110
1111
1112
1113
1114
1115
1116
1117
1118
1119
1120
1121
1122
1123
1124
1125
1126
1127
1128
1129
1130
1131
1132
1133
1134
1135
1136
1137
1138
1139
1140
1141
1142
1143
1144
1145
1146
1147
1148
1149
1150
1151
1152
1153
1154
1155
1156
1157
1158
1159
1160
1161
1162
1163
1164
1165
1166
1167
1168
1169
1170
1171
1172
1173
1174
1175
1176
1177
1178
1179
1180
1181
1182
1183
1184
1185
1186
1187
1188
1189
1190
1191
1192
1193
1194
1195
1196
1197
1198
1199
1200
1201
1202
1203
1204
1205
1206
1207
1208
1209
1210
1211
0
200
400
600
800
1000
1200
1400
1600
Alignment position

Figure S3
E.

F3_gag

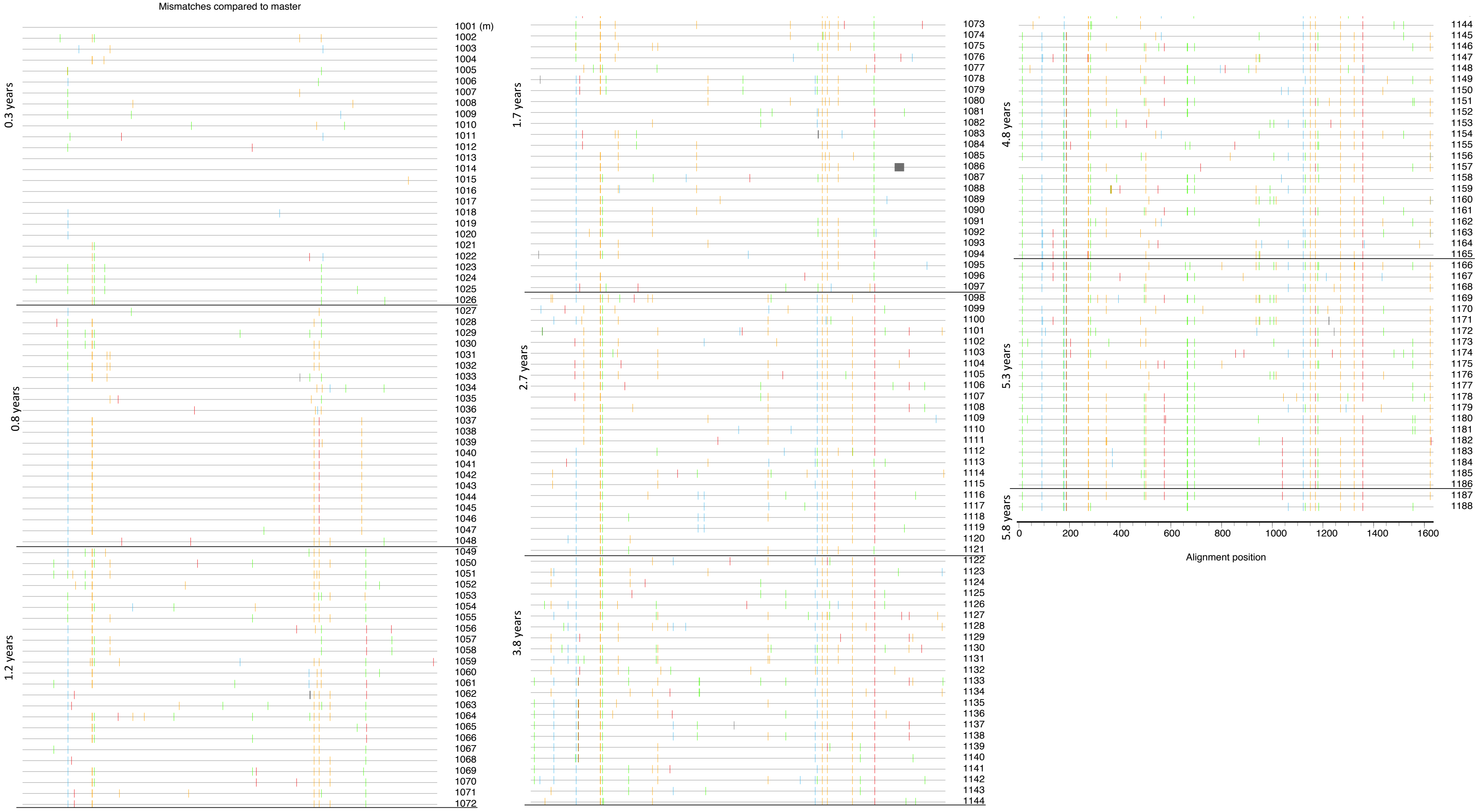

Figure S3
F.

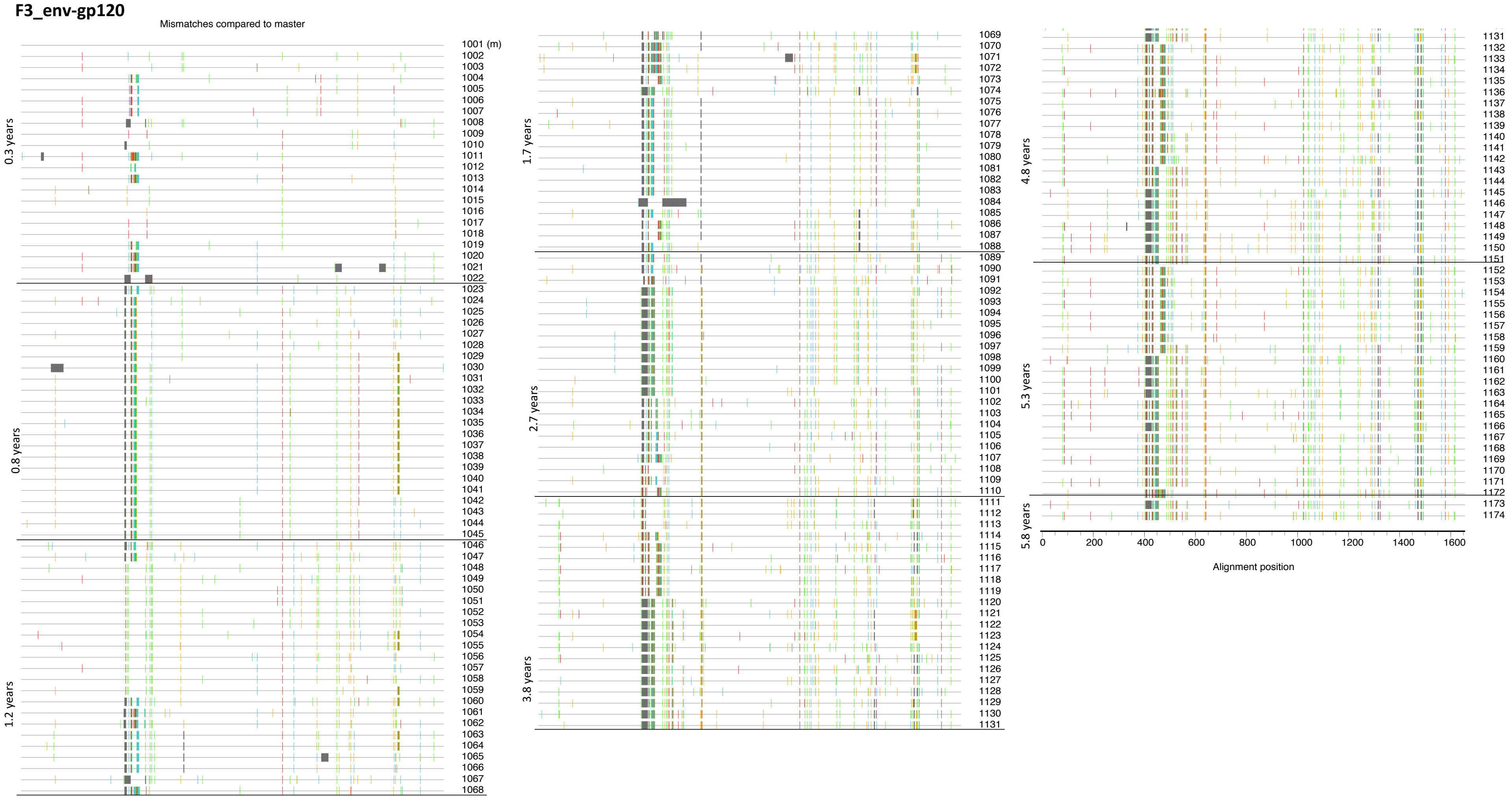
F3_env-gp120
Mismatches compared to master
0.3 years
0.8 years
1.2 years
1.7 years
2.7 years
3.8 years
4.8 years
5.3 years
5.8 years
1001 (m)
0
200
400
600
800
1000
1200
1400
1600
Alignment position

Figure S3

G.

F4_gag

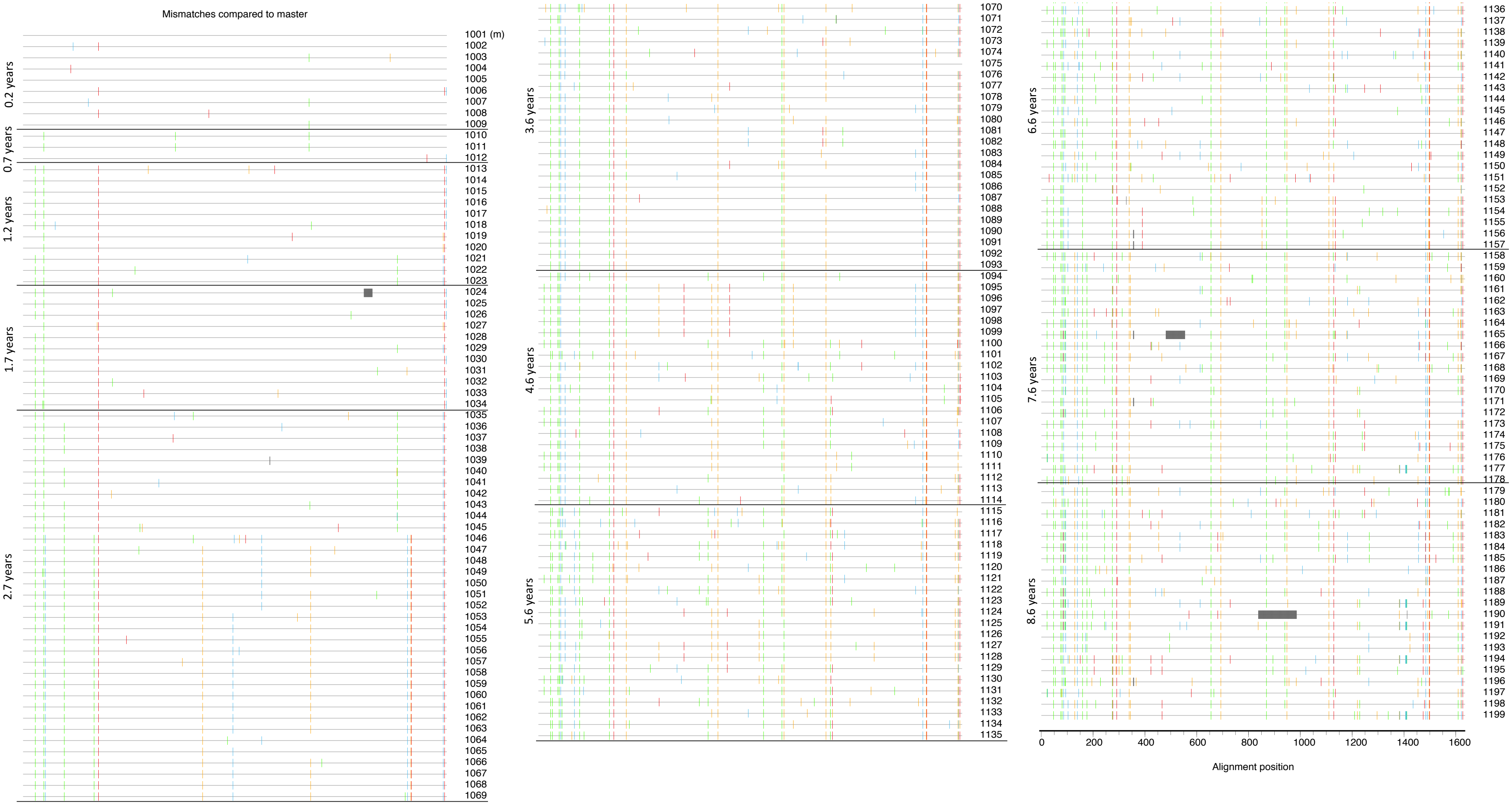

Figure S3
H.

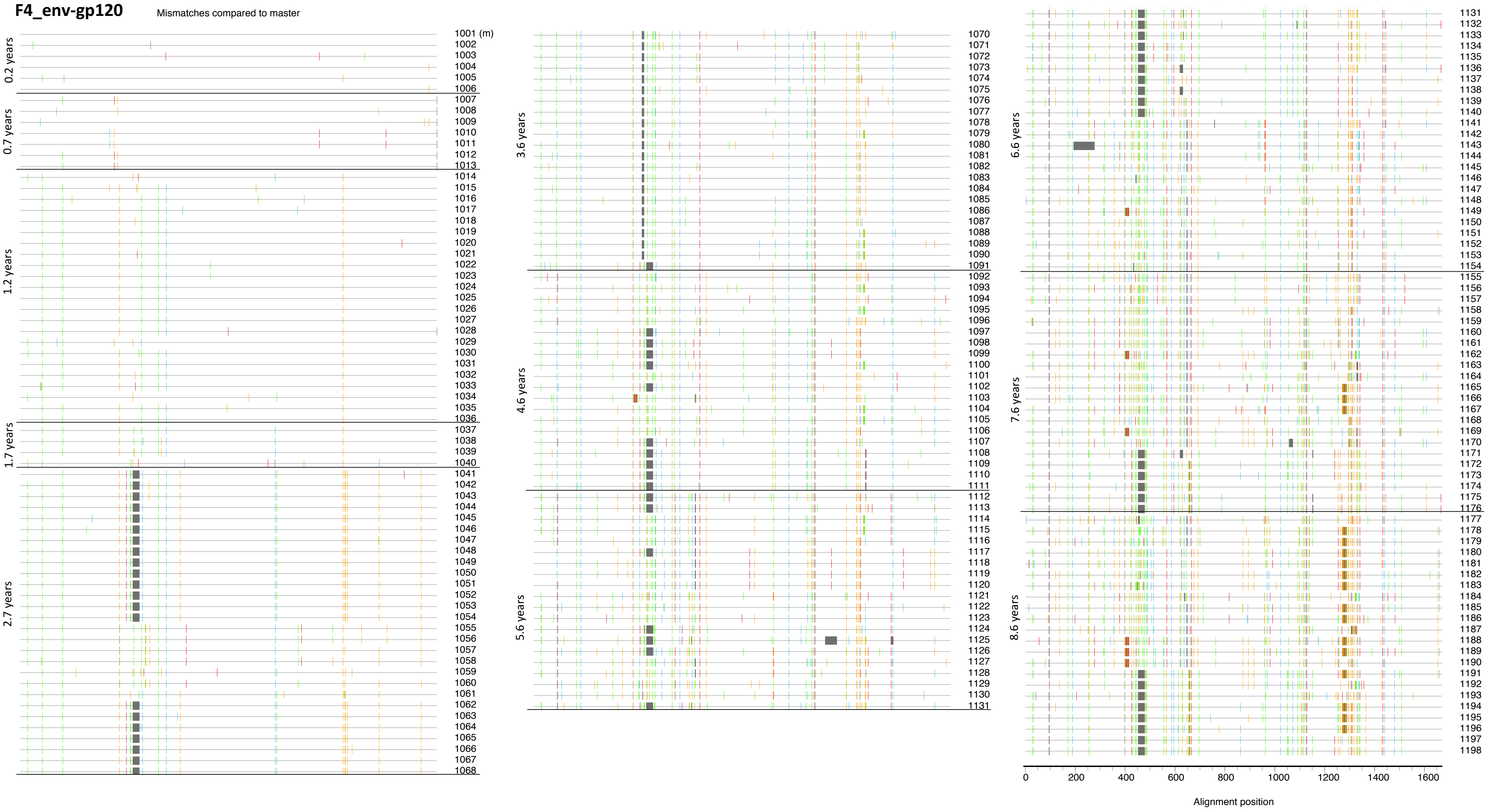

F4_env-gp120
Mismatches compared to master
1001 (m)
0.2 years
0.7 years
1.2 years
1.7 years
2.7 years
3.6 years
4.6 years
5.6 years
6.6 years
7.6 years
8.6 years
0
200
400
600
800
1000
1200
1400
1600
Alignment position

Figure S3

I.

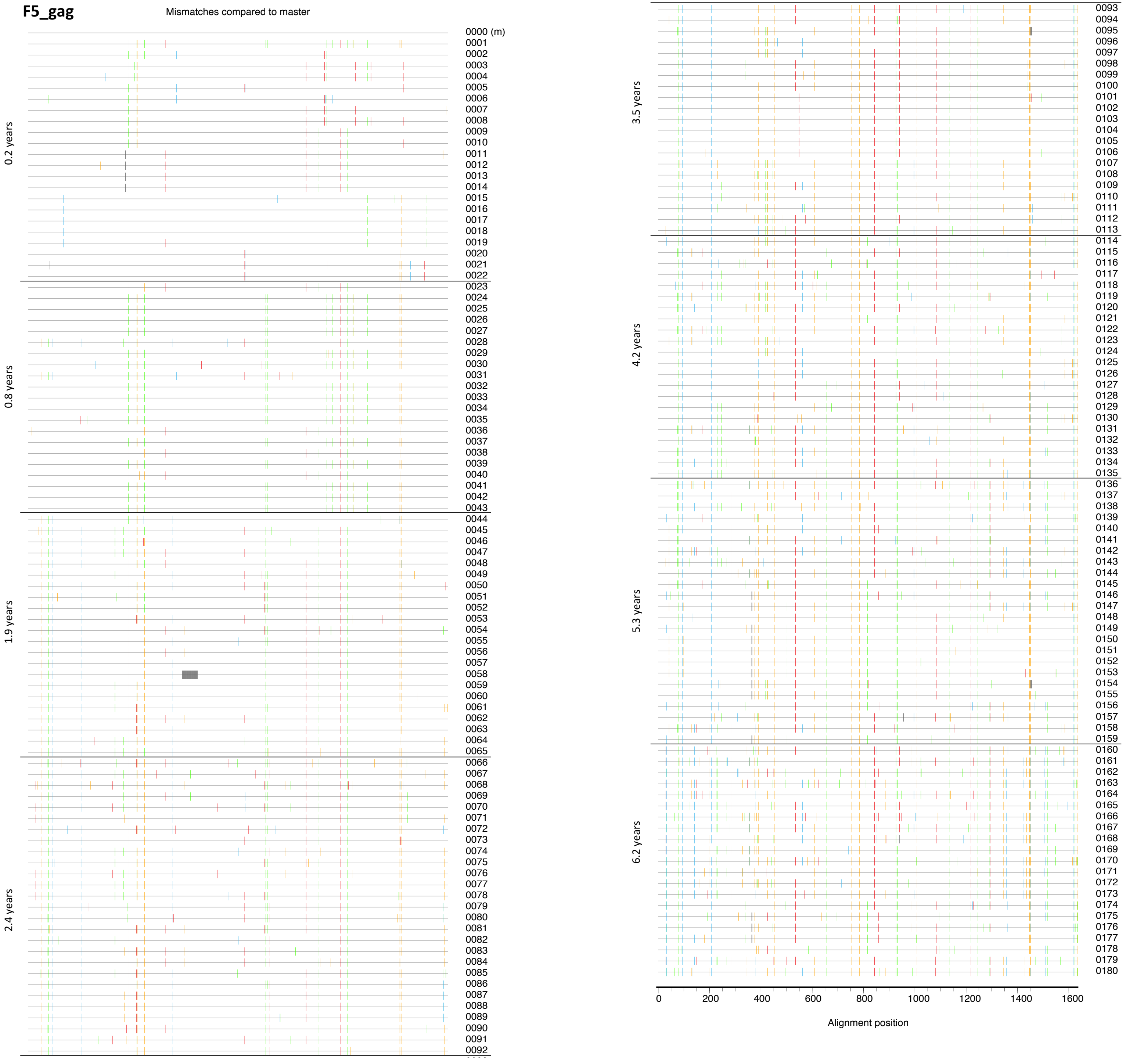
F5_gag
Mismatches compared to master
0.2 years
0.8 years
1.9 years
2.4 years
3.5 years
4.2 years
5.3 years
6.2 years
0000 (m)
0 200 400 600 800 1000 1200 1400 1600
Alignment position

Figure S3

J.

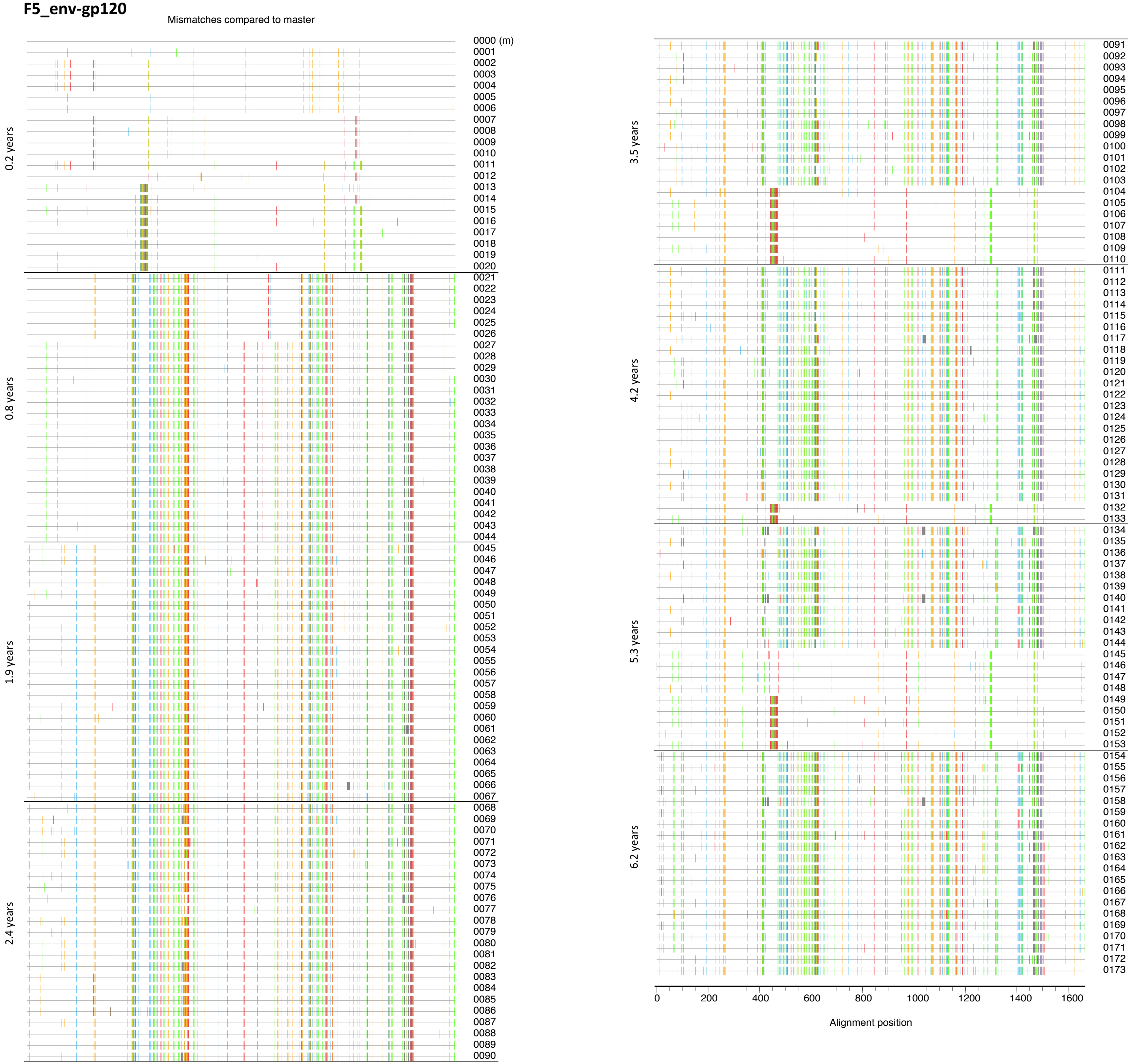

F5_env-gp120
Mismatches compared to master
0000 (m)
0.2 years
0.8 years
1.9 years
2.4 years
3.5 years
4.2 years
5.3 years
6.2 years
0 200 400 600 800 1000 1200 1400 1600
Alignment position

Figure S3
K.

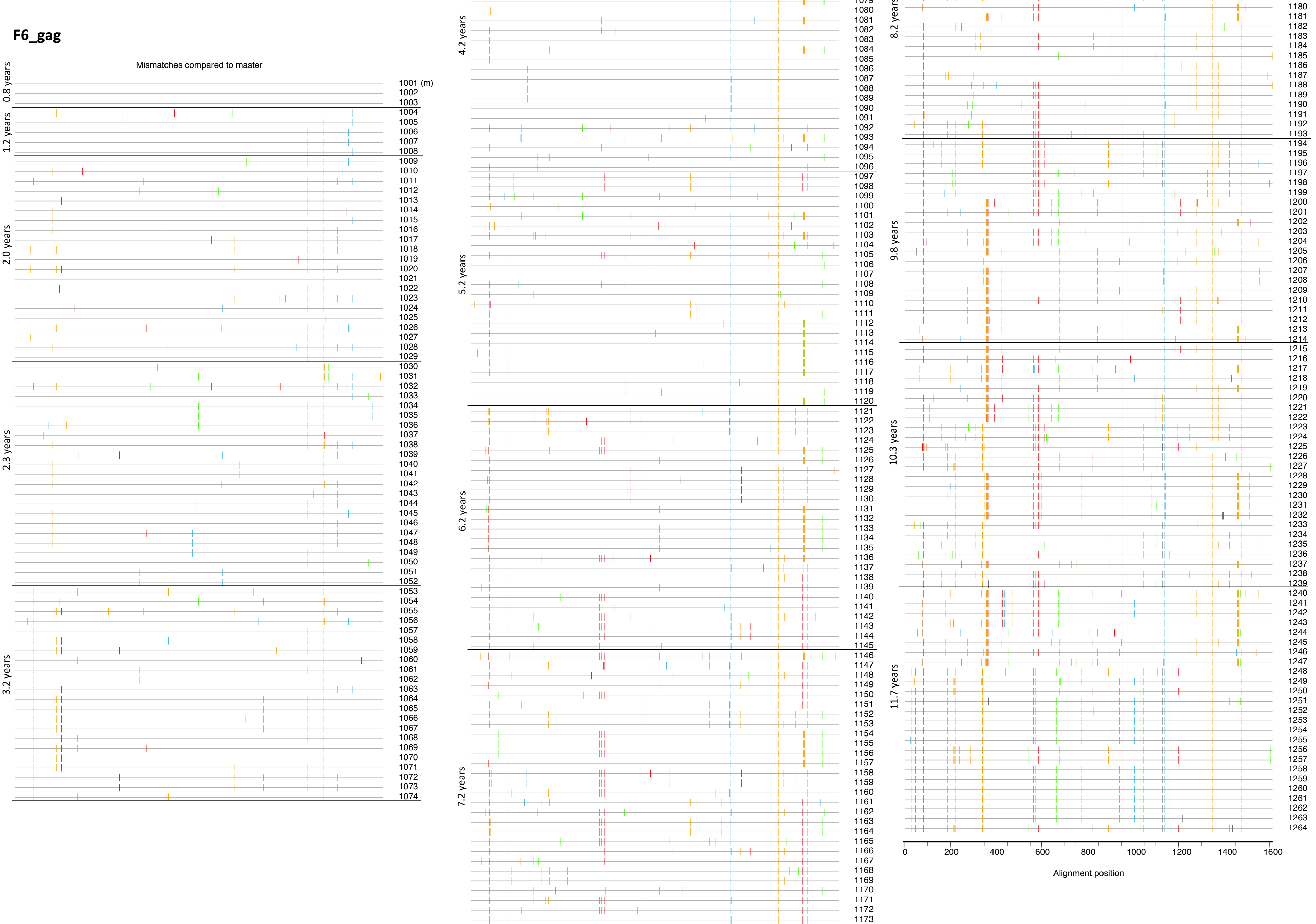
F6_gag
Mismatches compared to master
0.8 years
1.2 years
2.0 years
2.3 years
3.2 years
4.2 years
5.2 years
6.2 years
7.2 years
8.2 years
9.8 years
10.3 years
11.7 years
1001 (m)
Alignment position
0
200
400
600
800
1000
1200
1400
1600

Figure S3
L.

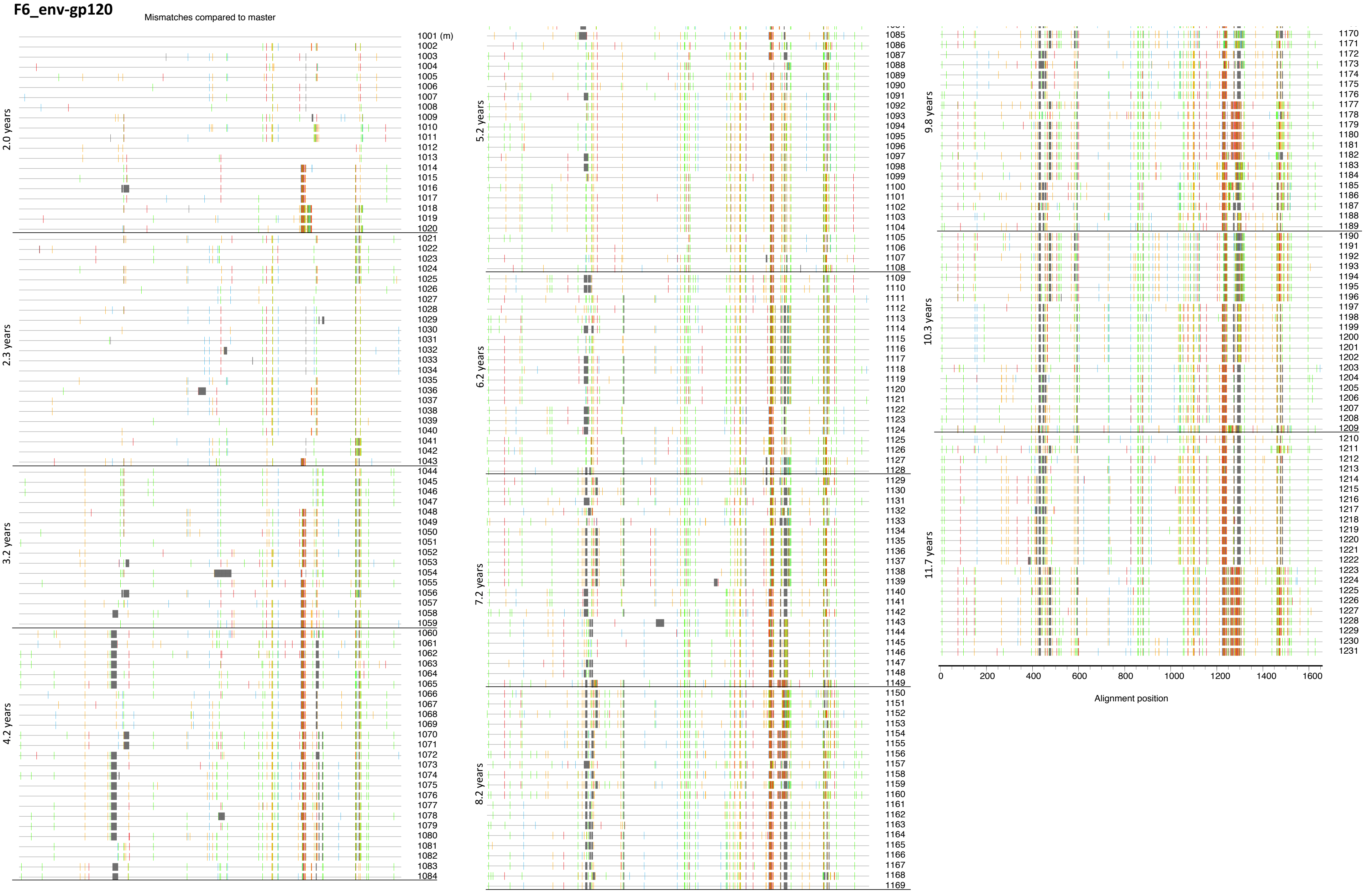
F6_env-gp120
Mismatches compared to master
2.0 years
2.3 years
3.2 years
4.2 years
5.2 years
6.2 years
7.2 years
8.2 years
9.8 years
10.3 years
11.7 years
1001 (m)
1002
1003
1004
1005
1006
1007
1008
1009
1010
1011
1012
1013
1014
1015
1016
1017
1018
1019
1020
1021
1022
1023
1024
1025
1026
1027
1028
1029
1030
1031
1032
1033
1034
1035
1036
1037
1038
1039
1040
1041
1042
1043
1044
1045
1046
1047
1048
1049
1050
1051
1052
1053
1054
1055
1056
1057
1058
1059
1060
1061
1062
1063
1064
1065
1066
1067
1068
1069
1070
1071
1072
1073
1074
1075
1076
1077
1078
1079
1080
1081
1082
1083
1084
1085
1086
1087
1088
1089
1090
1091
1092
1093
1094
1095
1096
1097
1098
1099
1100
1101
1102
1103
1104
1105
1106
1107
1108
1109
1110
1111
1112
1113
1114
1115
1116
1117
1118
1119
1120
1121
1122
1123
1124
1125
1126
1127
1128
1129
1130
1131
1132
1133
1134
1135
1136
1137
1138
1139
1140
1141
1142
1143
1144
1145
1146
1147
1148
1149
1150
1151
1152
1153
1154
1155
1156
1157
1158
1159
1160
1161
1162
1163
1164
1165
1166
1167
1168
1169
1170
1171
1172
1173
1174
1175
1176
1177
1178
1179
1180
1181
1182
1183
1184
1185
1186
1187
1188
1189
1190
1191
1192
1193
1194
1195
1196
1197
1198
1199
1200
1201
1202
1203
1204
1205
1206
1207
1208
1209
1210
1211
1212
1213
1214
1215
1216
1217
1218
1219
1220
1221
1222
1223
1224
1225
1226
1227
1228
1229
1230
1231
0
200
400
600
800
1000
1200
1400
1600
Alignment position

Figure S3

M.

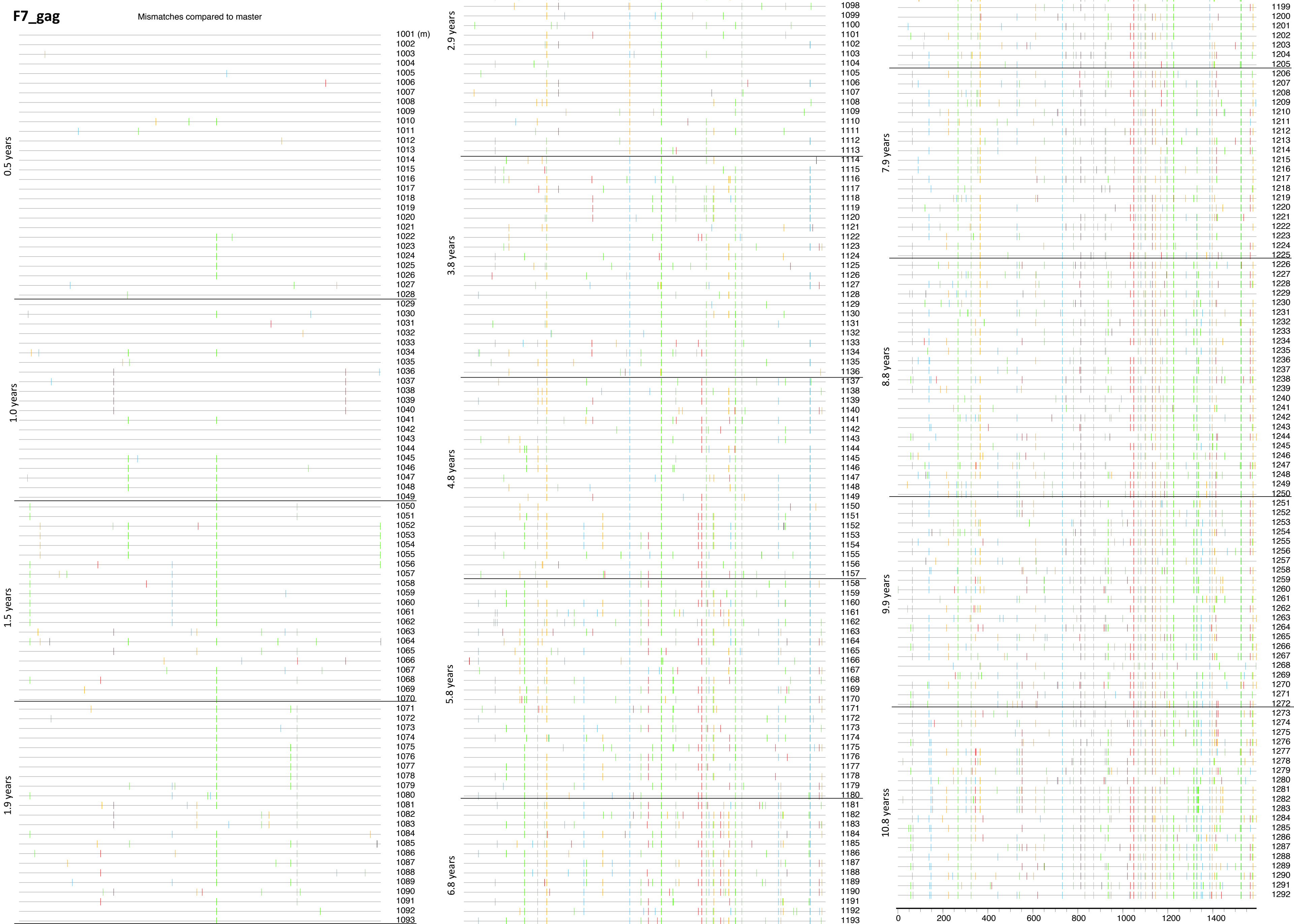
F7_gag
Mismatches compared to master
1001 (m)
0.5 years
1.0 years
1.5 years
1.9 years
2.9 years
3.8 years
4.8 years
5.8 years
6.8 years
7.9 years
8.8 years
9.9 years
10.8 yearss
0
200
400
600
800
1000
1200
1400

Figure S3

N.

F7_env-gp120

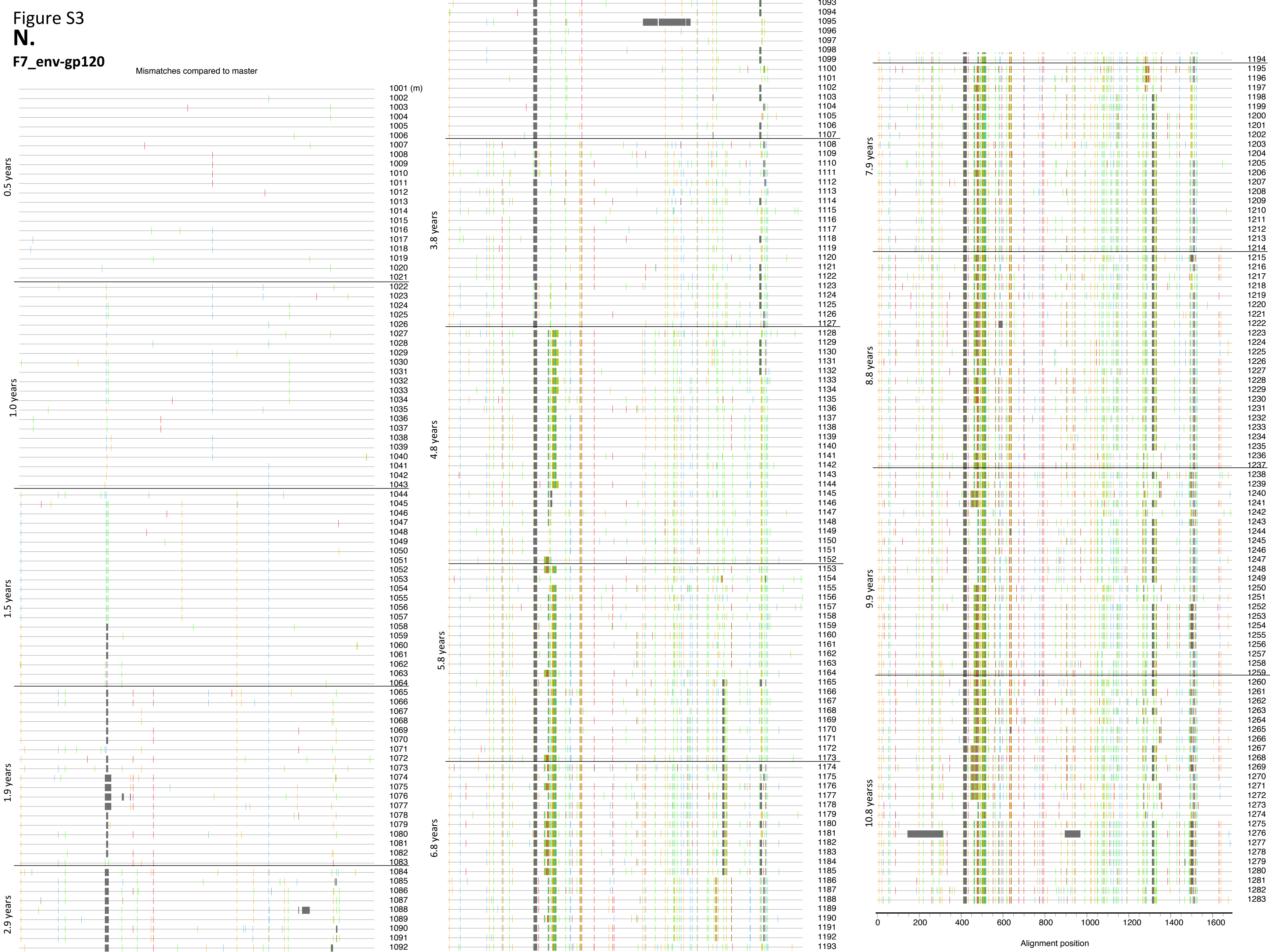

Figure S3
O.

F8_gag

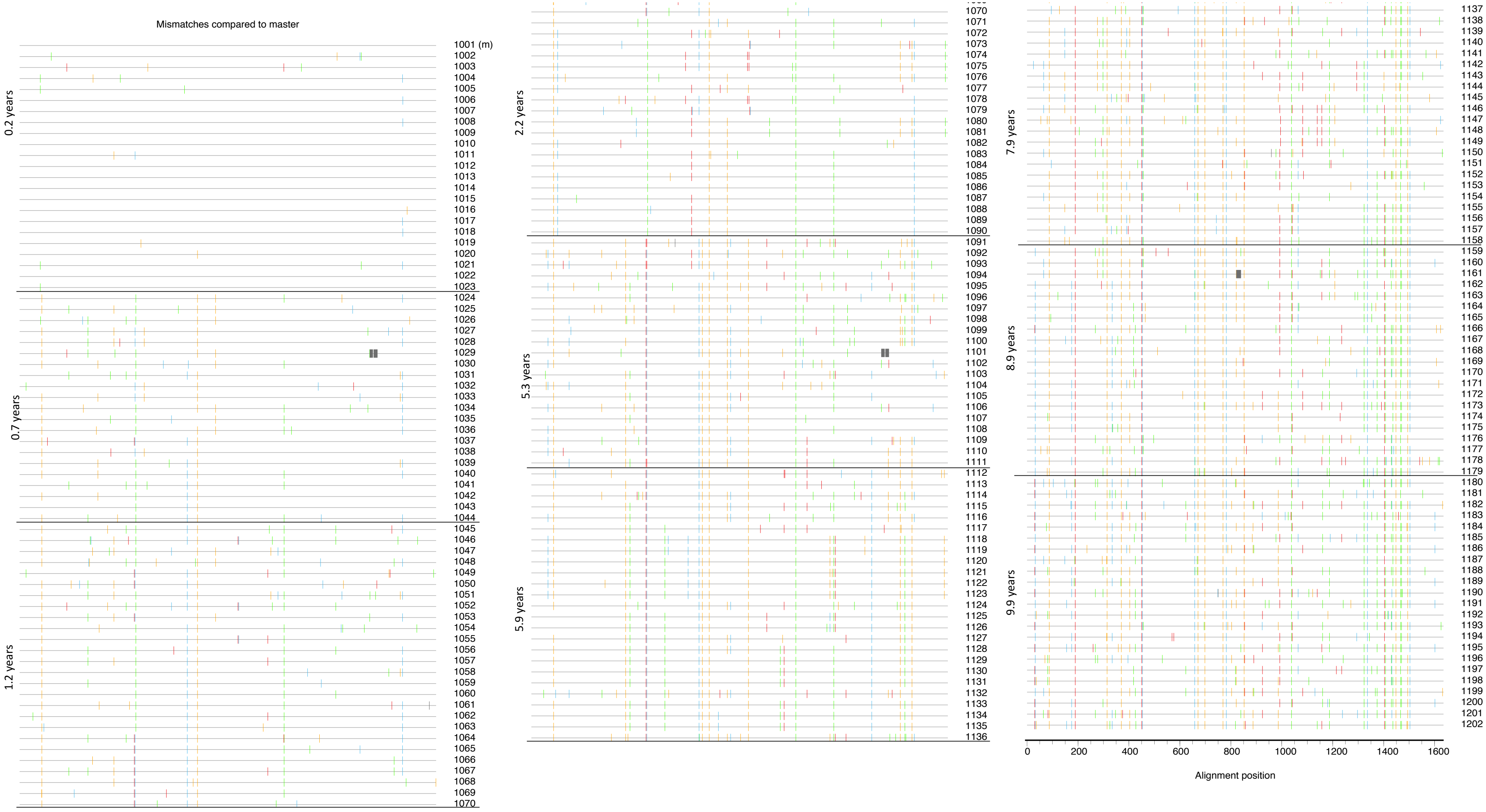

Figure S3
P.

F8_env-gp120

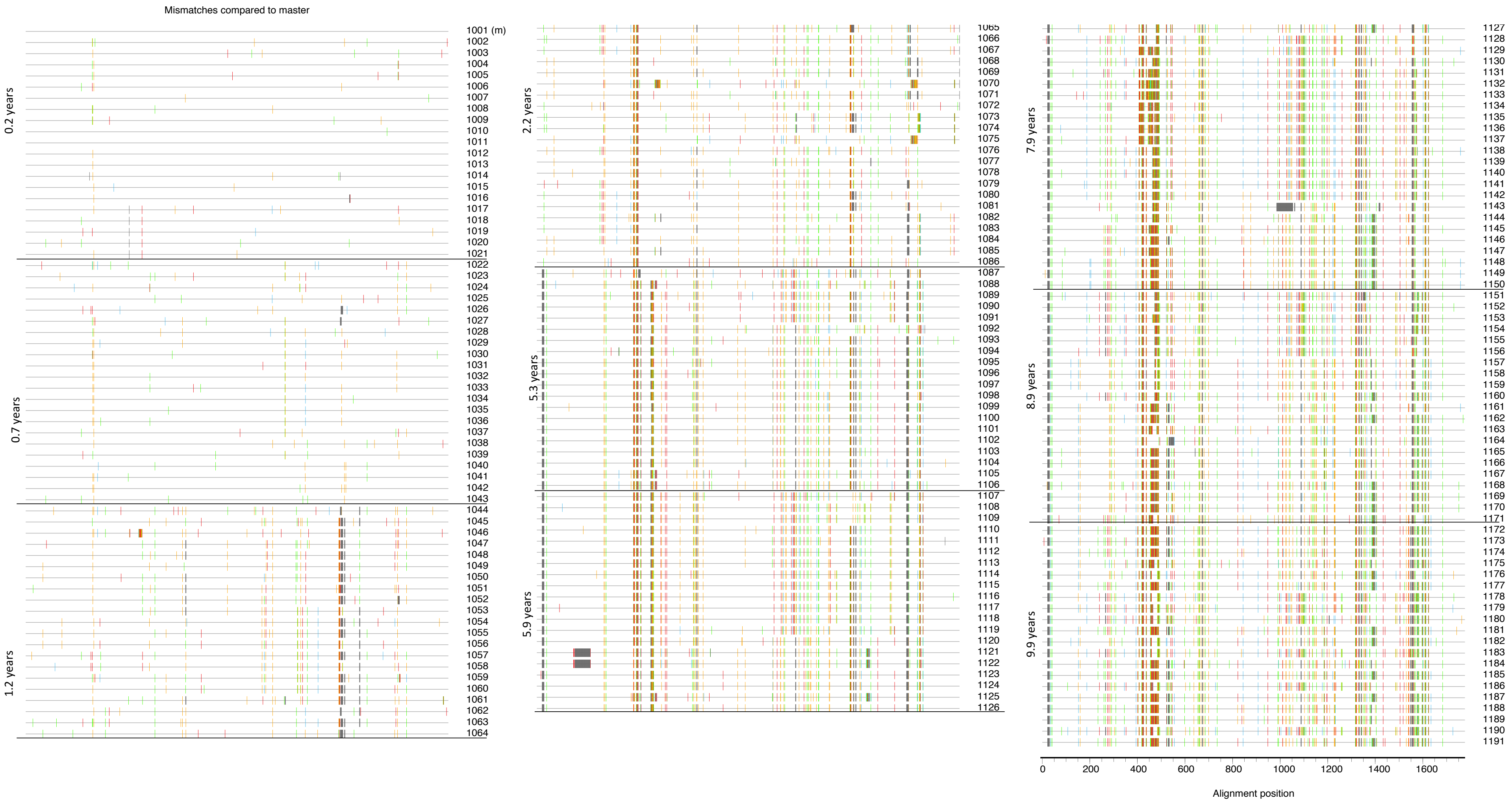

Figure S3

Q.

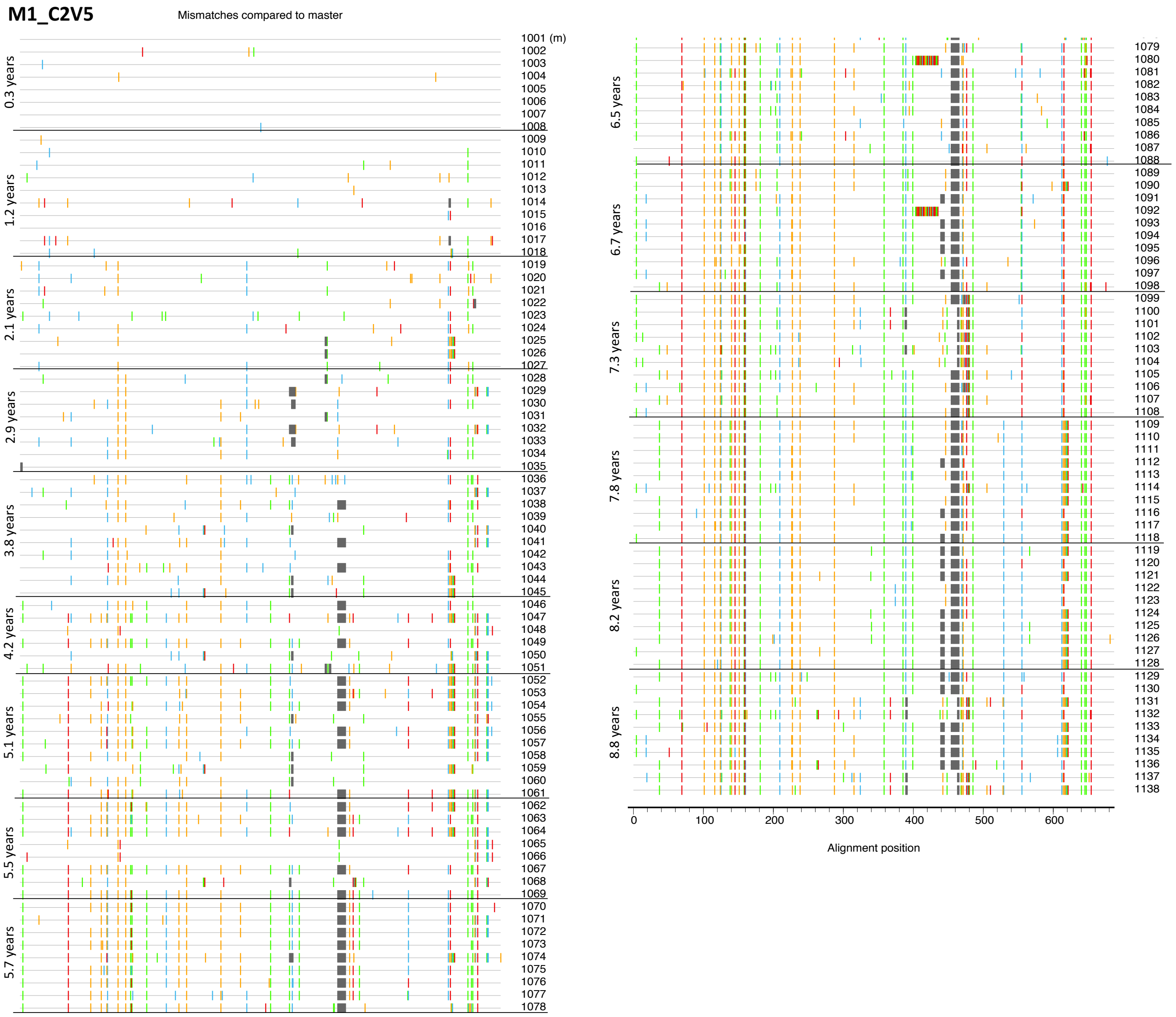

M1_C2V5
Mismatches compared to master
0.3 years
1.2 years
2.1 years
2.9 years
3.8 years
4.2 years
5.1 years
5.5 years
5.7 years
6.5 years
6.7 years
7.3 years
7.8 years
8.2 years
8.8 years
1001 (m)
0
100
200
300
400
500
600
Alignment position

Figure S3
R.

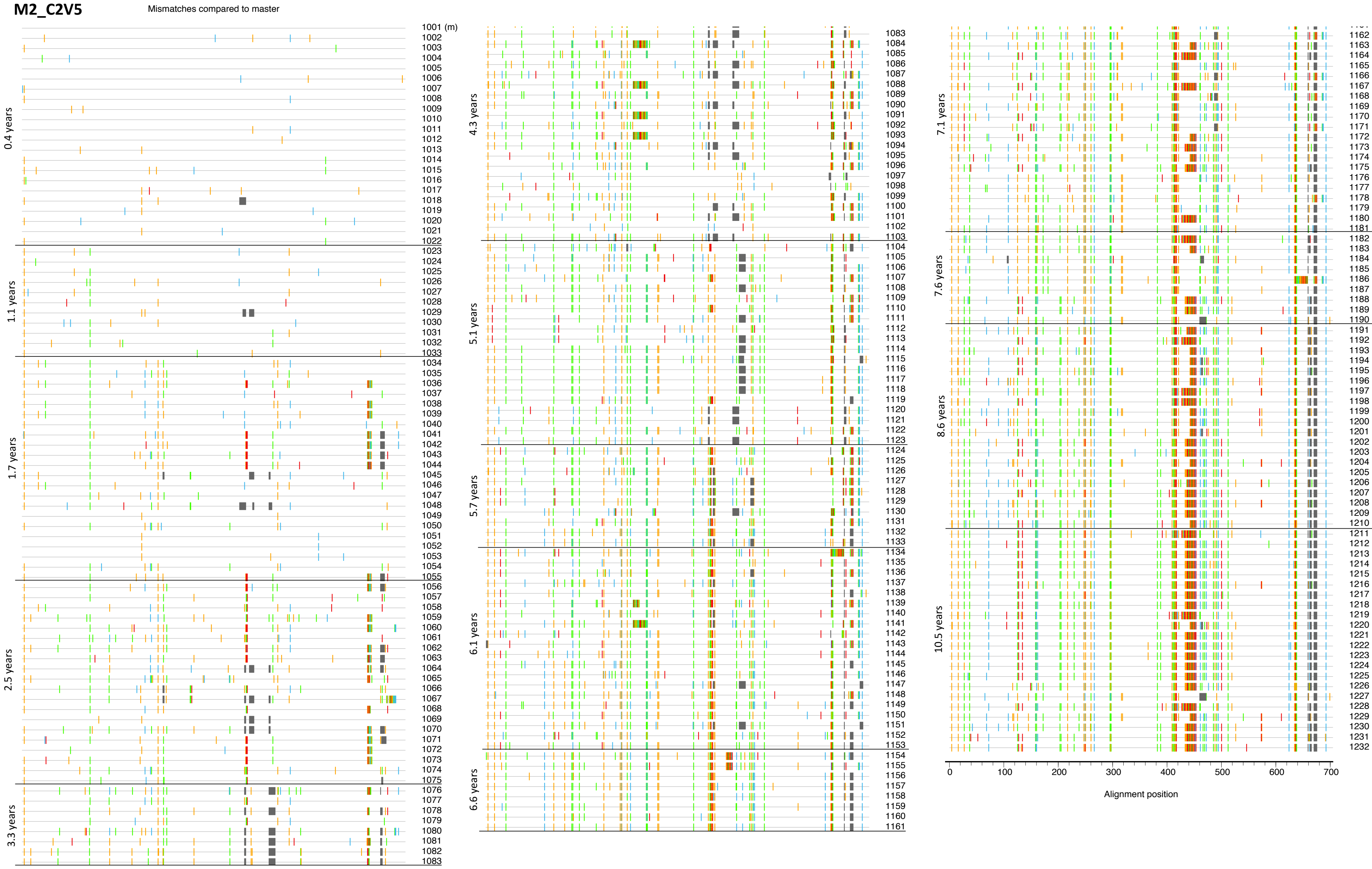
M2_C2V5
Mismatches compared to master
1001 (m)
0.4 years
1.1 years
1.7 years
2.5 years
3.3 years
4.3 years
5.1 years
5.7 years
6.1 years
6.6 years
7.1 years
7.6 years
8.6 years
10.5 years
0
100
200
300
400
500
600
700
Alignment position

Figure S3

S.

M3_C2V5

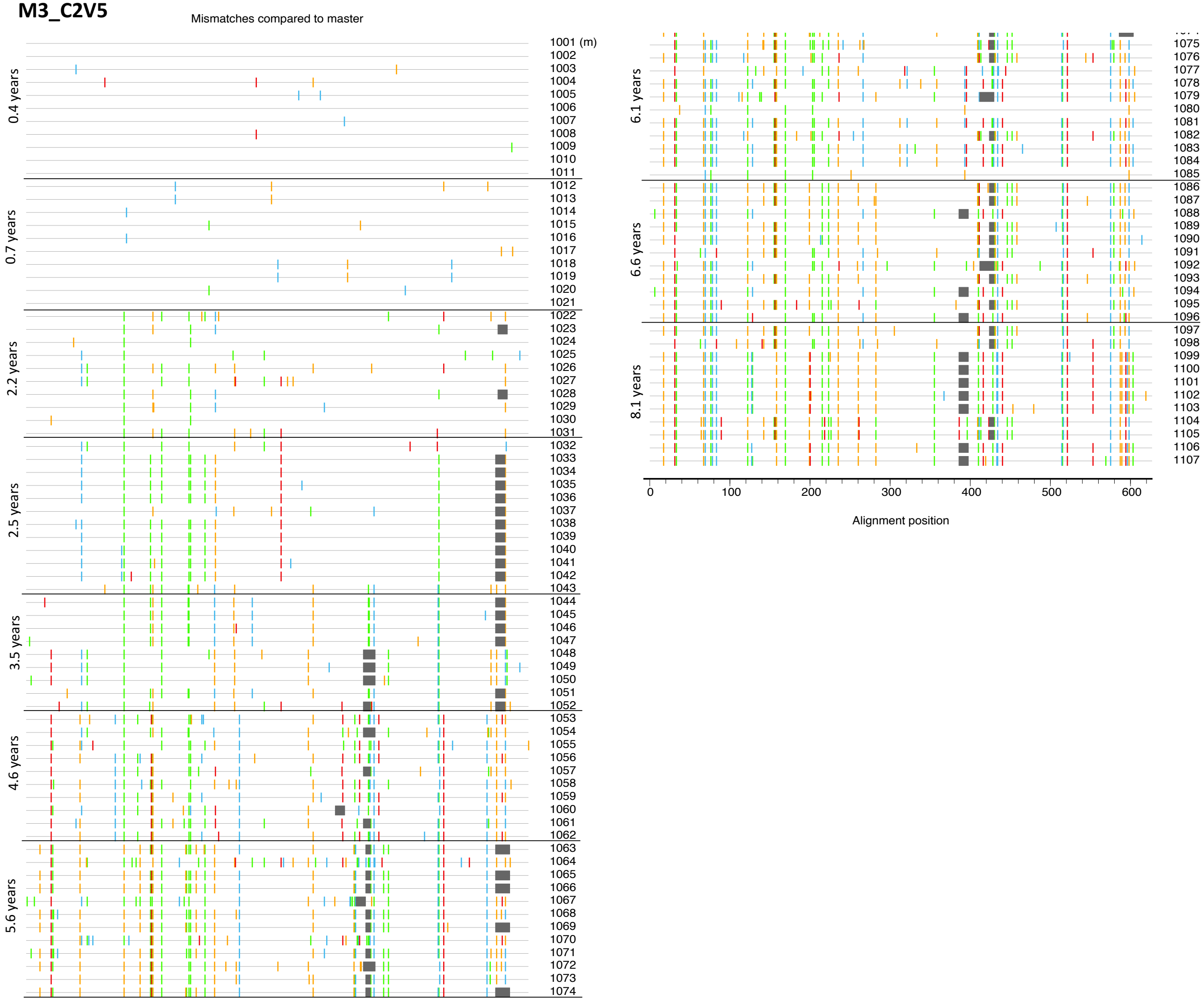

Figure S3

T.

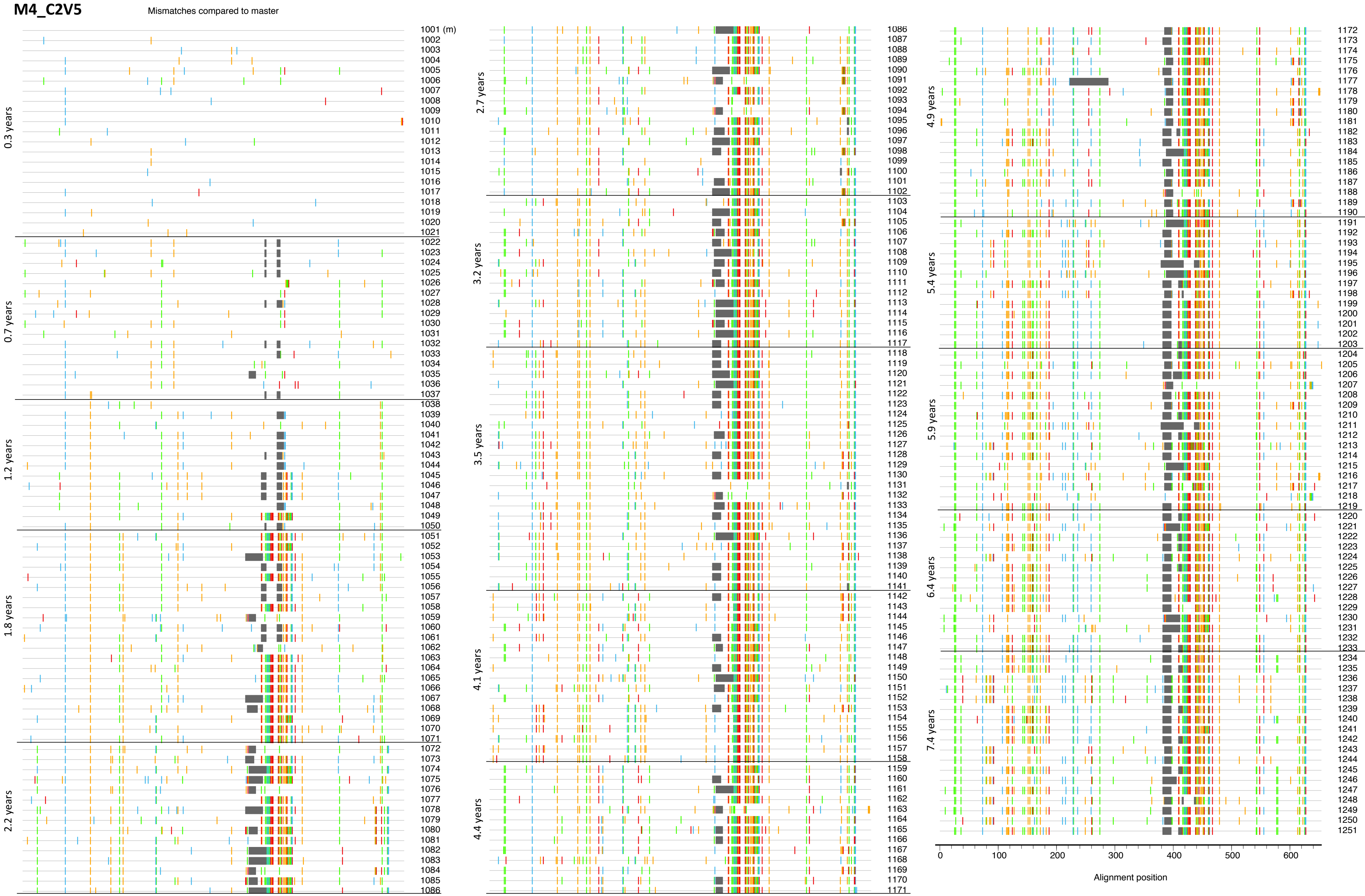
M4_C2V5
Mismatches compared to master
1001 (m)
0.3 years
0.7 years
1.2 years
1.8 years
2.2 years
2.7 years
3.2 years
3.5 years
4.1 years
4.4 years
4.9 years
5.4 years
5.9 years
6.4 years
7.4 years
0
100
200
300
400
500
600
Alignment position

Figure S3

U.

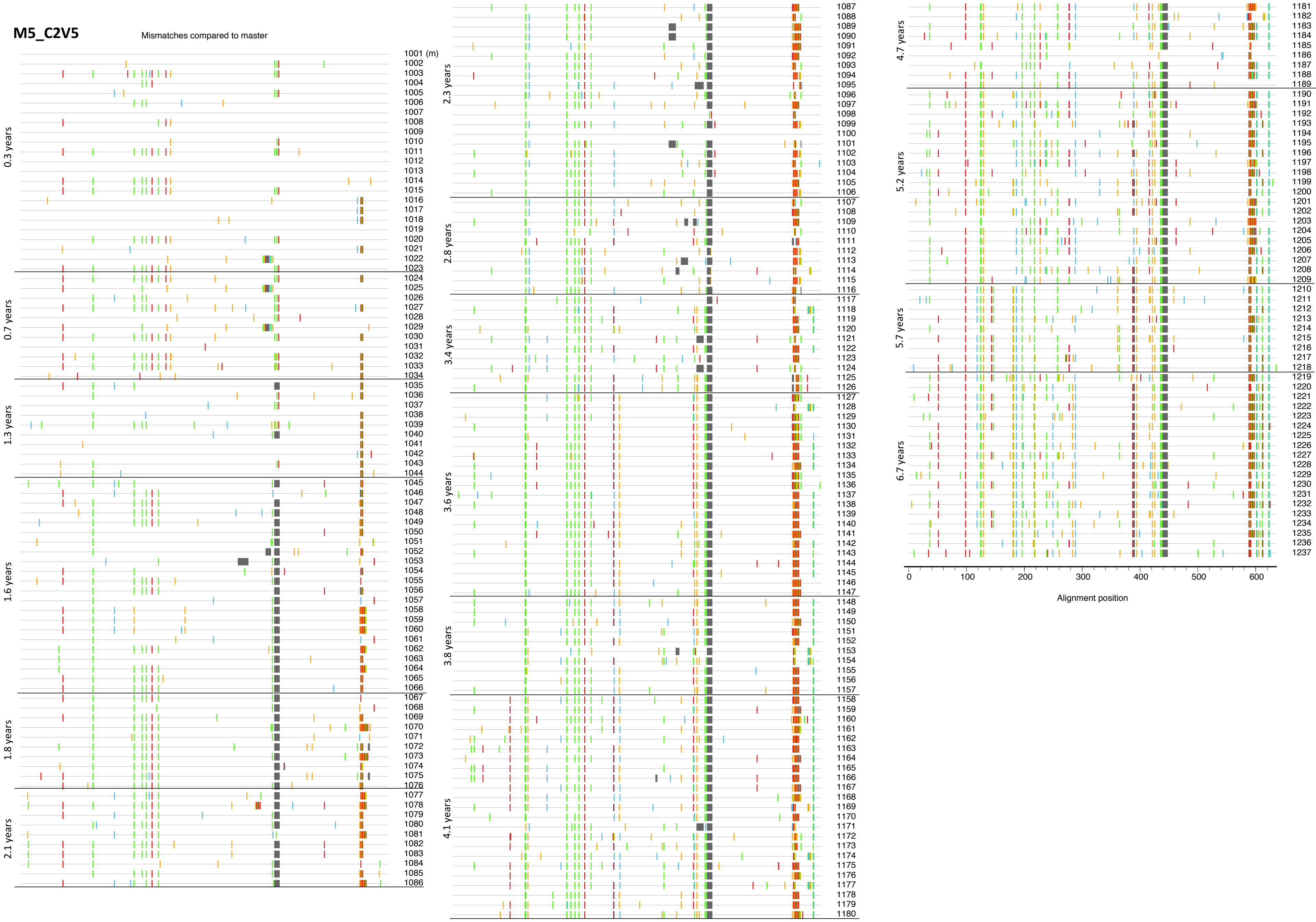

M5_C2V5
Mismatches compared to master
1001 (m)
0.3 years
0.7 years
1.3 years
1.6 years
1.8 years
2.1 years
2.3 years
2.8 years
3.4 years
3.6 years
3.8 years
4.1 years
4.7 years
5.2 years
5.7 years
6.7 years
0
100
200
300
400
500
600
Alignment position

Figure S3

V.

M6_C2V5

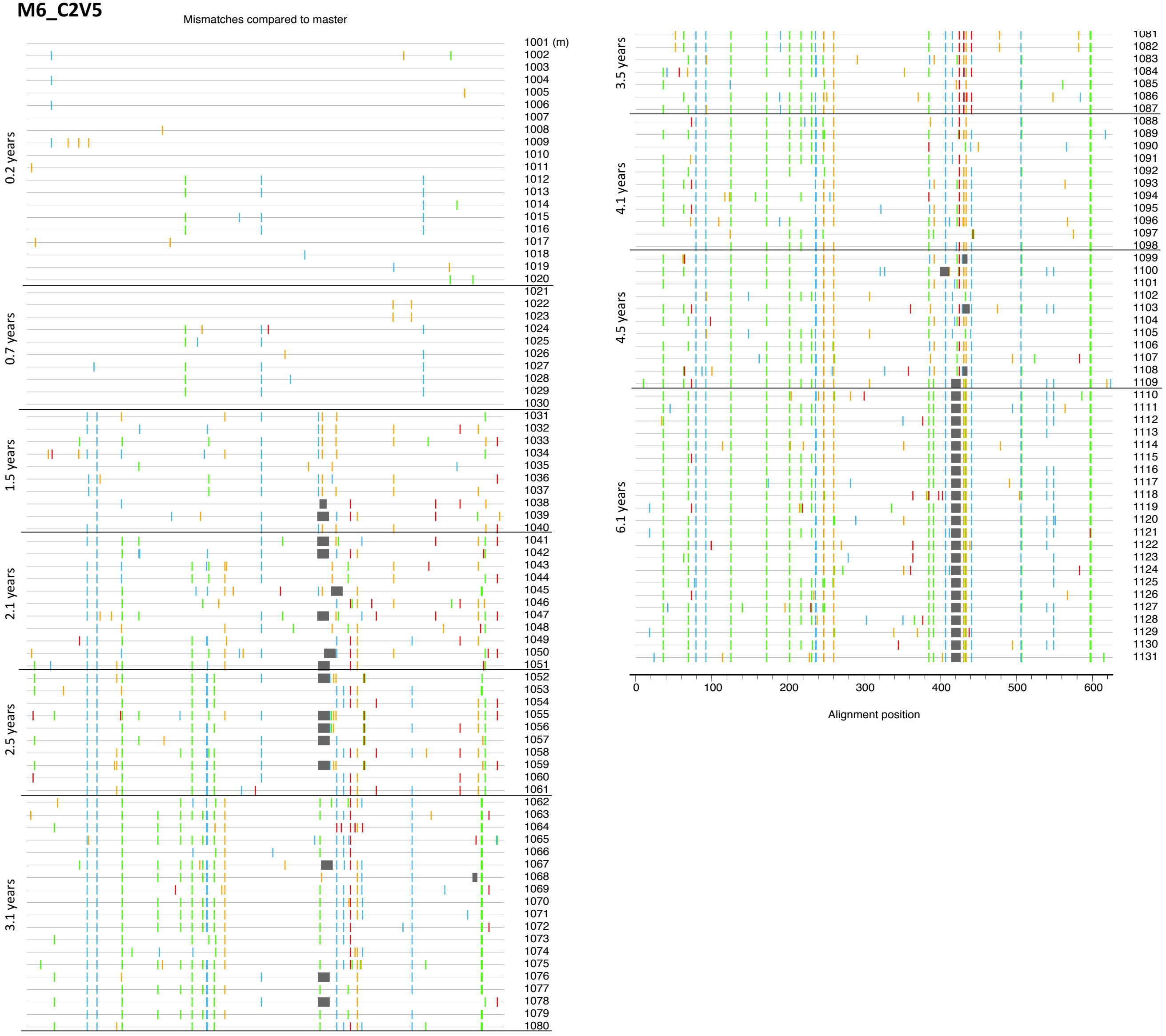

Figure S3
W.

M7_C2V5

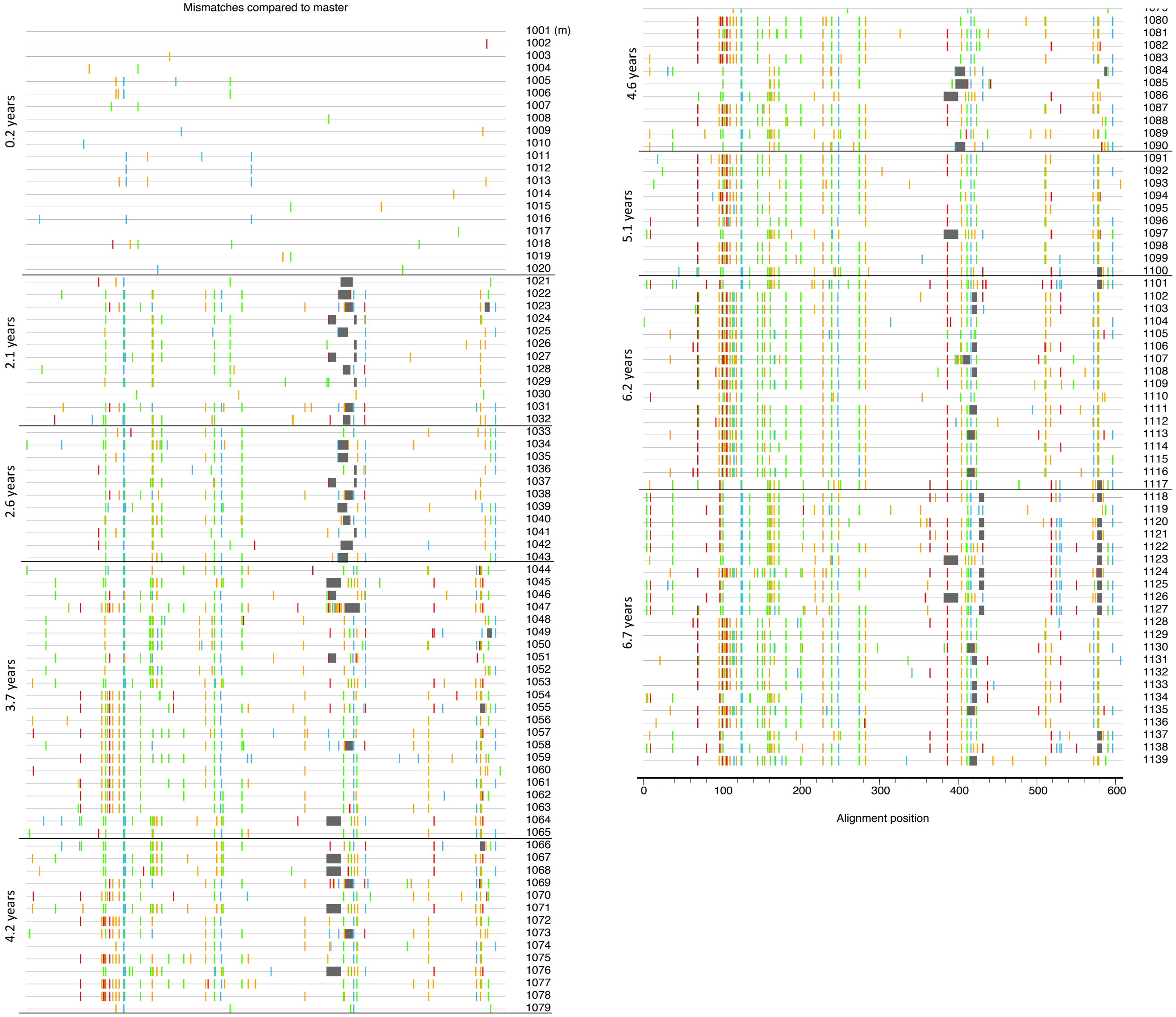

Figure S3

X.

M8_C2V5

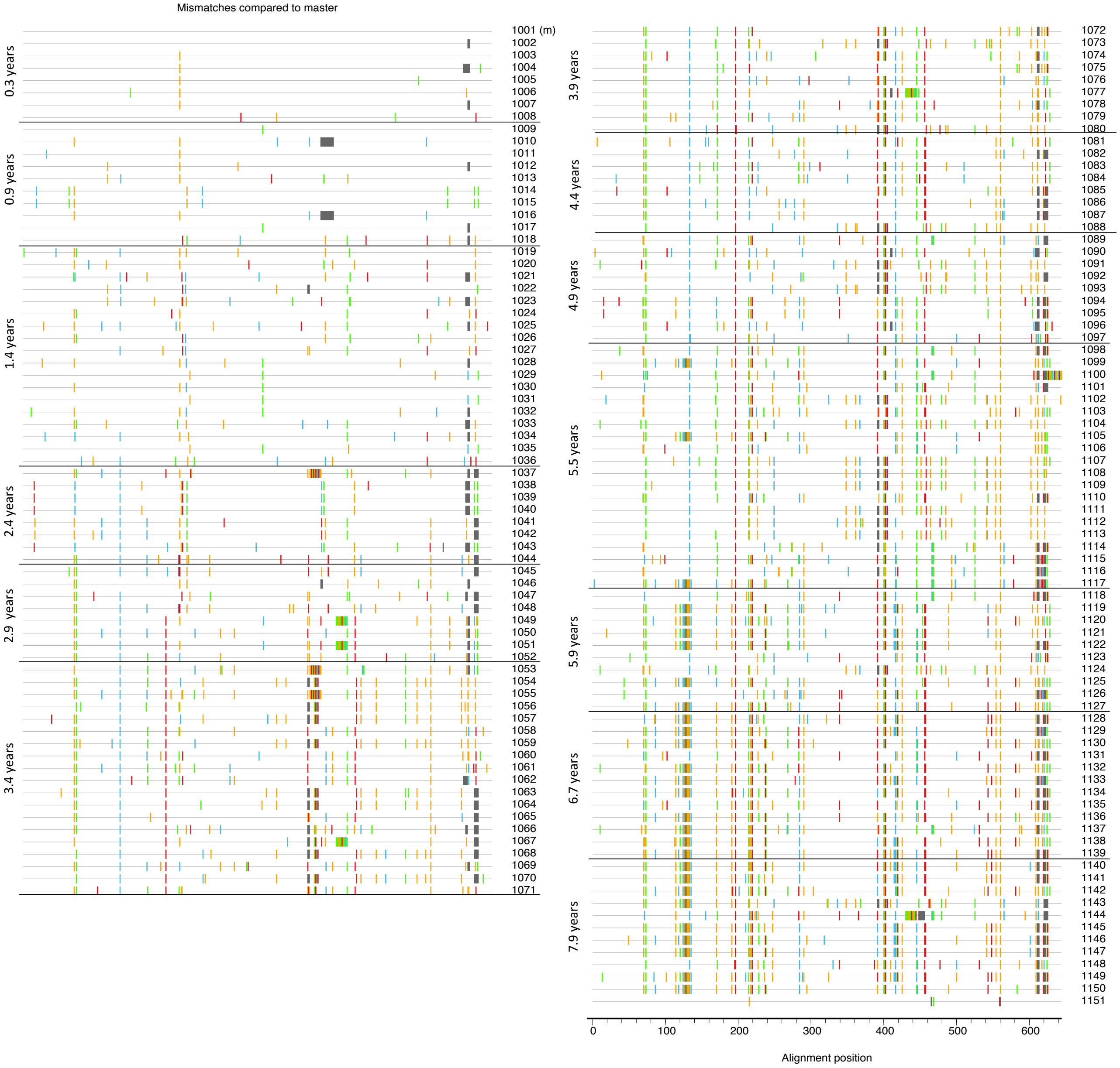

Figure S3

Y.

M9_C2V5

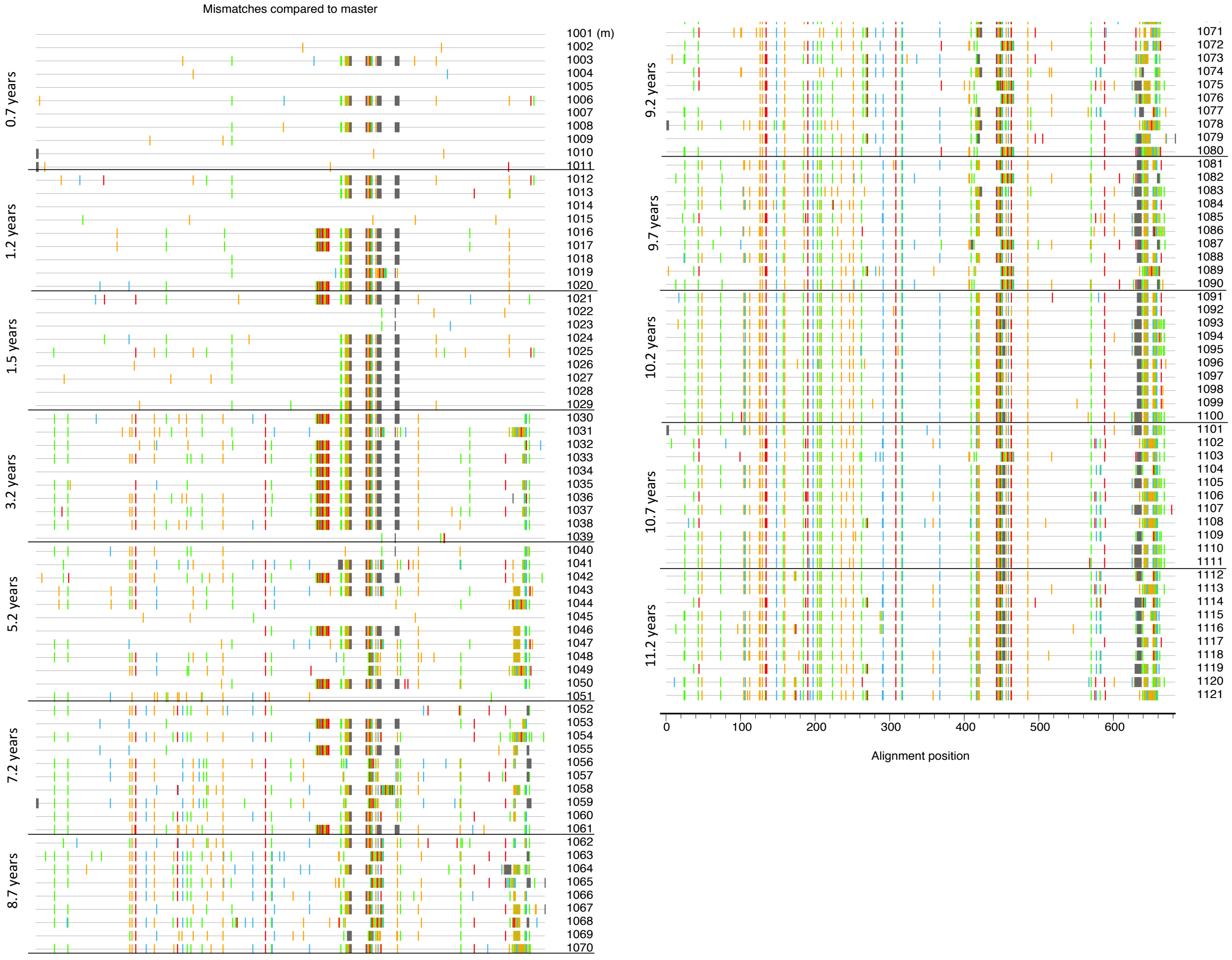

Figure S3

Z.

M10_C2V5

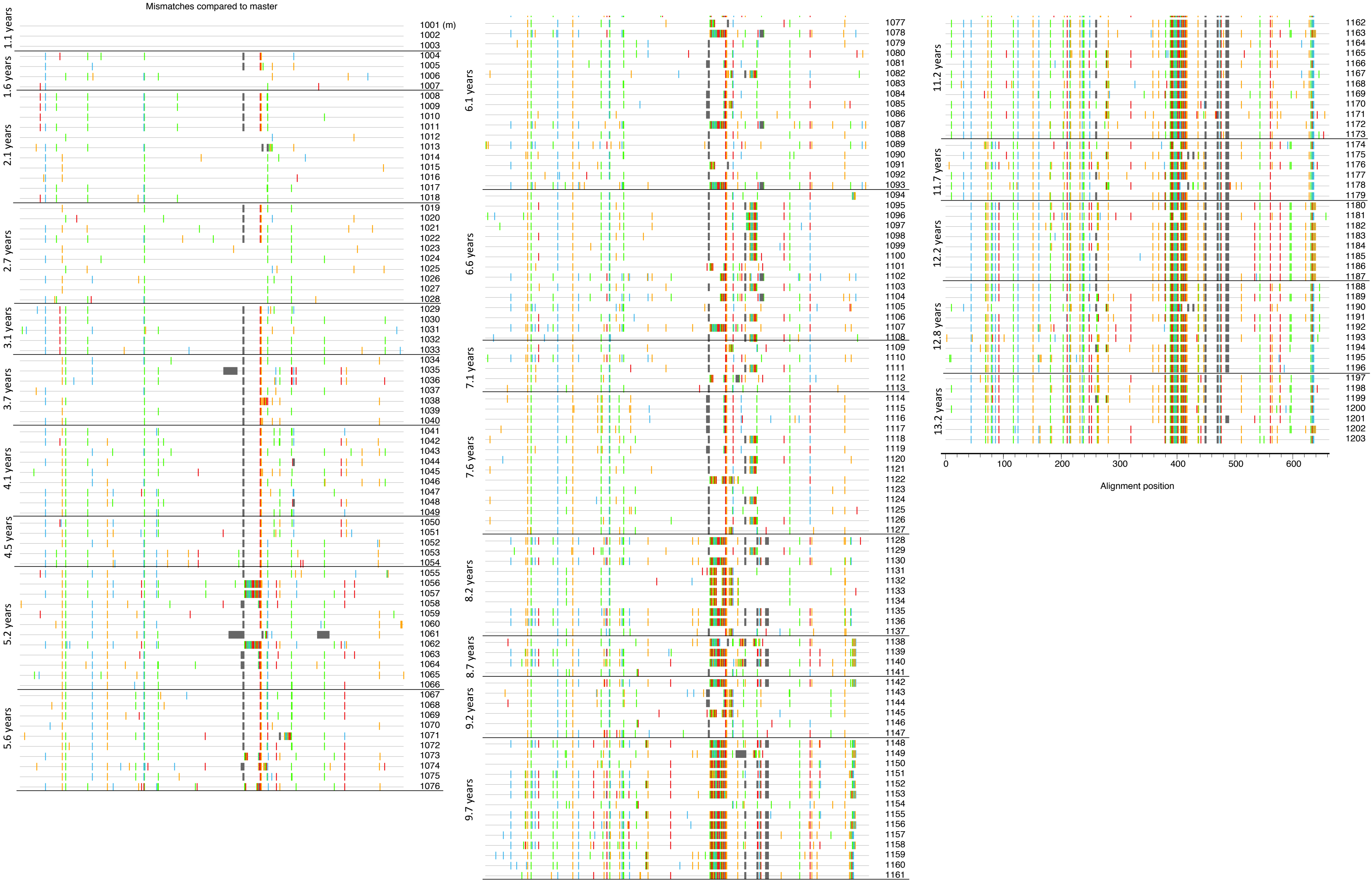

Figure S3
AA.

M11_C2V5

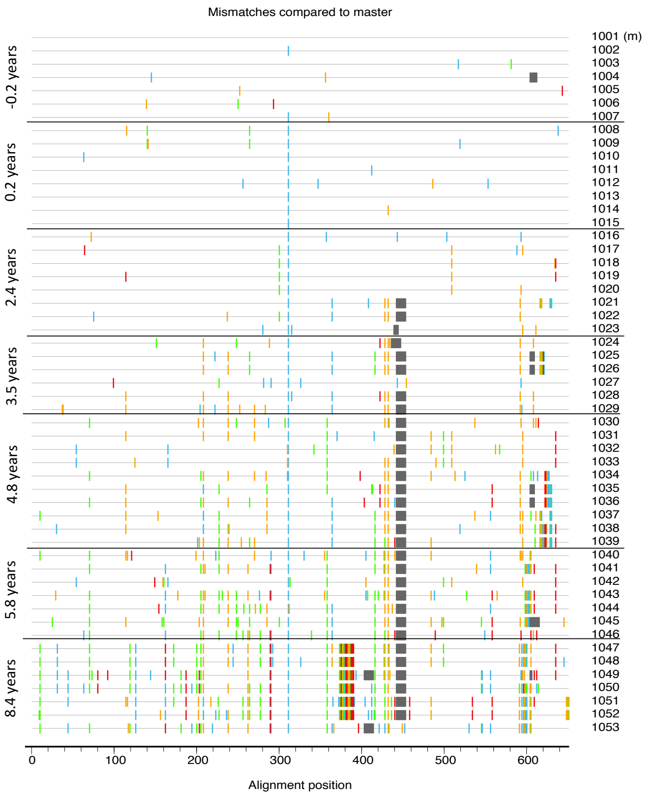

Supplement: S3 Fig — Alignments show nucleotide substitutions relative to the consensus sequence at first timepoint for gag and env-gp120 of the WIHS (A-N) and C2V5 of the MACS (O-Y). Substitutions relative to the first timepoint consensus (master) are color-coded: A = green, C = blue, G = orange, T = red, and grey = gap/deletion. Years post seroconversion is shown to the left of the denoted sequences. (PDF) [file pone.0182443.s003.pdf]
