# Supplementary material for: Patterns and rates of viral evolution in HIV-1 subtype B infected females and males
Source: PLoS One. 2017 Oct 18;12(10):e0182443. doi: 10.1371/journal.pone.0182443 (PMC5646779; doi:10.1371/journal.pone.0182443)
Supplement: S1 Table — (DOCX) [file pone.0182443.s014.docx]

**S1 Table. Comparison of nucleotide substitution rate estimates for constant and exponential growth population coalescent models.**

| **Viral region** | **PtID^a^** | **Constant^b^** | **Exponential growth^c^** |
| --- | --- | --- | --- |
| *gag* | F1 | 0.0073 [0.0061-0.0084] | 0.0066 [0.0054-0.0078] |
|  | F2 | 0.0081 [0.0064-0.0099] | 0.0058 [0.0047-0.0069] |
|  | F3 | 0.0067 [0.0053-0.0081] | 0.0065 [0.0052-0.0079] |
|  | F4 | 0.0079 [0.0064-0.0095] | 0.0063 [0.0051-0.0075] |
|  | F5 | 0.018 [0.014-0.022] | 0.016 [0.013-0.020] |
|  | F6 | 0.0051 [0.0041-0.0060] | 0.0052 [0.0044-0.0062] |
|  | F7 | 0.015 [0.012-0.017] | 0.0084 [0.0073-0.0096] |
|  | F8 | 0.011 [0.0085-0.013] | 0.0098 [0.0078-0.012] |
| *env-gp120* | F1 | 0.021 [0.019-0.024] | 0.020 [0.017-0.023] |
|  | F2 | 0.047 [0.038-0.057] | 0.047 [0.037-0.057] |
|  | F3 | 0.023 [0.020-0.027] | 0.021 [0.017-0.024] |
|  | F4 | 0.024 [0.020-0.028] | 0.021 [0.018-0.025] |
|  | F5 | 0.015 [0.012-0.018] | 0.016 [0.013-0.018] |
|  | F6 | 0.017 [0.015-0.019] | 0.018 [0.015-0.020] |
|  | F7 | 0.026 [0.022-0.032] | 0.021 [0.018-0.025] |
|  | F8 | 0.017 [0.015-0.020] | 0.014 [0.012-0.017] |
| *C2V5* | F1 | 0.021 [0.018-0.024] | 0.020 [0.017-0.023] |
|  | F2 | 0.051 [0.037-0.063] | 0.048 [0.036-0.060] |
|  | F3 | 0.015 [0.012-0.019] | 0.017 [0.013-0.020] |
|  | F4 | 0.020 [0.016-0.024] | 0.019 [0.015-0.022] |
|  | F5 | 0.010 [0.0079-0.013] | 0.012 [0.0093-0.014] |
|  | F6 | 0.025 [0.021-0.029] | 0.025 [0.021-0.030] |
|  | F7 | 0.019 [0.016-0.022] | 0.019 [0.016-0.022] |
|  | F8 | 0.020 [0.017-0.024] | 0.020 [0.016-0.023] |
|  | M1 | 0.014 [0.011-0.017] | 0.014 [0.012-0.017] |
|  | M2 | 0.020 [0.017-0.023] | 0.020 [0.017-0.022] |
|  | M3 | 0.022 [0.017-0.026] | 0.022 [0.017-0.027] |
|  | M4 | 0.024 [0.020-0.027] | 0.023 [0.020-0.027] |
|  | M5 | 0.039 [0.033-0.045] | 0.038 [0.033-0.045] |
|  | M6 | 0.019 [0.015-0.024] | 0.018 [0.015-0.023] |
|  | M7 | 0.013 [0.010-0.015] | 0.013 [0.011-0.015] |
|  | M8 | 0.024 [0.021-0.028] | 0.023 [0.020-0.027] |
|  | M9 | 0.011 [0.0093-0.014] | 0.010 [0.0083-0.012] |
|  | M10 | 0.015 [0.013-0.017] | 0.014 [0.012-0.017] |
|  | M11 | 0.016 [0.012-0.020] | 0.015 [0.011-0.018] |

^a^PtID signifies participant identifier.

^b^Nucleotide substitution rate (subs./site/year) estimated with a constant population coalescent tree prior.

^c^Nucleotide substitution rate (subs./site/year) estimated with an exponential growth population coalescent tree prior.
