# Supplementary material for: Patterns and rates of viral evolution in HIV-1 subtype B infected females and males
Source: PLoS One. 2017 Oct 18;12(10):e0182443. doi: 10.1371/journal.pone.0182443 (PMC5646779; doi:10.1371/journal.pone.0182443)
Supplement: S2 Table — (DOCX) [file pone.0182443.s015.docx]

**S2 Table. HIV *gag* and *env-gp120* sequences collected for analysis**

| **PtID^a^** | **Years**  **to SC^b^** | **ART^c^** | ***gag*** | ***env-gp120*** |  | **PtID** | **Years**  **to SC** | **ART** | ***gag*** | ***env-gp120*** |
| --- | --- | --- | --- | --- | --- | --- | --- | --- | --- | --- |
| F1 | 0.2 |  | 21 | 24 |  | F5 | 0.2 |  | 22 | 20 |
| (1996) | 0.7 |  | 20 | 23 |  | (1997) | 0.8 | ++ | 21 | 24 |
|  | 1.3 | ++ | 20 | 21 |  |  | 1.9 | ++ | 22 | 23 |
|  | 2.2 |  | 22 | 20 |  |  | 2.4 | ++ | 27 | 23 |
|  | 3.3 | ++ | 20 | 22 |  |  | 3.5 |  | 21 | 20 |
|  | 4.3 | ++ | 21 | 23 |  |  | 4.2 | ++ | 22 | 23 |
|  | 5.3 |  | 21 | 20 |  |  | 5.3 | ++ | 24 | 20 |
|  | 6.3 |  | 20 | 23 |  |  | 6.2 |  | 21 | 20 |
|  | 7.3 |  | 20 | 20 |  | F6 | 0.8 | +++ | 2 | 0 |
|  | 8.3 |  | 22 | 20 |  | (2000) | 1.2 | +++ | 5 | 0 |
|  | 9.3 |  | 4 | 39 |  |  | 2.0 |  | 21 | 19 |
|  | 10.3 |  | 26 | 20 |  |  | 2.3 |  | 23 | 23 |
|  | 11.3 | +++ | 23 | 21 |  |  | 3.2 |  | 22 | 16 |
| F2 | 0.3 |  | 23 | 22 |  |  | 4.2 |  | 23 | 25 |
| (1998) | 0.8 |  | 23 | 26 |  |  | 5.2 |  | 24 | 24 |
|  | 1.3 |  | 26 | 24 |  |  | 6.2 |  | 25 | 20 |
|  | 1.7 |  | 21 | 20 |  |  | 7.2 |  | 28 | 21 |
|  | 2.2 |  | 26 | 31 |  |  | 8.2 |  | 20 | 20 |
|  | 3.2 |  | 24 | 24 |  |  | 9.8 |  | 21 | 20 |
|  | 4.2 |  | 22 | 22 |  |  | 10.3 |  | 25 | 20 |
|  | 5.5 |  | 20 | 20 |  |  | 11.7 |  | 25 | 22 |
|  | 6.0 | +++ | 21 | 21 |  | F7 | 0.5 |  | 27 | 20 |
| F3 | 0.3 |  | 25 | 21 |  | (2001) | 1.0 |  | 21 | 22 |
| (2002) | 0.8 |  | 22 | 23 |  |  | 1.5 |  | 21 | 21 |
|  | 1.2 |  | 24 | 23 |  |  | 1.9 |  | 23 | 19 |
|  | 1.7 |  | 26 | 20 |  |  | 2.9 |  | 20 | 24 |
|  | 2.7 |  | 24 | 22 |  |  | 3.8 |  | 24 | 20 |
|  | 3.8 |  | 23 | 21 |  |  | 4.8 |  | 21 | 25 |
|  | 4.8 |  | 21 | 20 |  |  | 5.8 |  | 23 | 21 |
|  | 5.3 |  | 21 | 21 |  |  | 6.8 |  | 25 | 22 |
|  | 5.8 | +++ | 2 | 2 |  |  | 7.9 |  | 20 | 20 |
| F4 | 0.2 |  | 8 | 5 |  |  | 8.9 |  | 25 | 24 |
| (2002) | 0.7 |  | 3 | 7 |  |  | 9.9 |  | 22 | 22 |
|  | 1.2 |  | 22 | 23 |  |  | 10.8 |  | 20 | 24 |
|  | 1.7 |  | 11 | 4 |  | F8 | 0.2 |  | 22 | 20 |
|  | 2.7 |  | 24 | 29 |  | (1996) | 0.7 |  | 21 | 22 |
|  | 3.6 |  | 23^d^ | 23 |  |  | 1.2 |  | 26 | 21 |
|  | 4.6 |  | 21 | 20 |  |  | 2.2 | ++ | 20 | 22 |
|  | 5.6 |  | 21 | 21 |  |  | 5.3 |  | 21 | 20 |
|  | 6.6 |  | 22 | 23 |  |  | 5.9 |  | 25 | 20 |
|  | 7.6 |  | 21 | 22 |  |  | 8.0 |  | 22 | 24 |
|  | 8.1 | +++ | 21 | 22 |  |  | 8.9 |  | 21 | 21 |
|  |  |  |  |  |  |  | 9.9 |  | 23 | 20 |
|  |  |  |  |  |  |  |  |  |  |  |
|  |  |  |  |  |  | **median** |  |  | 22 | 21 |
|  |  |  |  |  |  | **minimum** |  |  | 2 | 0 |
|  |  |  |  |  |  | **maximum** |  |  | 28 | 39 |
|  |  |  |  |  |  |  |  |  |  |  |

^a^PtID signifies Participant Identifier (Year infected with HIV-1).

^b^Years SC is years post seroconversion.

^c^ART shows the number of prescribed antiretroviral drugs, signified by ‘+’.

^d^Twenty-four sequences, including one duplicated sequence, was erroneously included in subsequent analyses.
