# Supplementary material for: Patterns and rates of viral evolution in HIV-1 subtype B infected females and males
Source: PLoS One. 2017 Oct 18;12(10):e0182443. doi: 10.1371/journal.pone.0182443 (PMC5646779; doi:10.1371/journal.pone.0182443)
Supplement: S3 Table — (DOCX) [file pone.0182443.s016.docx]

**S3 Table. Rate estimations using a Bayesian phylogenetic approach.**

|  |  | *gag* |  |  | *env-gp120* |  |  | *C2V5* |  |
| --- | --- | --- | --- | --- | --- | --- | --- | --- | --- |
| PtID^a^ | Subs. rate^b^ | Coeff. of rate var.^c^ | Covar. of rates^d^ | Subs. rate^b^ | Coeff. of rate var.^c^ | Covar. of rates^d^ | Subs. rate^b^ | Coeff. of rate var. ^c^ | Covar. of rates^d^ |
| F1 | 0.0066  [0.0055-0.0078] | 0.53  [0.38-0.70] | 0.0075  [-0.071-0.093] | 0.020  [0.017-0.023] | 0.55  [0.43-0.67] | 0.034  [-0.050-0.12] | 0.020  [0.017-0.023] | 0.54  [0.38-0.70] | 0.026  [-0.058-0.11] |
| F2 | 0.0058  [0.0047-0.0069] | 0.18  [0.0000077-0.37] | 0.0013  [-0.091-0.10] | 0.047  [0.037-0.057] | 0.94  [ 0.73-1.15] | 0.058  [-0.041-0.17] | 0.048  [0.036-0.060] | 1.09  [0.79-1.46] | 0.047  [-0.056-0.16] |
| F3 | 0.0065  [0.0052-0.0079] | 0.16  [0.00021-0.34] | -0.0029  [-0.11-0.097] | 0.021  [0.017-0.024] | 0.54  [0.40-0.67] | 0.022  [-0.084-0.13] | 0.017  [0.013-0.020] | 0.53  [0.32-0.74] | 0.0082  [-0.098-0.11] |
| F4 | 0.0063  [0.0051-0.0075] | 0.32  [0.12-0.53] | 0.0017  [-0.099-0.097] | 0.021  [0.018-0.025] | 0.38  [0.24-0.51] | 0.020  [-0.080-0.12] | 0.019  [0.015-0.022] | 0.22  [0.00024-0.41] | 0.0031  [-0.095-0.10] |
| F5 | 0.0160  [0.013-0.020] | 0.57  [0.40-0.75] | 0.018  [-0.087-0.12] | 0.016  [0.013-0.018] | 0.54  [0.39-0.70] | 0.016  [-0.084-0.12] | 0.012  [0.0093-0.014] | 0.53  [0.28-0.78] | 0.021  [-0.089-0.12] |
| F6 | 0.0053  [0.0044-0.0062] | 0.36  [0.20-0.52] | 0.0046  [-0.080-0.091] | 0.018  [0.015-0.020] | 0.84  [0.68-0.99] | 0.078  [-0.026-0.19] | 0.025  [0.021-0.030] | 0.99  [0.78-1.21] | 0.068  [-0.026-0.16] |
| F7 | 0.0084  [0.0073-0.0096] | 0.20  [0.043-0.33] | -0.0006  [-0.083-0.079] | 0.021  [0.018-0.025] | 0.58  [0.47-0.69] | 0.041  [-0.043-0.12] | 0.019  [0.016-0.022] | 0.78  [0.61-0.96] | 0.054  [-0.033-0.14] |
| F8 | 0.0098  [0.0078-0.012] | 0.34  [0.15-0.53] | 0.0085  [-0.085-0.11] | 0.014  [0.012-0.017] | 0.81  [0.63-0.99] | 0.043  [-0.059-0.15] | 0.020  [0.016-0.023] | 0.54  [0.36-0.72] | 0.017  [-0.085-0.12] |
| **Median** | **0.0065** | **0.33** | **0.0032** | **0.020** | **0.57** | **0.038** | **0.019** | **0.54** | **0.024** |
| M1 |  |  |  |  |  |  | 0.014  [0.012-0.017] | 0.91  [0.65-1.19] | 0.048  [-0.075-0.17] |
| M2 |  |  |  |  |  |  | 0.020  [0.017-0.022] | 0.97  [0.79-1.19] | 0.028  [-0.060-0.12] |
| M3 |  |  |  |  |  |  | 0.022  [0.017-0.027] | 0.85  [0.56-1.15] | 0.076  [-0.069-0.21] |
| M4 |  |  |  |  |  |  | 0.023  [0.012-0.027] | 0.81  [0.66-0.99] | 0.042  [-0.048-0.13] |
| M5 |  |  |  |  |  |  | 0.038  [0.033-0.045] | 0.66  [0.50-0.82] | 0.019  [-0.070-0.11] |
| M6 |  |  |  |  |  |  | 0.018  [0.015-0.023] | 0.77  [0.51-1.02] | 0.045  [-0.081-0.17] |
| M7 |  |  |  |  |  |  | 0.013  [0.011-0.015] | 0.80  [0.61-1.01] | 0.046  [-0.076-0.17] |
| M8 |  |  |  |  |  |  | 0.023  [0.012-0.027] | 0.56  [0.40-0.73] | 0.014  [-0.095-0.13] |
| M9 |  |  |  |  |  |  | 0.010  [0.0083-0.012] | 0.78  [0.58-0.99] | 0.047  [-0.073-0.19] |
| M10 |  |  |  |  |  |  | 0.014  [0.012-0.017] | 0.56  [0.42-0.71] | 0.023  [-0.078-0.12] |
| M11 |  |  |  |  |  |  | 0.015  [0.011-0.018] | 0.49  [0.21-0.75] | 0.024  [-0.17-0.22] |
| **Median** |  |  |  |  |  |  | **0.018** | **0.78** | **0.042** |
| *Difference WIHS vs. MACS* | |  |  |  |  |  | 0.001 | 0.24 | 0.018 |
| *(P-value)*^e^ |  |  |  |  |  |  | 0.492 | 0.319 | 0.506 |

^a^PtID = Participant Identifier; ^b^Intra-host mean viral substitution rate (substitutions/site/year). Posterior means and 95% HPD are shown; ^c^Coefficient of variation of the branch-specific rates. Posterior means and 95% HPD are shown; ^d^Covariance of the branch-specific rates. Posterior means and 95% HPD are shown; ^e^Determined by Mann-Whitney U test.
